# From Melt Miscibility to Crystallization: Rheological and Thermal Analysis of Polyethylene Blends for Recycling Applications

**DOI:** 10.3390/polym18172054

**Published:** 2026-08-24

**Authors:** Nicole R. Demarquette, Judith Zaessinger, Daria Strugova, Amin Mirzadeh, Eunse Chang, Céline T. Bellehumeur

**Affiliations:** 1 Mechanical Engineering Department, École de Technologie Supérieure, Montréal, QC H3C 1K3, Canada; judith.zaessinger.1@ens.etsmtl.ca (J.Z.); daria.strugova@etsmtl.ca (D.S.); 2 Borouge International, 250 5 Street SW, Calgary, AB T2P 0R4, Canada; amin.mirzadeh@novachem.com (A.M.); eunse.chang@borougeinternational.com (E.C.); celine.bellehumeur@borougeinternational.com (C.T.B.)

**Keywords:** rheology, thermodynamics, circularity of polyethylene

## Abstract

Plastic waste management remains a major challenge, and the mechanical recycling of polyethylene (PE) is complicated by the coexistence of HDPE, LDPE, and LLDPE grades that are difficult to separate. This review critically examines the miscibility, apparent miscibility, compatibility, and crystallization behavior of PE blends, with an emphasis on linear viscoelastic rheology and thermal analysis. Rheological criteria, including additivity, Cole–Cole, time–temperature superposition, Han, and van Gurp–Palmen analyses, were evaluated, together with DSC and successive self-nucleation and annealing. Most studies indicate that HDPE/LDPE blends are melt-immiscible. HDPE/LLDPE blends more frequently exhibit apparent melt miscibility when LLDPE has low short-chain branching, typically below 15 branches per 1000 carbon atoms, and relatively low molar mass; increasing molar mass generally promotes immiscibility. LDPE/LLDPE behavior is less systematic and depends on branch content and distribution, molar mass, and catalyst-controlled molecular architecture. Crystallization does not necessarily mirror melt miscibility. HDPE/LLDPE co-crystallization is generally favored at high HDPE contents but strongly depends on branching, comonomer type, and cooling conditions, while LDPE/LLDPE may co-crystallize despite apparent melt immiscibility. These trends provide practical guidelines for designing recycled PE blends with more predictable processing and properties.

## 1. Introduction

Waste management has become a major societal challenge, with plastic accumulation threatening ecosystems and human health. Although plastic circularity is increasingly promoted through regulatory and industrial initiatives, only a small fraction of plastic waste is currently recycled [1,2,3,4]. The recovery and reuse of polyethylene (PE) are particularly urgent because PE is extensively used in packaging and single-use products, and post-consumer recycled PE (PCR PE) often contains mixtures of different PE grades, such as low-density polyethylene (LDPE), high-density polyethylene (HDPE), and linear low-density polyethylene (LLDPE), which are difficult to separate due to their similar densities and chemical structures [4,5,6,7].

Mechanical recycling, which generally involves sorting, cleaning, and re-extruding plastic waste, often with the addition of virgin polymer, is one of the most accessible strategies to increase the use of PCR PE. However, this approach remains limited by the differences in molecular structure among PE grades, as well as by residual impurities and possible degradation during repeated processing, which can lead to phase separation, material inconsistency, and downcycling. As a result, mechanical recycled streams are often complex blends containing different PE grades, degraded PE fractions and contaminants.

Within blends, two main types exist: those composed of miscible polymers and those composed of immiscible polymers. Miscible polymers form a “molecular-level solution” and typically exhibit properties that vary as a function of their composition [8]. They are also characterized by a negative free energy of mixing. However, because of their high molar mass and low entropy of mixing, most polymer pairs are thermodynamically immiscible and form multiphase materials. In such systems, the final properties depend strongly on morphology which include dispersed droplet in a matrix, co-continuous, or lamellar morphologies, each affecting the final properties of the material. For polyethylene blends, the terms miscibility, compatibility, and apparent miscibility must, therefore, be used carefully, because the conclusion may depend on the scale of observation and on the experimental method used.

Rheology, in particular small amplitude oscillatory shear (SAOS), is a very valuable tool to assess the apparent miscibility of two polymers in the molten state [9]. Indeed, it probes the linear viscoelastic behavior which reflects the relaxation dynamics of polymer chains. It can, therefore, indicate whether the blend components relax cooperatively or whether an additional relaxation, suggesting phase separation occurs. Rheological analysis can, thus, suggest apparent melt miscibility, but cooperative relaxation should be considered a necessary but not sufficient condition for thermodynamic miscibility. Rheological analysis can, therefore, provide important information on the apparent phase behavior of polyethylene blends, while requiring complementary interpretation.

After blending, cooling is particularly important because PE is semicrystalline, and crystallization behavior depends on melt-state-apparent miscibility, blend composition, cooling rate, and processing conditions, including whether solidification occurs under shear or extension. Single-phase blends may co-crystallize or phase separate during cooling, whereas multiphase blends generally crystallize separately, potentially modifying the morphology developed in the melt. In all cases, the final properties of PE blends are strongly influenced by structural factors such as degree of crystallinity, spherulite size, lamellar thickness, and phase morphology. Therefore, understanding how melt-state interactions, crystallization, and processing conditions control morphology is essential for improving the quality and consistency of recycled PE blends.

**Figure 1:**
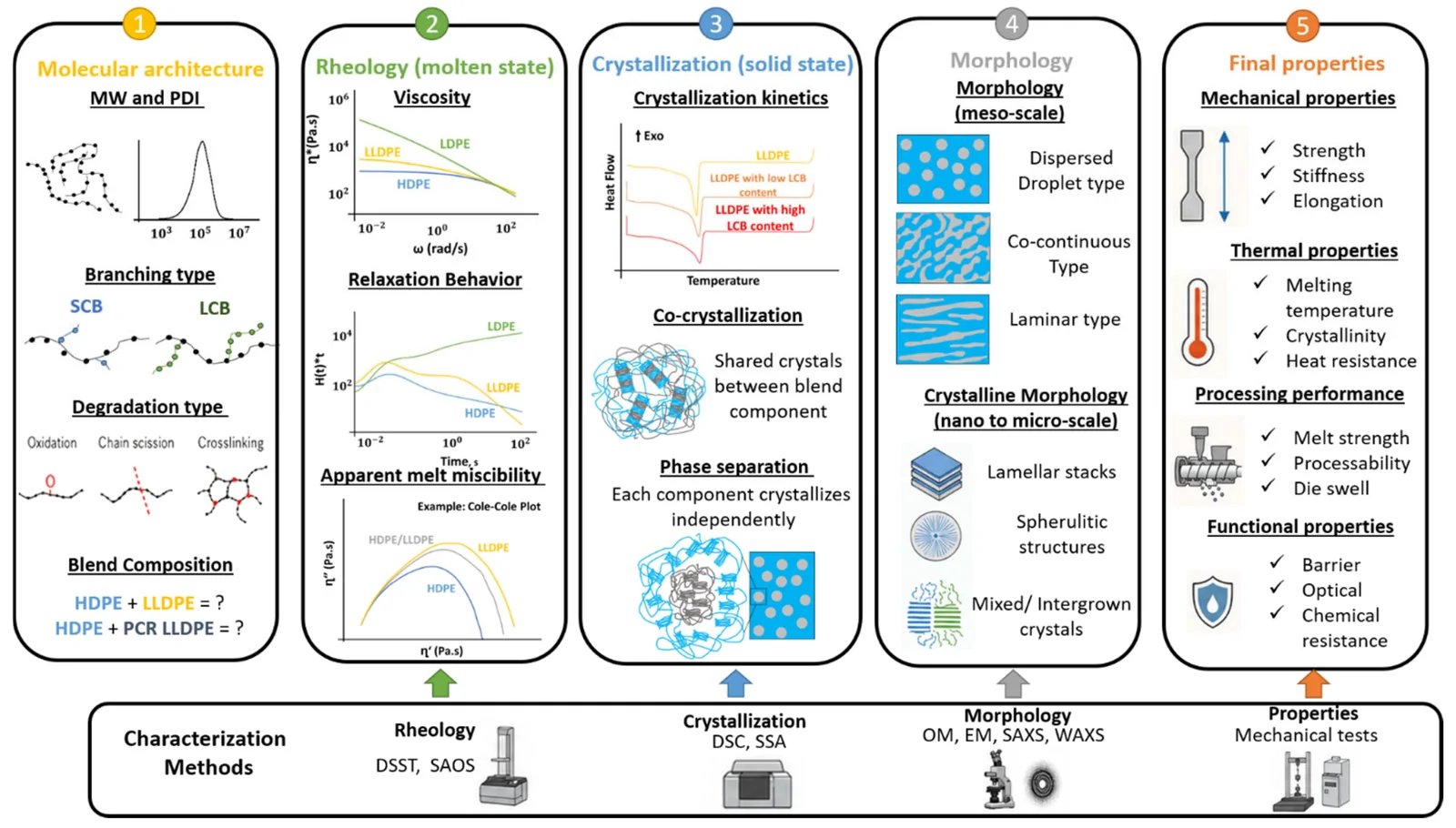
Conceptual framework showing how blend composition, melt state miscibility, cooling rate, and processing conditions affect morphology and consequently the functional properties of polyethylene blends (please note that the concepts of miscible and immiscible are a bit simplistic, as miscible correspond to a thermodynamic term and depends on the scale of observation).

Building on the framework presented in Figure 1, this review provides an expert-driven critical synthesis of the relationships among molecular architecture, apparent melt miscibility, crystallization, morphology, and final properties, while highlighting contradictions, methodological limitations, and remaining knowledge gaps in the field. As illustrated in Figure 1, differences in molar mass, molecular weight distribution, short- and long-chain branching, degradation state, and composition influence the melt-state rheological behavior of the components and their blends, including relaxation dynamics, apparent miscibility, interfacial behavior, and phase morphology. During cooling, these characteristics also affect crystallization kinetics, co-crystallization, phase separation, and the development of crystalline and phase morphologies, which ultimately contribute to the processing behavior and mechanical and functional properties of the blends.

Unlike many previous reviews, which focus either on melt-state rheology or on crystallization behavior [10,11] or technologies for recycling polyethylene [12], the present work considers the entire sequence from melt state interactions to crystallization and solid state structure. It not only summarizes the reported findings on miscibility and co-crystallization, but also critically evaluates the ability and limitations of the rheological and thermal techniques used to assess these phenomena. To the best of our knowledge, such a critical assessment of the available characterization methods has not previously been presented in the literature. Furthermore, contradictory findings are examined and interpreted considering differences in molecular architecture, blend composition and experimental conditions. This integrated analysis provides a practical framework for guiding the formulation of post-consumer recycled polyethylene (PCR-PE)/virgin PE blends.

## 2. Rheological Methods to Study Blend Homogeneity

### 2.1. Background

Small-amplitude oscillatory shear (SAOS) is widely used to probe the linear viscoelastic behavior of polymer blends in the molten state. In this regime, the storage modulus, loss modulus, complex viscosity and phase angle provide information on relaxation dynamics, molecular architecture, and possible phase behavior. For polyethylene blends, this analysis is particularly useful because differences in molecular weight, molecular weight distribution, short- and long-chain branching, and comonomer content strongly affect the rheological response.

Rheology can, therefore, provide evidence of apparent melt miscibility or compatibility, although it should not be considered a direct proof of thermodynamic miscibility. For example, when the storage modulus of a blend exceeds that of both neat components, particularly at low frequency, this is often interpreted as the signature of an additional relaxation process associated with phase separation or dispersed-phase deformation [13,14,15,16]. Such behavior has been reported for several polyethylene blends and has motivated the use of linear viscoelastic criteria to assess blend homogeneity, and several methods have, therefore, been proposed to analyze linear viscoelastic data and infer apparent miscibility, compatibility or phase separation in polymer blends. To ensure consistent terminology throughout this review, Table 1 presents the definitions of miscibility, apparent miscibility, and compatibility as applied to polymer blends.

The rheological methods that can be used to access apparent miscibility are reviewed below.

### 2.2. Analyses of Linear Viscoelasticity of Blends

#### 2.2.1. Additivity Rule

Researchers initially considered an additivity rule. According to this empirical rule a given rheological property of a blend formed by two polymers A and B, is compared to the one that would be obtained using an additivity rule, R_ar_ using the following equation:

(1) $${log(R}_{ar})=\phi_{A}\log\left(R_{A}\right)+\phi_{B}\log\left(R_{B}\right),$$

where $\emptyset_{i}$ and R_i_ are the volume concentration and rheological property of component i.

If the rheological properties of the blend formed by the two polymers A and B show a positive deviation behavior, it is because the polymers are immiscible. Conversely, if the rheological behavior of the blend formed by the two polymers A and B shows a negative deviation behavior, it is because the polymers are apparently miscible.

The additivity rule was applied in the literature to test the apparent miscibility of different types of polyethylene, utilizing zero shear viscosity [10,17,18,19,20], the storage modulus at low frequencies [17], relaxation time [19] or first normal stress coefficient [17]. These studies are discussed further in the paper.

#### 2.2.2. Cole–Cole Plot

The SAOS data can also be represented using Cole–Cole plot which was originally developed for dielectric relaxation and later adapted to describe the dynamic mechanical behavior of polymers [11,17,19]. In rheology, the Cole–Cole plot is commonly constructed by plotting the loss modulus (G″) as a function of the storage modulus (G′), or equivalently by plotting the imaginary and real part of the complex viscosity. For a rheologically simple material described by a single Maxwell element, the Cole–Cole takes the form of a semicircle. Materials with several discrete relaxation processes, result in a Cole-Cole plot with multiple arcs, whereas materials with a broad or continuous distribution of relaxation times generally result in plots with flattened or skewed arc. More details about Maxwell models and viscoelastic properties of polymers can be found in the monograph of Ferry [21].

**Figure 2:**
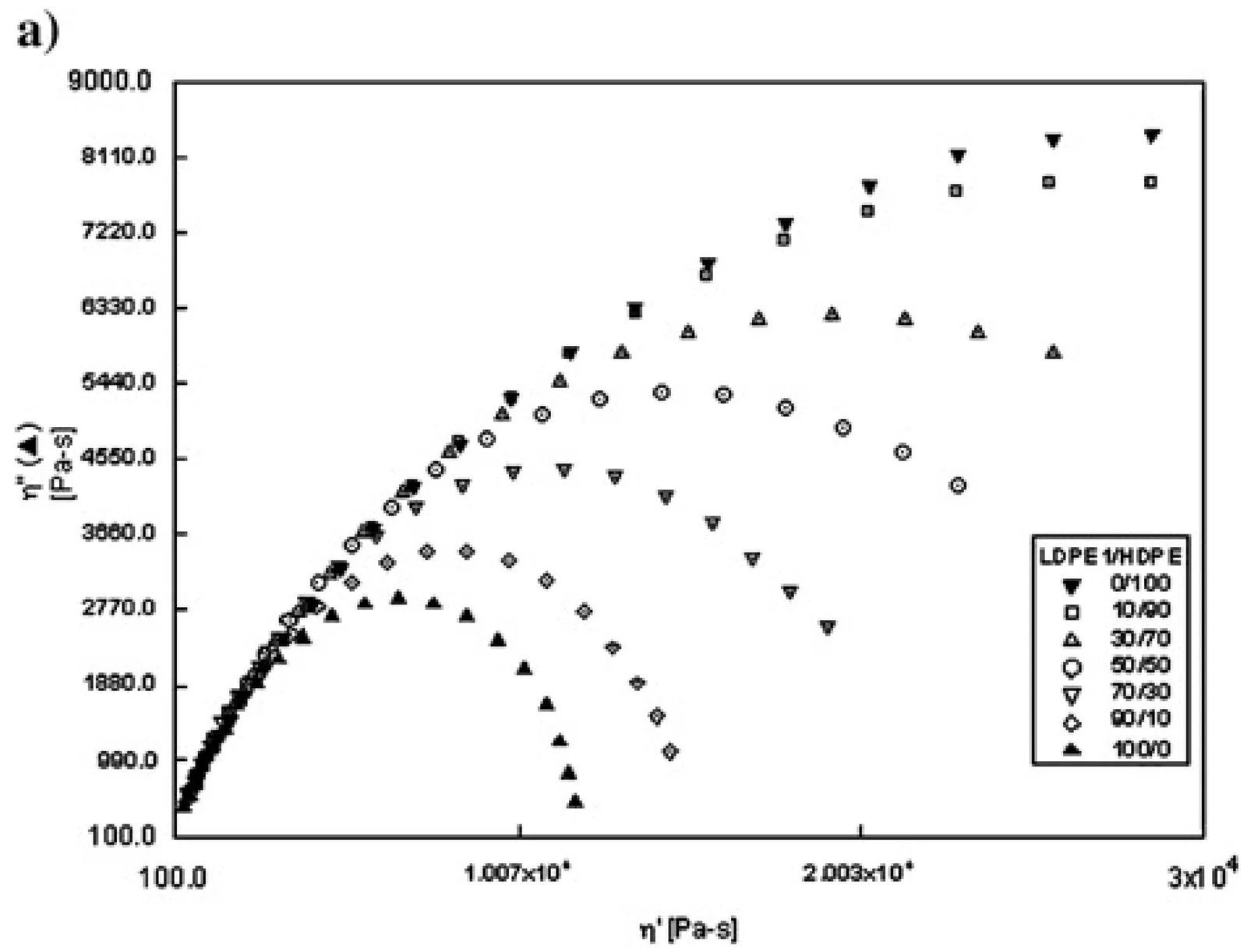

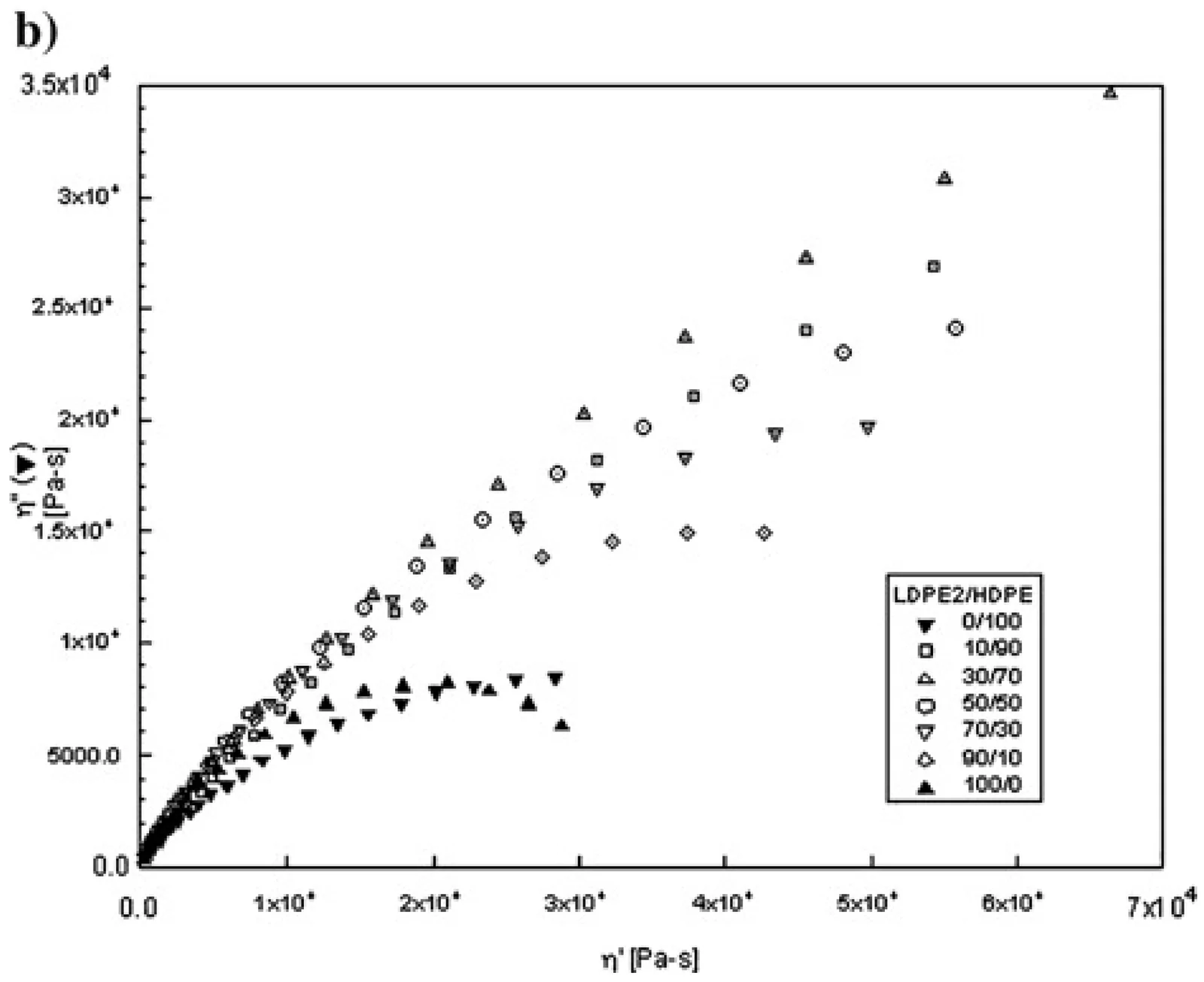
Cole–Cole plots for (**a**) LDPE1/HDPE and (**b**) LDPE2/HDPE (Tmix = 190 °C, Ttest = 190 °C, amplitude = 15%). Specifications of the polymer given in Table 3. Reprinted with permission from Hameed and Hussein [17]. Copyright © 2004 WILEY-VCH Verlag GmbH & Co. KGaA, Weinheim.

Cole–Cole plots have been used to assess apparent miscibility or compatibility in polymer blends. When the blend curves evolve progressively within the range defined by the two neat components, as observed for the LDPE1/HDPE blends in Figure 2a [17], this behavior is often interpreted as indicating that no clearly distinguishable additional relaxation is detected within the experimental window. By contrast, the non-monotonic evolution and stronger deviations observed for several LDPE2/HDPE compositions in Figure 2b may indicate a more complex relaxation response associated with melt heterogeneity or phase separation. However, this criterion remains qualitative because there is no universally accepted quantitative definition of whether a blend curve lies sufficiently “between” those of the neat components. Moreover, Cole–Cole plots remove the explicit frequency dependence of the moduli and provide limited direct information on morphology or interfacial relaxation. They should, therefore, be used together with other rheological, thermal, and morphological analyses and should not be considered definitive evidence of thermodynamic miscibility or immiscibility.

For immiscible blends, models such as those of Palierne, Bousmina, or YZZ are more appropriate to relate rheological behavior to droplet-matrix or co-continuous morphologies [13,14,15,16]. Therefore, Cole–Cole plots should be considered a qualitative indicator of relaxation behavior, rather than a definitive criterion for thermodynamic miscibility.

#### 2.2.3. Time–Temperature Superposition, Han and Van Gurp and Palmen Plots

The time–temperature superposition principle (TTS) is commonly used to extend the frequency range of linear viscoelastic data by shifting measurements obtained at different temperatures to construct a master curve at a chosen reference temperature [9,21,22,23]. This approach is valid when all relaxation mechanisms are affected similarly by temperature; the material is then considered thermorheologically simple. The applicability of TTS can be further evaluated using complementary representations such as Han plots and van Gurp–Palmen plots.

Han plots are based on the tube model theory developed by Doi and Edward [24] for linear, flexible, entangled monodisperse homopolymers a temperature-independent relationship when plotting log G′ as a function of Log G″. Similarly, the van Gurp–Palmen plot [25] represents the loss angle as a function of the magnitude of the complex modulus, If data obtained at different temperatures superpose in these representations, the material is generally considered thermorheologically simple.

Because (TTS) implies that the relaxation mechanisms in a material have similar temperature dependence, it has often been used as an indirect criterion to assess apparent miscibility or compatibility in polymer blends. The underlying assumption is that, if both polymers relax cooperatively under temperature variations, the blend may be homogeneous in the molten state and the polymers they may be miscible. However, this interpretation must be treated with caution. Successful TTS does not constitute proof of thermodynamic miscibility, since immiscible blends may also follow TTS when the components have similar activation energies or temperature-dependent relaxation behavior. Conversely, failure of TTS may suggest phase separation or thermorheological complexity, but it should not be interpreted alone as definitive evidence of immiscibility [25].

**Figure 3:**
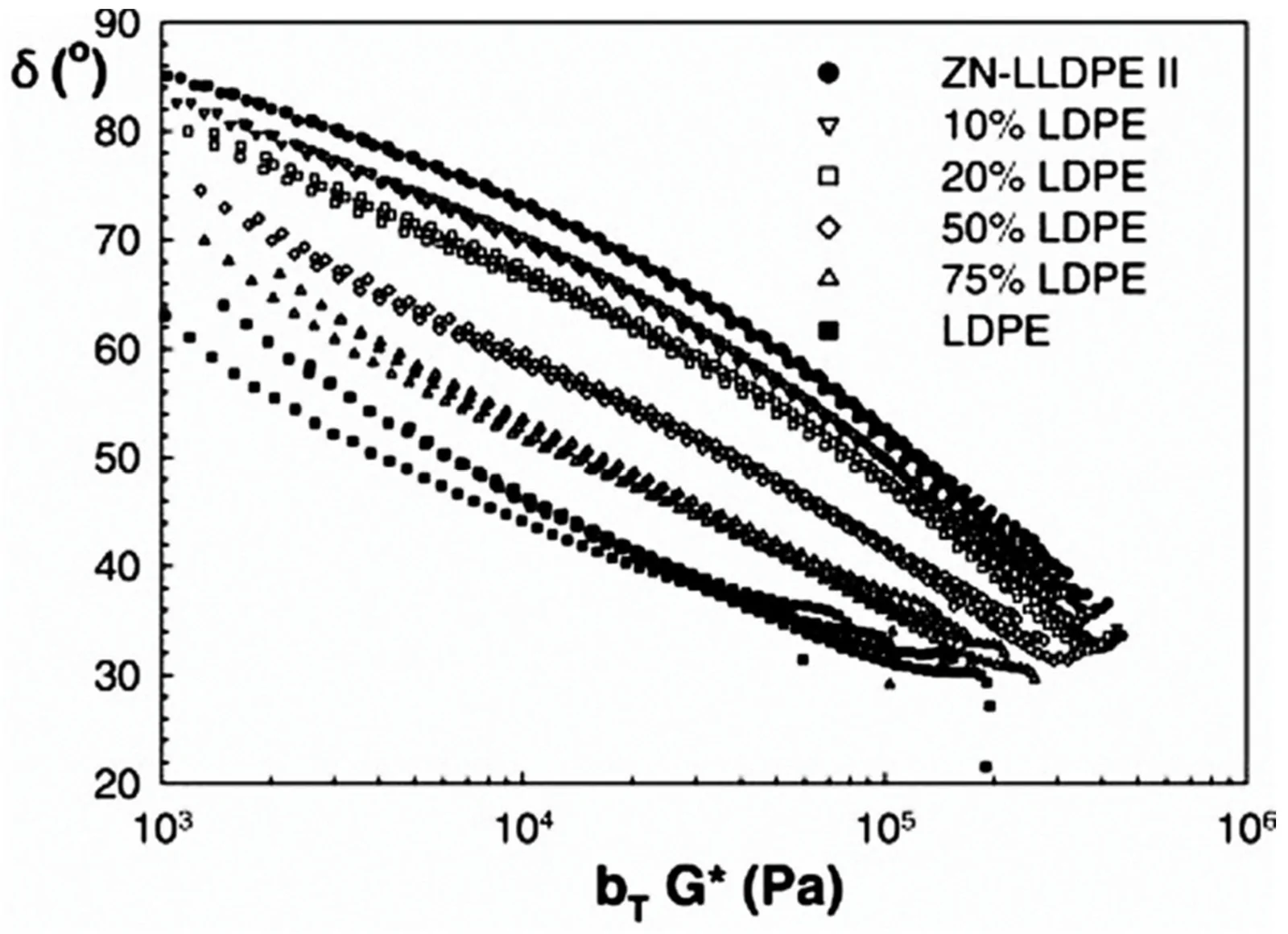
Van Gurp plot for an LLDPE/LDPE blend using linear viscoelastic data at 130, 150, 170, 190 and 210 °C. Reprinted with permission from [26]. Copyright © 2008 Wiley Periodicals, Inc. [26].

Figure 3 illustrates this issue for an LLDPE/LDPE blend system, using Van Gurp–Palmen plots constructed from linear viscoelastic data measured between 130 and 210 °C [26].

In this study, master curves were obtained for LLDPE and blends containing up to 50% LDPE, whereas blends with higher LDPE contents did not follow TTS. The authors attributed this failure of TTS to the immiscibility of the blend components in the molten state. This interpretation is reasonable, but the converse should not be assumed: the fact that a blend follows TTS does not necessarily prove miscibility. This example highlights the need to corroborate TTS-based interpretations with additional rheological, thermal or morphological evidence before drawing conclusions about blend compatibility.

Han plots have also been used in the literature to assess the apparent miscibility of different types of polyethylene blends [10,11,27]. In several studies, however, Han plots were constructed at a single temperature across various blend compositions. This use should be interpreted cautiously, because the original purpose of the Han plot is to assess the thermorheological behavior using data collected at different temperatures. Single-temperature Han plots may still provide useful information on composition-dependent relaxation behavior, but they should not be treated as definitive miscibility criteria.

#### 2.2.4. Palierne, Bousmina and YZZ Models

Other analyses have been proposed to evaluate the rheological behavior of polymer blends, particularly in cases where the components are thermodynamically immiscible. These models are typically used to describe the rheological response of immiscible polymer blends in order to characterize their morphology and interfacial tension [13,14,15,16]. More detailed discussions of these models can be found in Demarquette [28] and Strugova et al. [29,30].

#### 2.2.5. Conclusion

Table 2 presents a summary of the analyses that can be carried out to evaluate the apparent phase behavior of blends using linear viscoelastic measurements. These methods should not be considered equivalent or definitive criteria for thermodynamic miscibility. Rather, they provide rheological signatures that must be interpreted together with molecular architecture, composition, processing history and, when possible, complementary structural or thermal characterization.

### 2.3. Rheological Behavior of Polyethylene Blends

Before analyzing rheological studies that use small amplitude oscillatory shear (SAOS) to infer the apparent miscibility or compatibility of polyethylene blends, it is important to recall that the rheological response of PE is strongly governed by molecular architecture, particularly molecular weight, molecular weight distribution and the degree and type of branching.

High-density polyethylene (HDPE) is predominantly linear and, therefore, generally displays terminal flow behavior in the linear viscoelastic regime, with a storage modulus (G′) scaling approximately with ω^2^ and a loss modulus (G″) scaling approximately with ω at low frequencies. In contrast, low-density polyethylene (LDPE), which contains a significant number of long-chain branches, exhibits deviations from this ideal behavior. The long branches increase the relaxation time, resulting in a flattening of both modulus slopes at low frequencies. This also leads to an increase in zero-shear viscosity and more pronounced shear-thinning behavior.

Table 3, Table 4, Table 5, Table 6, Table 7, Table 8, Table 9 and Table 10 summarize the polyethylene blends investigated in the literature and are organized according to polymer family. In this section, only the rheological behavior of these blends is presented and discussed; crystallization behavior and property relationships are addressed in subsequent sections.

Despite the limitations discussed above, most studies on the linear viscoelastic behavior of PE blends have used rheological measurements to infer apparent miscibility, compatibility of phase separation. The following discussion is therefore organized around the conclusions drawn from these rheological criteria, while keeping in mind that such conclusions depend strongly on the method of interpretation. Overall, broad trends can be identified, such as the frequent immiscibility of HDPE/LDPE blends in the molten state. However, the literature is far from unanimous, particularly for HDPE/LLDPE and LDPE/LLDPE blends, where molecular weight, molecular weight distribution, branch content and length, comonomer type, and catalyst-related structural differences all influence the observed behavior.

- HDPE/LLDPE

HDPE/LLDPE blends are generally reported to be miscible when the LLDPE has a low number of short-chain branches (typically fewer than 15 branches per 1000 carbon atoms) [11,18,19,31,32,39]. This threshold was predicted by Fan et al. [41] using molecular dynamics simulations. Molar mass also plays a role: blends with low-molecular-weight LLDPE tend to be miscible, while those with high-molecular-weight LLDPE show phase separation [39].

- LDPE/LLDPE

The apparent miscibility of LDPE and LLDPE is particularly controversial, with numerous studies reporting conflicting outcomes. These discrepancies can largely be attributed to variations in short-chain branching (SCB), molecular weight, molecular weigh distribution, comonomer type and catalyst type. Below we attempt to isolate each factor individually; however, since these variables often vary simultaneously, it is difficult to disentangle their individual effect.

#### 2.3.1. Effect of Chain Branching

Hussein et al. [33,34] demonstrated that apparent miscibility between LDPE and Ziegler-Natta LLDPE (ZN-LLDPE) is strongly influenced by the SCB content of the LLDPE. Specifically, ZN-LLDPE with a higher number of methyl branches (e.g., 32.2 CH_3_ groups per 1000 carbon atoms) was more miscible with LDPE than LLDPE with lower branching (14.4 CH_3_/1000C). However, these results were shown to depend on the molecular mass or the polymers. This trend is likely due to closer density and rheological matching between the two components. A higher SCB content reduces the LLDPE’s density, bringing it closer to that of LDPE and potentially enhancing apparent miscibility through improved mechanical compatibility. This suggests that structural parameters such as SCB content may play a dominant role in blend behavior, and that catalyst type should be interpreted through its effect on molecular architecture rather than as an independent parameter.

#### 2.3.2. Effect of Molar Mass

The role of molecular weight in LDPE/LLDPE apparent miscibility is also unclear. Liu et al. [19] and Alamo et al. [42] reported that molecular weight had little to no impact on blend apparent miscibility. In contrast, Hussein et al. observed that lower molecular weight LLDPE blends more readily with LDPE across a broader range of compositions, while higher molecular weight LLDPE showed apparent miscibility only at select concentrations. These inconsistencies suggest that molecular weight may contribute to apparent miscibility primarily through its effect on viscosity ratio and entropic mixing behavior. Interestingly, the comonomer content in LLDPE also appears to influence apparent miscibility. LLDPEs with high hexene content were found to be miscible with LDPE, whereas those with lower hexene content were not [33], reinforcing the importance of branching structure.

#### 2.3.3. Effect of Catalyst Type

Delgadillo et al. [26] reported that LDPE was miscible with metallocene-catalyzed LLDPE (m-LLDPE) but not with ZN-LLDPE, suggesting a possible catalyst effect. However, this conclusion is debated. Indeed different results were obtained by Hussein as shown above. The observed differences may not be due to the catalyst itself, but rather to differences in molecular weight distribution (MWD) associated with each catalyst type. Ziegler-Natta catalysts, depending on the reactor setup, can yield very broad MWDs (polydispersity index, PDI > 6–12) or narrower ones (PDI < 3.5). These variations directly influence the viscosity ratio at the effective shear rate, which in turn affects apparent miscibility. Therefore, resin viscosity—closely tied to MWD—may play a more significant role in apparent miscibility than the catalyst used per se.

- UHMWPE/LLDPE

Chen et al. [27] and Yang et al. [37] studied the blends of ultra-high-molecular-weight polyethylene (UHMWPE) with and LLDPE. Their results showed that UHMWPE is miscible with LDPE but not with LLDPE, highlighting how blend behavior can vary widely depending on structural and compositional factors.

- Compatibilizer effect

Lastly, Lee and Denn [38] evaluated the three binary HDPE/LLDPE, HDPE/LDPE and LLDPE/LDPE. They reported that LLDPE is partially miscible with LDPE in the melt and that HDPE could be used as a compatibilizing agent for LLDPE/LDPE to create a ternary system that appears to be miscible.

More generally, compatibilizers may influence not only interfacial interactions and blend morphology but also crystallization kinetics and crystalline structure, as recently illustrated for other semicrystalline polymer blends [43].

Table 11 summarizes key findings from the literature on how various factors influence the apparent miscibility of different types of polyethylene, as inferred from rheological analysis, although these conclusions should be interpreted with caution due to the variability in experimental conditions and evaluation criteria across studies.

## 3. Crystallization as a Complementary Tool to Interpret PE Blend Apparent Miscibility and Solid State Morphology

### 3.1. Generalities

Polyethylene is a semicrystalline polymer of which the properties can be finely tuned by controlling its degree of crystallinity, a critical factor influencing its mechanical behavior. In its crystalline regions, polyethylene adopts an orthorhombic lattice structure with parameters a_o_ = 0.741, b_o_ = 0.495, and c_o_ = 0.255 nm [5]. However, crystallinity is not an intrinsic, fixed characteristic; rather, it is shaped by the molecular architecture of the polymer chains, particularly the presence and distribution of side branches. These branches are typically excluded from the crystalline phase, remaining in the amorphous regions, thereby reducing overall crystallinity. Moreover, branching influences lamellar thickness, which in turn affects the polymer’s melting temperature [44]. Larbi and Halary [45] demonstrated this effect by comparing high-density polyethylene (HDPE) and low-density polyethylene (LDPE), finding that lamellar thickness varies significantly between the two. In their study, HDPE (Mw = 101, Mn = 16,600 g/mol) exhibited lamellae of approximately 40 nm, whereas LDPE (Mw = 219,000 g/mol, Mn =30,000 g/mol) showed much thinner lamellae, around 20 nm.

When blended in the molten state, two different types of polyethylene can form either a single-phase (monophasic) or multi-phase (heterophasic) system, as discussed earlier in this review. Upon cooling, both components crystallize, but their crystallization pathway depends on the state of mixing in the melt. If the polymers are phase-separated in the melt, crystallization generally occurs within each phase, although the developing crystals may still modify the initial morphology or lead to interfacial crystallization effects. If the blend is apparently homogeneous in the melt, the polymers may either remain mixed during solidification, co-crystallize, or undergo phase separation during cooling. In all cases, crystallization behavior is influenced by several factors, including chain structure, melting temperature, crystallization kinetics of each polymer, blend composition, and cooling or processing conditions. These factors, in turn, determine the morphology of spherulites, lamellar arrangement, and overall crystallinity, all of which directly impact the functional properties of the final material.

Understanding crystallization in polyethylene blends is, therefore, essential because it provides complementary information to melt rheology. While rheological measurements can probe apparent miscibility or phase behavior in the molten state, crystallization analysis reveals how this melt-state organization is translated, modified, or disrupted during solidification. The following section briefly compares the main experimental techniques used to investigate crystalline structure in polyethylene blends, before reviewing the crystallization behavior reported in the literature for different PE blend systems.

### 3.2. Experimental Techniques to Study Crystalline Structures

Numerous studies have investigated the solidification of polyethylene blends using a variety of experimental techniques. These include Differential Scanning Calorimetry (DSC), both isothermal and Successive Self-Annealing (SSA) [10,18,19,20,26,32,33,35,37,40,46,47,48,49,50,51,52,53,54,55]; Small- and Wide-Angle X-ray Scattering (SAXS and WAXS) [18,20,45,49,51]; Neutron Scattering (SANS) [42]; Rheological analyses [56,57,58,59,60,61,62,63], and several microscopy methods such as Polarized Optical Microscopy (POM) [52,53], Scanning Electron Microscopy (SEM) [38], Transmission Electron Microscopy (TEM) [47,52,64,65,66], and Atomic Force Microscopy (AFM) [18,52,54]. Each technique probes a different structural scale and provides different information on crystallinity, lamellar organization, crystal proportions, spherulitic morphology, or crystallization kinetics. These techniques are therefore most powerful when used in combination. Below the most used to study polyethylene blends are reviewed briefly. The purpose of the section is not to provide a detailed methodological review of each technique, but rather to highlight how selected techniques have been used to interpret crystallization, co-crystallization, phase segregation, and solid-state morphology in polyethylene blends. A comparison of the main techniques is provided at the end of the section.

#### 3.2.1. Differential Scanning Calorimetry

Differential scanning calorimetry (DSC) is the most widely used technique to determine melting temperature, crystallization temperature, melting and crystallization enthalpies, and overall degree of crystallinity in polyethylene and polyethylene blends. Although conventional DSC is useful for determining transition temperatures and overall crystallinity, it often lacks the resolution needed to distinguish overlapping crystal populations in complex polyethylene blends.

For this reason, thermal fractionation methods such as step crystallization (SC) and successive self-nucleation and annealing (SSA) have been widely used to characterize polyolefins, particularly LDPE, LLDPE, and recycled polyolefin blends [67,68,69,70]. Both methods rely on the fact that polyethylene chains with different ethylene sequence lengths, branch contents, or lamellar thicknesses crystallize and melt at different temperatures. SC consists of cooling the polymer through a series of crystallization steps, whereas SSA subjects the sample to successive heating and cooling cycles at selected temperatures. Both approaches fractionate the crystalline populations of the material, but SSA generally provides better resolution and shorter experimental times. After SSA or SC treatment, the final heating scan reveals multiple melting endotherms corresponding to different crystal populations. These populations can be related to differences in lamellar thickness, ethylene sequence length, branch distribution, or polymer composition. SSA has, therefore, been used to characterize chain branching in LLDPE and LDPE, determine ethylene sequence length distributions, distinguish different polyethylene fractions, and identify polyolefins in mechanically recycled blends [48,71,72,73,74,75,76,77].

SSA is particularly useful for polyethylene blends because it can help determine whether the different polyethylene fractions crystallize predominantly independently or whether their crystallization behavior is modified by blending. A common approach consists of comparing the final SSA heating curve of the blend with the response expected from the neat components at the same composition. If the blend response closely follows the weighted contribution of the neat components, this may suggest predominantly independent crystallization. In contrast, deviations from this expected response, such as shifted peaks, additional peaks, missing peaks, or changes in peak areas, may indicate co-crystallization or crystallization-induced interactions between chains from different components. However, such interpretations should be made cautiously, because overlapping peaks or limited resolution may mask more subtle co-crystallization phenomena.

**Figure 4:**
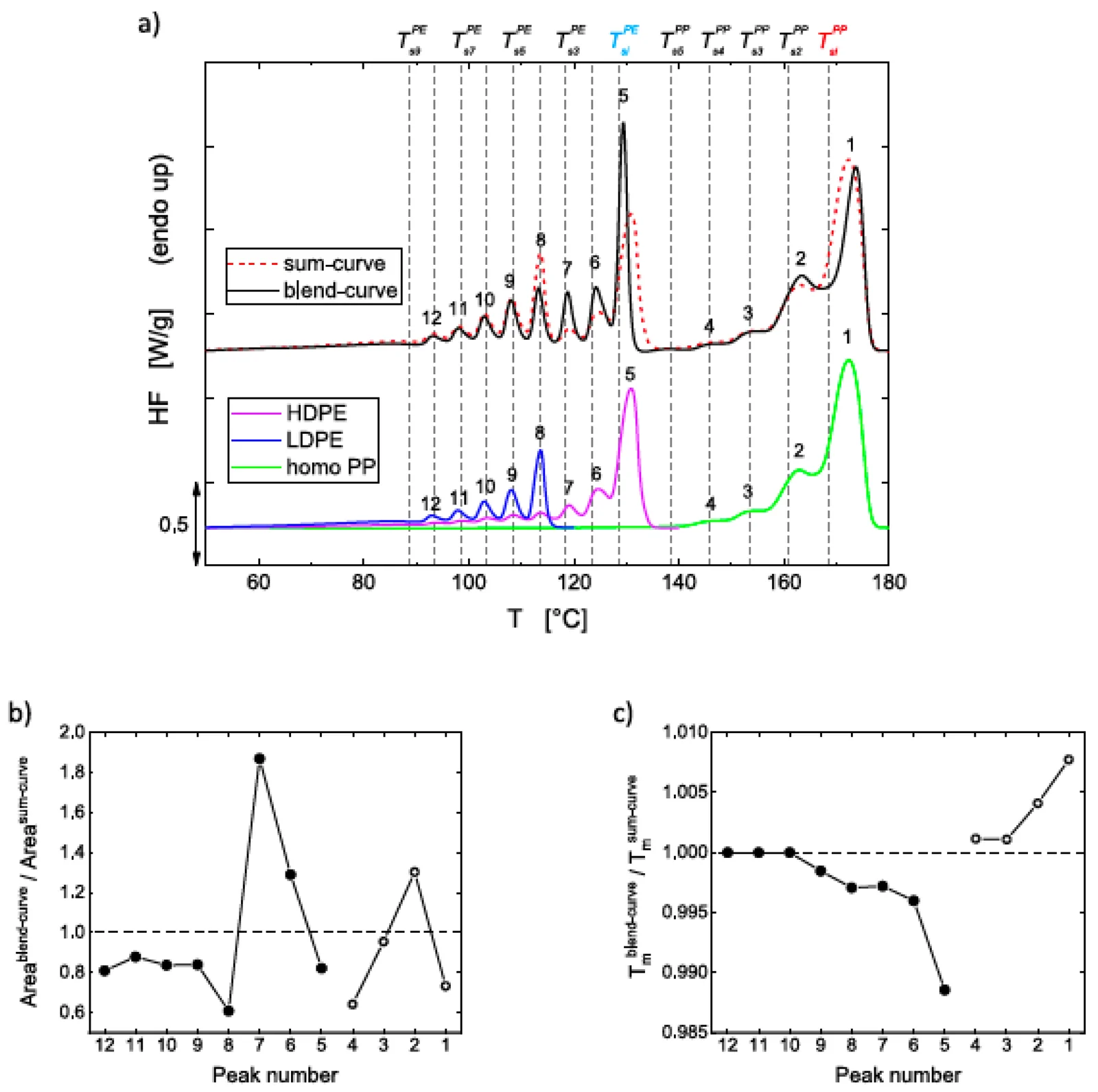
(**a**) Comparison between the last heating curve of a 58.5/20/20 PP/HDPE/LDPE blend subjected to SSA to the sum (scaled by their composition) of the curves of the individual components subjected to the same SSA. (**b**) Ratios of the areas and (**c**) the melting temperatures for the different crystallization peaks. Reprinted with permission from [74] Copyright © 2020 Elsevier Ltd. [74].

This approach is illustrated in Figure 4 for a PP/HDPE/LDPE blend [71]. In this case, some melting peaks followed the expected mixing rule, whereas others deviated from it. The peaks that did not follow the expected response were attributed to co-crystallization between specific LDPE and HDPE crystalline fractions. This example illustrates why SSA can be more informative than conventional DSC for recycled polyolefin blends, where several crystalline populations may overlap.

Overall, DSC provides essential information on transition temperatures and overall crystallinity, whereas SSA provides a more refined analysis of crystal populations and co-crystallization. For polyethylene blends relevant to recycling, SSA is, therefore, a valuable complementary technique to melt rheology because it can reveal whether apparent melt-state miscibility is preserved, lost, or transformed during crystallization.

#### 3.2.2. Rheological Measurements

Flow, whether shear or extension [56,57,58,59,60,61,62,63], can influence the crystallization kinetics and the resulting crystalline morphology, thereby affecting the final functional properties of the material. Rheology is therefore a valuable tool for studying deformation-induced crystallization in polymers. During crystallization, all viscometric properties, including viscosity, storage and loss moduli, increase sharply as the material evolves from a molten state to a partially crystalline or solid–liquid structure. Monitoring the evolution of these rheological properties under controlled deformation and thermal conditions can therefore provide information on the onset of crystallization, crystallization rate and development of solid-like network.

To obtain more direct structural information during crystallization under flow, several authors have coupled rheological measurements with Raman spectroscopy [57,61,63] or small-angle or wide-angle X-ray Scattering [56,58,60,78]. To obtain more direct structural information during crystallization under flow, several authors. These coupled approaches are particularly useful because they connect the macroscopic rheological response to molecular or crystalline structure development. Raman spectroscopy can follow the evolution of crystalline and amorphous bands during cooling or deformation, although distinguishing different polyethylene grades in a blend remains difficult because their chemical structures are very similar. Rheo-SAXS and rheo-WAXS provide complementary information on lamellar organization, crystalline orientation and the development of anisotropic structures under flow.

Yang et al. [58] used rheo-SAXS and rheo-WAXS to investigate the effect of adding a small fraction of high-molecular-weight polyethylene to lower-molecular-weight polyethylene. Blends containing low-molar-mass polyethylene, either MB-50 K or MB-100 K, and high-molar-mass polyethylene, MB-250 K, were subjected to shear flow at 60 s^−1^ for 5 s at 120 °C. The evolution of crystalline structure after shear was then followed by SAXS and WAXS. These measurements made it possible to analyze the development of crystalline planes, lamellar organization and orientation induced by flow. This study illustrates how coupled rheological and scattering measurements can reveal the influence of molecular weight and flow history on polyethylene crystallization.

Overall, rheological measurements during crystallization are particularly relevant for polyethylene blends because they show how processing conditions may alter crystallization pathways, modify solid state morphology and influence the relationship between melt-state behavior and final properties.

#### 3.2.3. Microscopy

Microscopy techniques, including optical microscopy, scanning electron microscopy (SEM), transmission electron microscopy (TEM), and atomic force microscopy (AFM), can provide useful information on the solid state morphology of polyethylene blends [79]. However, the direct observation of phase morphology in blends composed only of polyethylene grades remains challenging. LDPE, LLDPE and HDPE have very similar chemical structures and usually provide limited optical or electron contrast. As a result, distinguishing the different PE-rich phases or crystalline populations often requires specific sample preparation, selective staining, etching, or complementary characterization techniques. Chemical staining or differential etching can sometimes improve contrast, particularly by revealing amorphous or crystalline regions. However, these methods may also modify the morphology being observed and must therefore be interpreted with caution. For PE/PE blends, microscopy is often more useful for visualizing differences in crystalline organization, lamellar morphology, spherulitic structure, or local stiffness contrast than for directly identifying each polyethylene component [80].

AFM is particularly useful because it can provide nanoscale information on surface morphology and phase contrast associated with differences in stiffness or crystalline organization. However, AFM probes relatively small areas, and representative sampling is therefore essential. For this reason, AFM observations should generally be combined with thermal, rheological or scattering measurements before drawing conclusions about blend apparent miscibility or co-crystallization.

**Figure 5:**
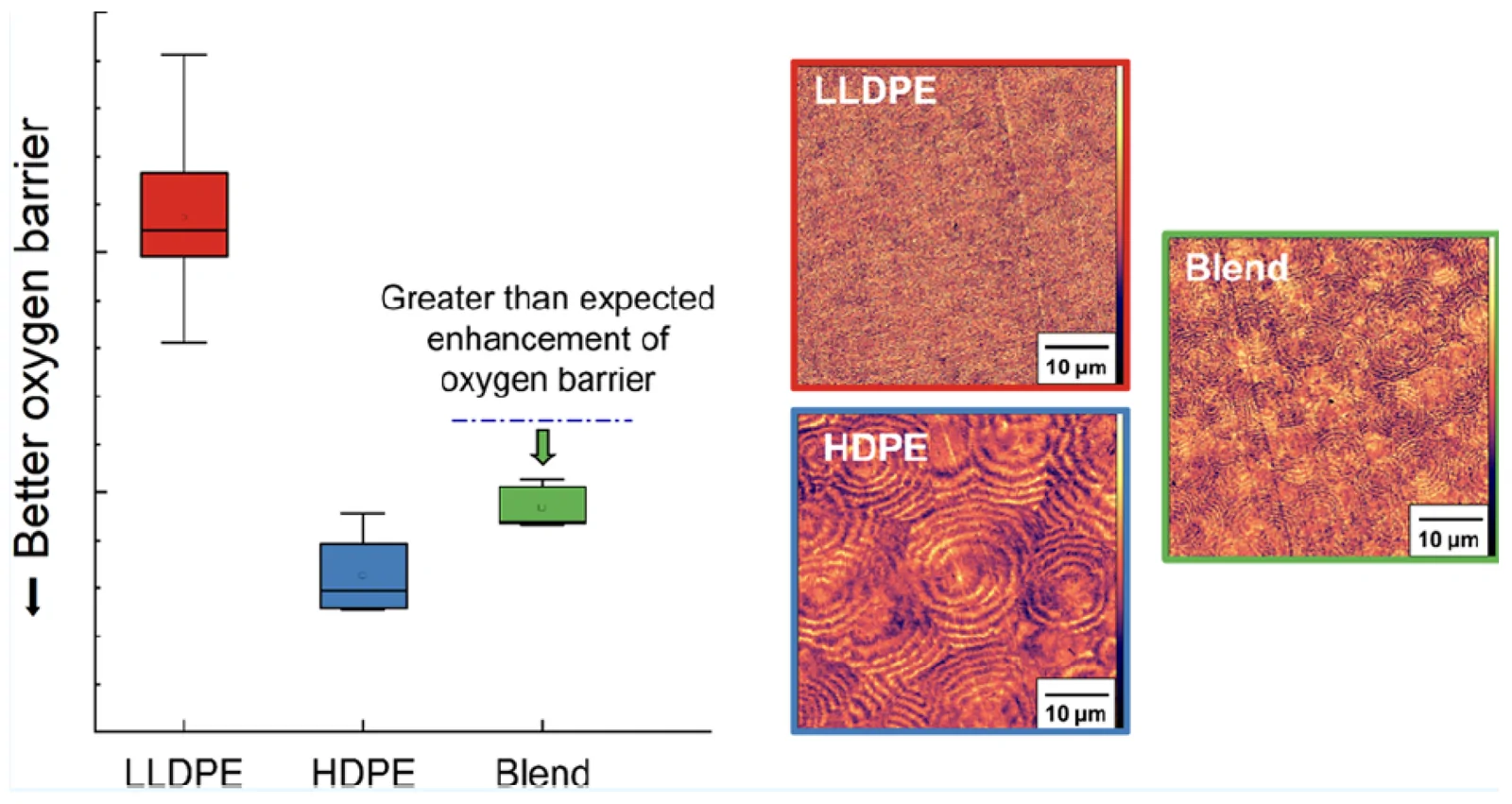
Microstructure of LLDPE/HDPE (80/10) blends by AFM-IR. Reprinted with permission from [57].

A particularly relevant approach is the coupling of AFM with infrared spectroscopy. AFM-IR has been used by Macosko’s group [54] to analyze LLDPE/HDPE blends. In this study, the blends exhibited better oxygen barrier properties than expected from conventional models. The AFM-IR analysis suggested that the addition of HDPE promoted the development of spherulitic structures in the LLDPE-rich phase, creating a more tortuous pathway and reducing oxygen permeability. This example, shown in Figure 5, illustrates how microscopy-based techniques can connect crystalline morphology with functional properties in polyethylene blends.

Overall, microscopy provides valuable local information on crystalline morphology and phase organization, but it rarely provides a complete interpretation on its own. For polyethylene blends, it is most powerful when combined with DSC or SSA, rheology, SAXS/WAXS, or spectroscopic techniques, allowing for solid state morphology to be interpreted in relation to melt-state behavior and crystallization pathways.

#### 3.2.4. Comparison of the Different Techniques

As discussed above, several techniques can be used in a complementary manner to investigate the crystallization and solid state morphology of polyethylene blends. Each technique probes a different structural scale and provides distinct information, such as overall crystallinity, transition temperatures, crystal structure, lamellar organization, spherulitic morphology, or crystallization under flow. Table 12 summarizes the main information provided by each technique and highlights their relevance for the study of polyethylene blend crystallization.

### 3.3. Crystallization of Blends of Polyethylene

#### 3.3.1. Introduction

Table 13, Table 14, Table 15, Table 16, Table 17, Table 18, Table 19 and Table 20 present an overview of studies on the crystallization of blends of polyethylene, as well as the main conclusions reached by the different research groups. Information about the mechanical properties of the blends, when reported in the papers, is also shown in these tables. This information is relevant because the crystalline and phase morphologies developed during cooling, including the degree of crystallinity, lamellar organization, crystal perfection, and interphase structure, ultimately contribute to the mechanical and functional properties of the blends. However, these property data are reported only to provide context, and a detailed analysis of crystalline morphology–property relationships was not undertaken, as this lies outside the main focus of the present review. Earlier studies focused on obtaining the blend through solvent casting, which involves dissolving the polyethylene mixture in xylene and precipitating it in acetone. More recent studies, however, have reported results on blends obtained through melt extrusion.

Although the results are highly diverse and sometimes contradictory, a few general trends can still be identified. Solidification behavior depends not only on the specific polymers involved in the blend, but also on blend composition and processing conditions. In the following section, we attempt to clarify this complex landscape and bring some order to the apparent disorder. To do so, we organize the discussion by blend type: HDPE/LDPE, HDPE/LLDPE, LDPE/LLDPE, blends containing UHMWPE and ternary blends.

#### 3.3.2. Crystallinity in HDPE/LDPE Blends

##### Number of Crystalline Phases in HDPE/LDPE Blends

Conflicting results have been reported regarding the number of crystalline phases formed in HDPE/LDPE blends. Some studies have suggested solid-state apparent miscibility or partial co-crystallization, although the structural characterization was sometimes limited [10]. In contrast, Andersson et al. [49] showed that crystallization behavior strongly depends on cooling rate. In LDPE/HDPE blends containing 5 wt% HDPE, three crystal populations were observed at cooling rates up to 5 °C/min, corresponding to HDPE crystals. This co-crystalline population was not observed at the slowest cooling rate, indicating that thermal history plays a key role in determining whether the components crystallize separately or form mixed crystalline populations.

##### Effect of Blending on Degree of Crystallinity and Lamellar Long Period

Larbi et al. [45] studied how adding LDPE affects the crystalline structure of HDPE using Small-Angle X-ray Scattering (SAXS). Their results showed a monotonic decrease in the degree of crystallinity with increasing LDPE content. Interestingly, the long period of the crystal lamellae exhibited a positive deviation, indicating structural reorganization within the lamellar morphology.

##### Effect of LDPE Structure on Apparent Miscibility and Crystallinity

Branch content also plays a key role in the apparent miscibility and crystallinity of HDPE/LDPE blends. Alamo et al. [42] evaluated blends with HDPE polydispersity ranging from 1 to 6 and LDPE polydispersity from 5 to 17, across a range of branch contents. Using Small-Angle Neutron Scattering (SANS), they found that blends were homogeneous when the LDPE had a low branch content, about 4 branches per 100 carbon atoms for a molecular weight of 105,000 g/mol. However, when the branch content exceeded 8 branches per 100 carbon atoms, phase separation occurred, suggesting that higher branching disrupts apparent miscibility and alters crystallinity.

#### 3.3.3. Crystallinity in HDPE/LLDPE Blends

Numerous studies have been conducted on the HDPE/LLDPE system. While attempts have been made to isolate the effects of blend composition, molecular structure, and thermal history, these factors are often deeply intertwined. For example, phase separation and co-crystallization may both depend on blend composition, comonomer type and cooling rate, making it difficult to assign a given behavior to a single cause. Despite this complexity, some general trends can be observed by grouping the findings under dominant effects.

##### Effect of Composition

The crystallization behavior of HDPE/LLDPE blends depends strongly on blend composition. Higher HDPE contents have generally been associated with a greater tendency toward co-crystallization, whereas lower or intermediate HDPE contents may lead to the formation of two distinct crystal populations [64,66]. However, composition alone is not sufficient to predict the crystallization behavior, since the molecular structure of the LLDPE also plays an important role.

The type of comonomer incorporated in the LLDPE can modify this behavior. For example, blends containing butene- or hexene-based LLDPEs have often exhibited a single melting peak, which may suggest co-crystallization. However, phase separation was reported for one butene-based blend composition, indicating that crystallization behavior is also affected by branch content, branch distribution and crystallizable sequence length, rather than by blend composition alone [10].

##### Effect of LLDPE Structure

The type and amount of short-chain branching in LLDPE strongly influence crystallization behavior of HDPE/LLDPE blends. Tanem and Stori [52] conducted an extensive study on the effect of adding a small fraction of HDPE to LLDPE with varying mol% of butyl branches. When LLDPE contained a comonomer content above approximately 1.8 mol%, the formation of two distinct crystal populations became evident. This result suggested that, although some degree of co-crystallization may have occurred, as indicated by an increase in the crystallization temperature of the LLDPE-rich phase compared to with the neat polymers, increasing comonomer content led to thinner lamellae and disrupted the crystallization of LLDPE in the presence of HDPE [52]. However, this behavior is not universal, since some studies have reported only a limited effect of branching type on the solid state properties of HDPE/LLDPE blends [32].

More detailed structural insights have been gained using Successive Self-Nucleation and Annealing (SSA). These studies suggest that polyethylene blends with similar short-chain branching content and branch distributions are less prone to segregation during SSA and may form more stable co-crystal populations [48,81]. However, since melt-state apparent miscibility was not directly characterized, these conclusions should be interpreted cautiously. In such cases, SSA provides strong evidence of crystallization behavior and thermal fractionation, but does not alone constitute definitive proof of thermodynamic miscibility in the melt.

**Figure 6:**
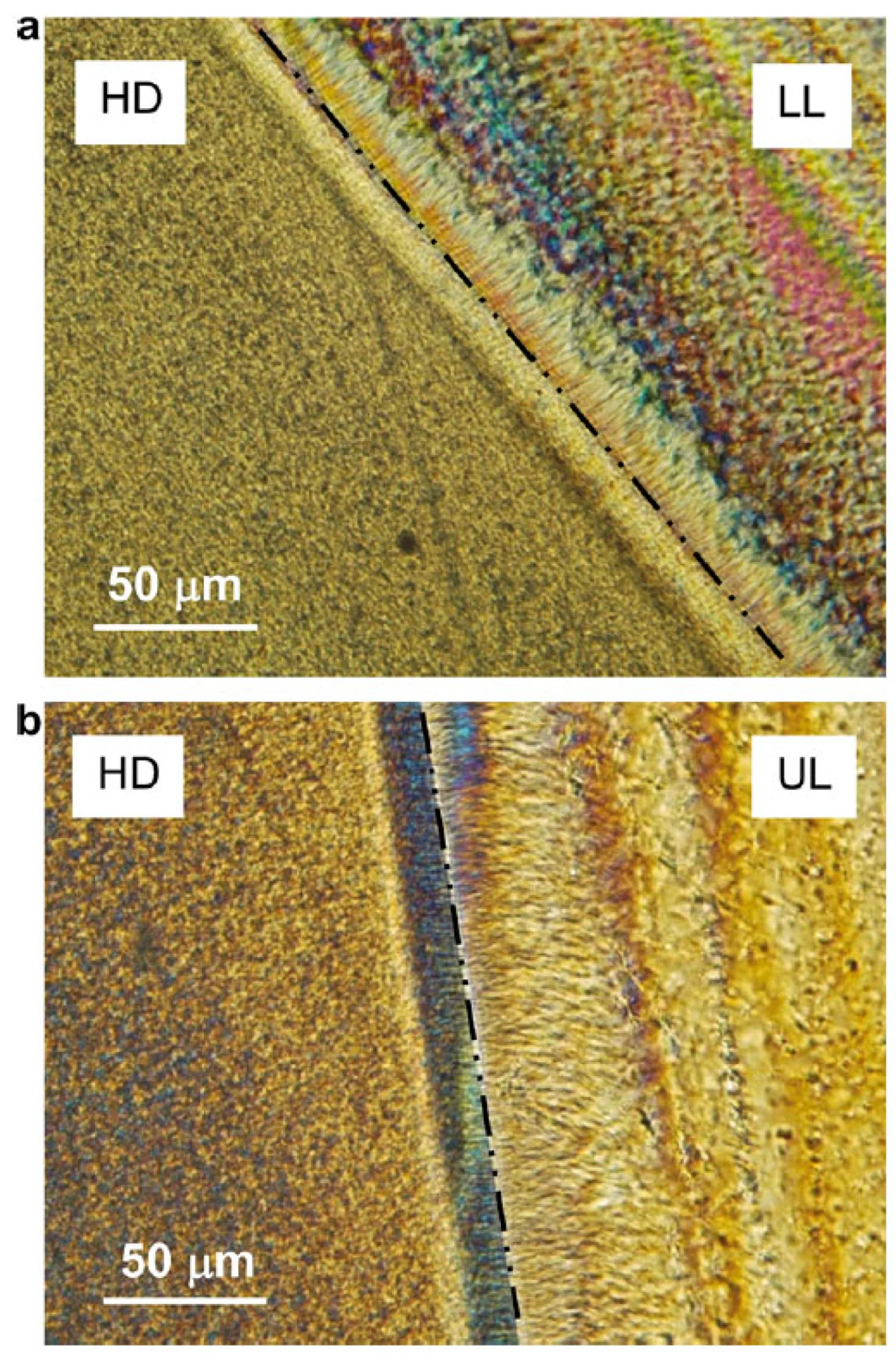
Co-crystallization occurring between HDPE and LLDPE (**a**) or ULDPE (**b**) [18]. Reprinted with permission from [18]. Copyright © 2010 Elsevier Ltd.

Co-crystallization was also reported by Cheng et al. [48], who used SSA to analyze blends of different LLDPE and LDPE grades varying in comonomer type, comonomer content, molar mass, and molar mass distribution. They concluded that for all blends studied (see Table 15), some co-crystallization occurred. However, the extent of co-crystallization varied for each blend. While molar mass and molar mass dispersity did not seem to influence the extent of co-crystallization for blends of LLDPE and LDPE, the co-crystallization of blends of LLDPE depended on the co-chain length. This evidence of co-crystallization was seen by Frederix et al. [18] who demonstrated that it could occur at the interface between immiscible polyethylene as shown Figure 6.

##### Effect of Thermal History

Crystallization behavior is highly sensitive to thermal processing, particularly the cooling rate. When HDPE/LLDPE blends are cooled slowly, they tend to undergo phase separation. In contrast, rapid cooling can suppress segregation and promote the formation of co-crystals [65]. Additionally, the presence of structurally similar components enhances this effect. For instance, when HDPE and VLDPE were combined, the HDPE-rich phase exhibited a melting point depression—a dilution effect—while also nucleating VLDPE crystals. This underscores how thermal history interacts with both blend composition and molecular architecture to dictate the final microstructure [65].

#### 3.3.4. Crystallinity of LDPE/LLDPE Blends

The crystallization behavior of LDPE/LLDPE blends is strongly influenced by branch content and length, as evidenced by the data summarized in Table 15. These structural parameters play a key role in determining the extent of phase separation versus co-crystallization. While the apparent miscibility of LDPE and LLDPE in the molten state varies depending on catalyst type, side chain length and content, and molecular weight. A consistent observation across multiple studies is that some degree of co-crystallization occurs upon cooling, regardless of melt-state apparent miscibility [26,64].

For blends containing metallocene-catalyzed LLDPE (m-LLDPE), partial co-crystallization has been reported in both studies considered. Notably, Xu et al. [35] investigated solvent-cast blends of LDPE and m-LLDPE and concluded that, although the polymers were immiscible in the melt, the observed decrease in melting temperatures for both components suggested that some less-branched LDPE was incorporated into LLDPE, and branches of LLDPE were incorporated into LDPE. This mutual accommodation points to the formation of co-crystals despite melt immiscibility.

In contrast, when LDPE was blended with Ziegler-Natta LLDPE (ZN-LLDPE), the authors identified the presence of a third melting endotherm, which they attributed to the formation of a distinct crystal population. This indicates that the crystallization behavior of LDPE/LLDPE blends depends strongly on the molecular architecture of the LLDPE, including catalyst type, branch distribution and crystallizable sequence length.

#### 3.3.5. Blends Containing UHMWPE

Blends containing UHMWPE have also been investigated, although fewer studies are available [37,46]. Even when UHMWPE is reported to be immiscible with the polyethylene matrix in the molten state, it can still modify crystallization behavior by acting as a nucleating or structure-modifying component. In particular, the presence of UHMWPE has been reported to affect the nucleation and growth of HDPE crystals and to promote the formation or recrystallization of more stable lamellae. These results highlight that melt-state immiscibility does not necessarily imply that one component has no influence on solid-state crystallization.

#### 3.3.6. Ternary Blends

**Figure 7:**
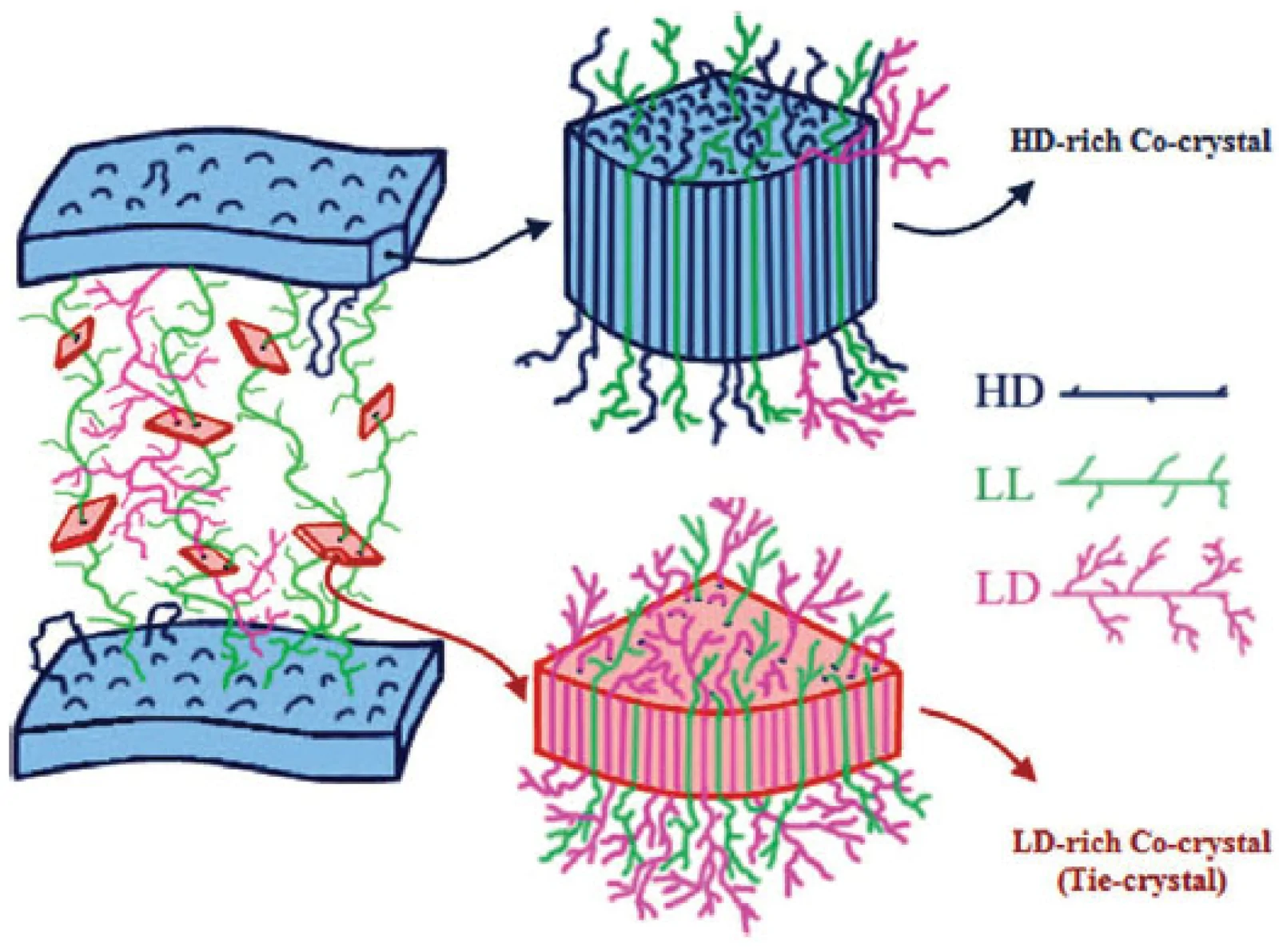
Schematic diagram of the crystalline morphology of HDPE, LLDPE and LDPE blends. Reprinted with permission from [51]. Copyright © 2023 Society of Plastics Engineers [51].

**Figure 8:**
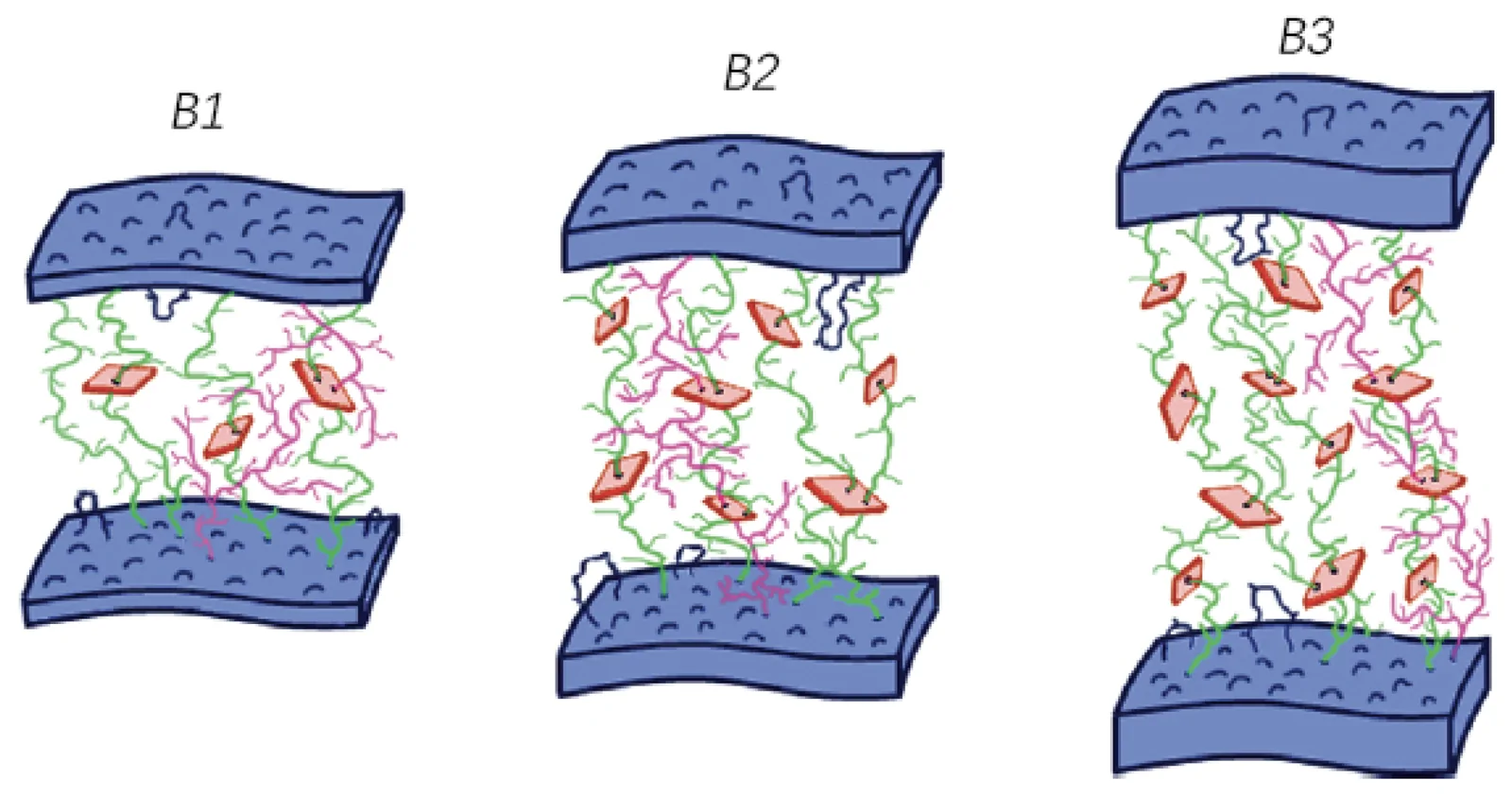
Schematic representation of the ternary blends studied by Khederlou et al. permission from [51]. Copyright © 2023 Society of Plastics Engineers [51].

Ternary blends add another level of complexity because several pairs of polyethylene chains may participate in crystallization. Khederlou et. al. [51] investigated ternary blends of HDPE, LDPE and ZN-LLDPE using rheology, DSC, WAXS, SAXS and field-emission SEM. They concluded that the polymers were miscible in the molten state and that fine LDPE/ZN-LLDPE co-crystals, were dispersed within coarse crystals of HDPE/ZN-LLDPE co-crystals and suggested a model for the crystalline structure (see Figure 7). They observed that increasing the amount of LDPE within the blend would increase the toughness of the blend as measured by tensile tests which they justified by an increase in distances between the HDPE/ZN-LLDPE co-crystals as shown in Figure 8.

They concluded that the polymers were miscible in the molten state and proposed that fine LDPE/ZN-LLDPE co-crystals were dispersed within coarser HDPE/ZN-LLDPE co-crystalline domains, as schematically illustrated in Figure 7. They further reported that increasing the LDPE content improved blend toughness, which they attributed to an increase in the distance between HDPE/ZN-LLDPE co-crystalline domains, as shown in Figure 8. This study illustrates how ternary PE blends can develop complex crystalline morphologies in which co-crystallization and spatial organization of the crystalline domains directly influence mechanical properties.

#### 3.3.7. Conclusion

Although no single study has systematically addressed all influencing parameters that may influence crystallization, including processing conditions, blend composition, comonomer type, chain branching, molar mass, molar mass distribution and catalyst type, several trends can be identified. Solidification behavior depends strongly on both molecular structure and thermal history. Co-crystallization is more likely when polymers share similar chain architecture, branch content and crystallizable sequence lenghts. HDPE can act as a nucleating component and promote crystallization in blends whereas LDPE generally reduces crystallinity and disrupts lamellar organization. In LDPE/LLDPE blends, partial co-crystallization has been reported even when apparent melt-state miscibility is limited, particularly for metallocene-catalyzed LLDPE. Thermal history, especially cooling rate, strongly affects the balance between phase separation independent crystallization and co-crystal formation. UHMWPE, although often reported to be immiscible in the melt, can still modify HDPE crystal morphology. Finally, ternary PE blends may develop complex crystalline structures in which fine LDPE/LLDPE or LDPE/ZN-LLDPE co-crystalline domains influence the spacing between HDPE-rich crystalline regions and contribute to improved mechanical toughness.

Table 21 presents a summary of some key crystallization trends in polyethylene blends.

## 4. Current State of Knowledge on Blends for the Recycling of Post-Consumer Polyethylenes

Several strategies have been reported in the literature to upcycle post-consumer recycled polyethylene (PCR PE), ranging from the incorporation of carbon-based additives [55,82,83,84], lignocellulosic materials [85,86,87] to the use of PCR PE in construction materials [67,68,69,70,88], or in lower-value applications [89,90,91,92,93,94,95]. However, one of the most viable strategies to increase the use of PCR PE while maintaining material performance remains its blending with virgin polymers of the same chemical nature [96,97,98,99,100,101].

For example, Cecon et al. [98] carried out an extensive study on the effect of adding a single-source PCR PE to polyethylene grades of different densities, including LDPE, LLDPE, MDPE, and HDPE. Although different trends were observed depending on the virgin PE grade used, most of the physical properties of the blends followed rule-of-mixtures behavior. However, the results were not interpreted in terms of apparent polymer miscibility, phase morphology, or crystallization behavior, and the detailed molecular nature of the PCR PE was not identified. Similarly, Zeng et al. [102] investigated the blends of recycled HDPE with virgin HDPE and concluded that approximately 75 wt% virgin HDPE was required to obtain properties comparable to those of pure virgin HDPE. They also highlighted the importance of removing contaminants from PCR PE to enable successful reuse.

These studies illustrate both the potential and the limitations of blending as a recycling strategy. In many cases, the lack of fundamental characterization of PCR PE streams limits the universality and transferability of the proposed solutions. Without detailed information on composition, molecular architecture, degradation state, contamination, rheological behavior, and crystallization behavior, it remains difficult to understand the effect of the previous life cycle of the recycled polymer or to predict the performance of the resulting blends. This lack of characterization also hinders the development of standardized formulation strategies for recycled polyethylene.

Two major challenges arise when dealing with PCRPE, first polymer properties may change during service life or repeated processing due to degradation, oxidation, chain scission, branching or crosslinking and the presence of contaminant. Second, separating or identifying the different polyethylene grades present in a post-consumer streams remains difficult because PE grades have similar chemical structure and densities.

Table 22 summarizes the main polyethylene modifications in polyethylene resulting from service-life exposure and reprocessing. During use, PE may be exposed to UV radiation, oxygen, heat, mechanical wear, had contact with food, detergents, oils, or other chemicals. It may also loose stabilizers and other additives, undergo crosslinking, or become contaminated by other polymers, labels, adhesives, inorganic matter, or residual products. Subsequent reprocessing can further modify the molecular structure, as summarized in Table 22.

The first challenge is that the polymers may already have been degraded during their service life. For example, they may have undergone photo-oxidative and thermo-oxidative degradation. A recent review by Gardette and Thérias provides a detailed description of the degradation reactions occurring in polyethylene and of the methods used to evaluate both photo- and thermo-oxidation [103]. These degradations can lead to chain scission, chain branching, change in branching, crosslinking, oxydations and other chemical changes. Mechanical wear may also occur, while antioxidants, carbon black, or compatibilizers may be used to limit degradation or restore some properties [109].

The second challenge, namely the identification and sorting of different PE grades in recycled streams, is equally important. To address this issue, Silva et al. recently developed approaches combining FTIR [112] or Raman spectroscopy [113] with mathematical or chemometric tools to classify polyethylene materials containing different contaminants. Such methods are important because a better identification of the recycled stream can guide the selection of an appropriate virgin polyethylene grade for blending. In another study, Müller and co-workers [114] reported the use of modulated DSC to analyze the composition of model polyolefin blends mimicking PCR polyolefin streams containing HDPE, LLDPE, LDPE and different types of PP. Their approach enabled the quantification of the different polyolefin fractions and was subsequently used to characterize real PCR polyolefin streams.

The effect of contaminants was briefly discussed by Thoden Vqn Velzen et al. [110], which emphasized the importance of obtaining a sufficiently homogeneous PCR PE stream. Kazemi et al. also showed that contamination can modify the response to compatibilization. In their recycled PE/PP blends, the added compatibilizer was less effective than in the corresponding virgin blend. Labels, adhesives, and other packaging residues must therefore also be removed as efficiently as possible.

In addition to degradation during service live, PE may undergo further structural changes during reprocessing. It has long been recognized that repeated processing can lead to chain scission, oxidation, branching or crosslinking, depending on molecular architecture, catalyst type, processing conditions and oxygen exposure [104,105]. However, despite the growing interest in mechanical recycling, relatively few studies have examined in detail the effect of repeated processing on PE structure and properties, especially in blends of recycled and virgin PE [99,102,118].

It was shown in the literature that the effects of repeated processing depend strongly on the initial molecular architecture of the PE, the catalyst system used for its production, and the presence of stabilizers. Moss and Zweifel investigated the degradation and stabilization of different PE grades during multiple extrusion cycles [116]. They showed that unstabilized Phillips HDPE, produced using a chromium-based catalyst, tended to undergo an increase in molecular weight and crosslinking, whereas unstabilized Ziegler HDPE, produced using a titanium-based catalyst, was more prone to chain scission. Phenolic antioxidants and phosphite stabilizers limited degradation, and their combined use provided the greatest stabilization.

Other studies have confirmed that the structural changes induced by reprocessing vary with PE grade. For HDPE, multiple extrusion cycles used to simulate mechanical recycling resulted in a progressive decrease in molecular weight, attributed to oxidative degradation and chain scission during melt extrusion [99]. FTIR analysis also revealed an increase in carbonyl groups, confirming oxidation, while blending the reprocessed HDPE with virgin HDPE partially mitigated some of these effects. In LDPE, repeated extrusion initially promoted chain scission, followed by microgel formation and an increase in melt viscosity after more extensive processing [119]. By contrast, a study of two LLDPE grades subjected to nine extrusion cycles found no clear evidence of crosslinking but identified chain scission and marked changes associated with long-chain branching [117].

The response to reprocessing may become even more complex when the material contains several PE grades, fillers, or additives. Maleki et al. [120], for example, reported that repeated processing reduced the mechanical properties of a ternary blend composed of HDPE, LDPE/LLDPE, and CaCO_3_. This result indicates that the effects of reprocessing cannot be attributed solely to changes in the PE molecular structure, since the presence of fillers and the interactions among the different components may also influence the final properties.

Washing that is performed for recycling and which remove dirt and reduce ash content it can also affect the stabilization state of the recycled material. In one study, washing and subsequent thermal recycling produced only a small change in viscosity but reduced thermo-oxidative stability [115].

All the changes undergone during service life exposure and reprocessing can strongly affect the rheological response of PCR PE/virgin PE blends. Chain scission, branching, crosslinking, and gel formation modify the relaxation spectrum and may change the viscosity ratio between recycled and virgin fractions. Consequently, conclusions drawn from the additivity rule, Cole–Cole plots, time–temperature superposition, Han plots, or van Gurp–Palmen plots may be affected by degradation-induced changes rather than by miscibility alone. For example, additional curvature in a Cole–Cole plot may originate from broadening of the molecular-weight distribution, long-chain branching, or gel formation. Likewise, failure of time–temperature superposition may result from oxidation or structural heterogeneity. If degradation or contamination produces a multiphase blend, the morphology and the characteristic domain size inferred using models such as the Palierne model will also be affected. In this context, the study reported by Dordinejad et al. [117] is particularly relevant because it demonstrated that repeated processing can modify rheological and thermorheological signatures, including Cole–Cole and van Gurp–Palmen plots and the apparent activation energy. Therefore, these plots must be interpreted cautiously when they are used to assess apparent miscibility in PCR PE/virgin PE blends.

Oxidation and contamination may additionally change polarity and interfacial interactions and can therefore modify phase separation and morphology. However, such effects should not automatically be interpreted as changes in thermodynamic miscibility. Rather, they may alter apparent melt homogeneity, interfacial tension, domain stability, and the rheological response. Polymer contaminants and residual additives may similarly transform a nominally single-family PE blend into a more complex multiphase material.

Because these degradation-induced changes can complicate the interpretation of rheological data, recent studies have combined spectroscopic techniques, molecular-weight analysis, and rheological modeling to obtain a more complete understanding of degradation mechanisms in polyethylene [106,107,108]. Spectroscopic and molecular weight analyses can provide evidence of oxidation, chain scission, recombination, or broadening of the molecular weight distribution, while rheological measurements can reveal changes in relaxation behavior, branching, crosslinking, and gel formation. For example, linear viscoelastic data fitted using models such as the Branch-on-Branch model [121] can help distinguish relaxation modes associated with different molecular architectures and may reveal whether reprocessing leads predominantly to chain scission, branching or crosslinking, depending on the PE grade considered. Such complementary analyses are therefore essential for distinguishing degradation-related changes from those associated with apparent miscibility.

These changes are not limited to the melt state and may also strongly affect crystallization behavior. The recycled and virgin fractions no longer necessarily have the same molecular structures, degradation states, or stabilization histories. Chain scission may increase molecular mobility, whereas branching, crosslinking, and oxidation may hinder chain folding or modify crystal perfection. Contaminants, fillers, pigments, and residual additives may also act as nucleating agents. Consequently, separate crystallization, changes in co-crystallization, or shifts in crystallization temperature cannot be attributed only to the original PE grade combination; the degradation and contamination histories must also be considered. For example repeated thermal recycling of LLDPE has been reported to alter crystallinity and branching and to promote crosslinking, consistent with the observed changes in mechanical properties. Under the conditions investigated, the authors nevertheless concluded that two recycling cycles could be performed without a critical loss of performance [102].

Overall, these studies show that efficient use of blending as a recycling strategy requires going beyond simple dilution of PCR PE with virgin PE. It requires a detailed understanding of the composition and degradation state of PCR PE streams, as well as the apparent miscibility, phase morphology and crystallization behavior of the resulting blends. This is precisely where the combined rheological and thermal framework discussed in this review becomes relevant. By linking molecular architecture, melt-state interactions, crystallization behavior and final properties, such an approach can help guide the selection of suitable virgin PE grades and support the formulation of recycled polyethylene blends with more predictable performance. In the next section we will present guidelines for PCRPE/Virgin PE formulations.

## 5. Industrial Implications and Practical Guidelines for PCR PE/Virgin PE Formulation

The industrial use of PCR PE is complicated by the inherent variability of post-consumer feedstocks [122,123,124,125,126,127,128,129]. Unlike virgin PE grades, which are produced with controlled molecular architectures, additive packages, and processing specifications, PCR PE streams may contain several PE grades, other polymers, residual additives, degradation products, and organic or inorganic contaminants. Their composition and properties may also vary among suppliers and production batches. As an example, Fisher et al. [122] investigated the influence of seasonal batch-to-batch fluctuations on post-consumer recycled HDPE. They observed that viscosity could be strongly affected by the possible presence of PP, as well as by residual slip agents such as stearic acid and stearamide or other additives such as PEG. They also emphasized the importance of design for recycling, including the establishment of appropriate guidelines for product design, sorting, and recycling. However, these considerations primarily concern the upstream management of waste streams before the formulation of PCR PE/virgin PE blends. Variability may also arise among producers because a large number of polyethylene grades are available, depending on the polymerization system, catalyst, comonomer type, molecular architecture, and intended application. Consequently, the formulation of PCR PE/virgin PE blends cannot be based solely on the nominal designation or melt flow rate of the recycled material, as this may lead to poorly controlled or inadequate final properties [110].

An effective industrial strategy should, therefore, begin with the characterization of the PCR PE stream, and considerable efforts are currently being made in this direction. For example, Müller and co-workers [114] used modulated DSC to identify and quantify the different polyolefin fractions present in recycled streams. This initial characterization should be followed by the selection and evaluation of suitable virgin PE candidates. The objective is not necessarily to obtain thermodynamic miscibility, but to develop a blend with adequate processing stability, controlled phase and crystalline morphologies, and properties suitable for the targeted application. The rheological and thermal methods discussed throughout this review provide complementary information for making these decisions.

Table 23 summarizes this approach as a practical sequence of questions, experimental methods, and informative outcomes. The proposed framework begins with the identification of degradation and contamination, continues with the assessment of melt state and solidification behavior, and ends with the validation of application-specific properties. Because PCR PE composition and degradation state may vary between batches, this sequence should be viewed as a qualification and formulation framework rather than as a universal formulation recipe.

## 6. Knowledge Gaps and Future Perspectives

Despite the large number of studies devoted to PE blends, several important knowledge gaps remain before the behavior of PCR PE/virgin PE systems can be predicted reliably. A major difficulty is that recycled polyethylene is characterized not only by its original molecular architecture, but also by its previous service life and processing history. The composition, molecular architecture, degradation state, additive content, and contamination level of PCR PE streams are often insufficiently known. This makes it difficult to distinguish the effects of thermodynamic incompatibility from those arising from differences in molar mass, branching, oxidation, contamination, or previous processing.

The level of uncertainty depends strongly on the origin of the recycled material. Post-industrial PE is generally easier to characterize because the original grade, formulation, and processing history are often known. In this case, rheological characterization, combined with spectroscopic, molecular weight, and thermal analyses, can be used to determine how processing has modified the polymer structure. Post-consumer PE is considerably more complex because it may contain several PE grades, other polymers, residual additives, degradation products, and organic or inorganic contaminants.

The effects of service life should therefore be investigated more systematically. PE packaging may have been exposed to ultraviolet radiation, heat, oxygen, detergents, oils, food products, cosmetics, or household chemicals. Contact with products such as tomato-based foods, shampoos, cleaning agents, or oils may affect extraction, additive migration, oxidation, swelling, or surface contamination. These effects are unlikely to be identical for all PE grades because commercial polyethylene materials differ in catalyst system, comonomer type, branching distribution, molar mass distribution, and stabilization package. Considering the very large number of available PE grades, systematic studies would be required to establish how representative service-life environments affect the molecular structure, rheological behavior, crystallization, and recyclability of each type of PE.

Such studies could eventually support the development of large and standardized databases relating the original PE grade and formulation to its service-life exposure, degradation state, molecular architecture, rheological response, crystallization behavior, morphology, and final properties. As a first step, data-driven and machine-learning approaches could help identify the different polymers present in recycled streams. For example, machine learning has been combined with DSC data to quantify the relative amounts of PE and PP in polyolefin blends [130]. These approaches could then be extended to predict how a given PE grade is likely to evolve under specific exposure conditions and to assist in selecting a suitable virgin PE grade, additive package, stabilizer, or compatibilization strategy for a given recycled stream. However, the reliability of such tools would depend on the availability of sufficiently large, consistent, and well-characterized datasets integrating rheological, thermal spectroscopic, molecular morphological and property data. At present, the amount of data required to account for the diversity of PE grades, applications, exposure conditions, contaminants, and processing histories remains a major limitation.

Improved traceability could partly reduce this uncertainty. Machine-readable identifiers, barcodes, QR codes, digital watermarks, or digital product passports could provide information on the original PE grade, additive package, intended application, and recommended recycling route. If the identity of the polymer were known at the sorting stage, the main remaining uncertainty would concern the extent to which it had been modified during service life and reprocessing. This would make it easier to separate the problem of material identification from that of degradation. Traceability information could then be combined with analytical measurements and predictive tools to guide sorting and formulation. However, such systems would need to be designed so that the labels, inks, adhesives, or markers used for identification do not themselves become contaminants.

Several studies have already explored this type of approach. For example, Rumetshofer et al. used a digital product passport to ensure the traceability of a bottle-cap waste stream throughout its recycling and transformation into a new product [131]. Another study discussed the use of digital product passports to improve the traceability and quality management of plastic waste and to facilitate its circulation within a circular economy framework [132]. Such approaches are highly promising, although their implementation would require action upstream, including the redesign of products and packaging so that relevant material information can accompany them throughout their life cycle. This would represent a substantial change in current practice, but it could greatly facilitate future sorting, qualification, and recycling strategies.

Once the composition, identity, and degradation state of a PCR PE stream are known, the next challenge is to determine which virgin PE grade should be selected for blending. This requires systematic rheological and crystallization studies because the behavior of the blend depends not only on the original structures of the two components, but also on how the recycled fraction has been altered during use and processing. Processing conditions, including temperature, residence time, shear history, oxygen exposure, blend composition, and cooling rate, must also be considered because they may further modify molecular architecture, morphology, and crystallization during blend preparation.

Machine-learning methods could ultimately assist in this formulation step by combining information on composition, degradation, molecular architecture, rheological behavior, crystallization, morphology, and targeted properties. Such models could potentially recommend whether a PCR PE stream should be diluted with a specific virgin PE grade, restabilized, compatibilized, reactively modified, or directed toward another application. Developing such tools would first require the generation of large amounts of high-quality rheological, DSC, and SSA data to train and validate the predictive models.

Some initial efforts have already been reported. For example, Liang et al. and Bishnu et al. used neural-network-based approaches to classify the apparent miscibility or predict the morphology of polymer-blend systems [133,134]. However, these studies remain exploratory. The number of variables governing polymer blend behavior is extremely large, including molecular architecture, molar mass distribution, composition, processing history, degradation state, and experimental conditions. Considerable experimental effort will therefore be required to generate sufficiently large, standardized, and representative datasets before reliable AI-assisted formulation tools can be developed. Nevertheless, this approach represents a promising direction for the future.

Advanced compatibilization and reactive-extrusion strategies may also be particularly useful when PCR PE streams contain immiscible polymer contaminants or form unstable multiphase morphologies. However, these approaches should be selected according to the actual composition, morphology, and interfacial behavior of the recycled stream rather than applied systematically.

Further progress is also required in rheological characterization and modeling. The criteria currently used to assess apparent melt miscibility, including the additivity rule, Cole–Cole plots, time–temperature superposition, Han plots, and van Gurp–Palmen plots, are often considered separately and may provide ambiguous or contradictory conclusions. Future work should develop integrated interpretation frameworks in which several rheological criteria are applied to the same materials and validated against molecular-weight, spectroscopic, thermal, and morphological observations. This would help distinguish changes caused by apparent miscibility from those resulting from degradation, molecular weight broadening, long-chain branching, crosslinking, gel formation, or contamination.

Additional theoretical developments in molecular rheology are also needed. Architecture-based models, such as the Branch-on-Branch model [121] and related tube-based or constitutive approaches [135], may help determine how processing and service-life exposure modify chain architecture and relaxation behavior. Such models could potentially distinguish whether degradation leads predominantly to chain scission, branching, crosslinking, or changes in the distribution of relaxation modes. However, most existing models were developed for relatively well-defined polymers and must be further adapted and validated for heterogeneous, contaminated, and partially degraded PCR PE streams, for which the initial molecular structure is often poorly known.

Greater attention should also be given to validating rheological criteria for apparent miscibility against direct structural and thermal observations. Future studies should combine molecular, spectroscopic, rheological, thermal, and morphological characterization on the same materials and under the same processing histories. In-line rheological and spectroscopic monitoring may also provide useful tools for detecting batch-to-batch variability, degradation, oxidation, viscosity changes, and processing instability during recycling. These methods could support process optimization and quality control, provided that sufficiently robust relationships are established between in-line signals and the molecular and morphological state of the material.

Beyond the selection of a suitable virgin PE grade, future research should also explore new routes for increasing the value of PCR PE streams that cannot be readily reformulated through simple blending. This may include the development of more effective or more selective compatibilizers, particularly for streams containing immiscible polymer contaminants, as well as reactive-extrusion strategies tailored to the actual composition and interfacial characteristics of the recycled material. Another promising route is the incorporation of functional fillers, such as electrically conductive carbon-based particles, to create recycled composites for higher-value applications requiring electrical conductivity, antistatic behavior, electromagnetic shielding, sensing, or other functional properties. In such systems, rheology remains essential for controlling filler dispersion, phase morphology, processing behavior, and the formation and stability of conductive networks.

Overall, future research should move away from isolated measurements and toward integrated composition–service-life exposure–degradation–processing–rheology–crystallization–morphology–property datasets. Such an approach would support the qualification of PCR PE streams, the rational selection of virgin PE grades, and the development of recycled polyethylene blends with more predictable processing and performance.

## 7. Conclusions

Plastic waste management remains a major environmental and technological challenge, and increasing the use of post-consumer recycled polyethylene (PCR PE) requires strategies that go beyond empirical formulation. Blending PCR PE with virgin polyethylene of similar chemical nature is one of the most practical approaches to improve processability and final properties while reducing the consumption of virgin polymer. However, the success of this strategy depends on a detailed understanding of the recycled stream, including its composition, degradation state, contamination level, molecular architecture, and crystallization behavior.

This review has shown that the interpretation of polyethylene blend behavior is complex because PE grades cannot be treated simply as HDPE, LDPE, or LLDPE labels. Molecular weight, molar mass distribution, short- and long-chain branching, comonomer type, catalyst type, and processing history all influence melt state behavior and solidification. As a result, the literature reports sometimes contradictory conclusions regarding apparent miscibility, compatibility, phase separation, and co-crystallization in polyethylene blends.

Linear viscoelastic rheology provides a valuable framework to infer apparent melt miscibility, compatibility, and phase behavior. Criteria such as the additivity rule, Cole-Cole plots, Han plots, time-temperature superposition, and van Gurp–Palmen plots can reveal important differences in relaxation behavior. However, these approaches should not be considered definitive proof of thermodynamic miscibility. They must be interpreted cautiously and, whenever possible, combined with additional structural and thermal characterization. When blends are immiscible, models such as those of Palierne, Bousmina, or YZZ can provide further insight into morphology and interfacial effects.

Crystallization analysis provides essential complementary information because the structure developed in the melt may be preserved, modified, or even contradicted during cooling. The polyethylene forming the blend may crystallize independently, phase-segregate during cooling, or form co-crystalline populations depending on molecular architecture, composition, cooling rate, and flow history. Techniques such as DSC, SSA, SAXS/WAXS, microscopy, and rheological measurements during crystallization are, therefore, important for understanding how melt state interactions translate into solid-state morphology and final properties.

Future work should focus on the standardized and more complete characterization of PCR PE streams, including molecular, rheological, thermal, and morphological descriptors. Advanced techniques such as AFM-IR, rheology coupled with Raman spectroscopy, and rheo-SAXS/WAXS may provide deeper insight into how polyethylene blends crystallize under processing-relevant conditions. Such information will be increasingly important for developing data-driven and physically informed strategies for the upcycling of recycled polyethylene.

## Figures and Tables

**Table 1:** Definitions of miscibility, apparent miscibility and compatibility.

Terminology	Definition
Miscible	A polymer blend that is mixed at the molecular level and forms a single homogeneous phase. Thermodynamically, miscibility is associated with a negative Gibbs free energy of mixing under the temperature, pressure, and composition conditions considered.
Apparently miscible	A polymer blend for which rheological measurements do not allow for the relaxation processes of the individual components to be distinguished. In such cases, the components appear to relax together. However, this observation does not necessarily indicate molecular-level miscibility and should not be regarded as proof of thermodynamic miscibility.
Compatible	A thermodynamically immiscible polymer blend that exhibits a relatively stable multiphase morphology and satisfactory processing or end-use properties. Compatibility may be intrinsic or may be improved through the addition of a compatibilizer or by promoting specific interactions between the phases.

**Table 2:** Practical guide to rheological methods used to assess apparent melt miscibility and blend morphology.

Method	How to Use the Method	Principle and Possible Conclusions	Main Advantages	Limitations	Recommended Experimental Conditions and Practical Considerations	Ref.
**Additivity rule (AR)**	A selected rheological quantity, such as complex viscosity, G′, G″, or a characteristic relaxation parameter, is plotted as a function of blend composition and compared with a linear or logarithmic mixing rule calculated from the neat components.	A deviation from the selected mixing rule indicates non-ideal rheological behavior and may reveal interactions, morphological effects, or distinct relaxation contributions. A positive deviation behavior is commonly associated to immiscibility whereas a negative deviation behavior is associated to miscibility.	Simple, rapid, and useful as an initial screening method. It requires only data for the neat components and blends.	Entirely empirical. The result depends strongly on the rheological quantity selected, the frequency at which the rheological data is selected and temperature. Deviations may arise from molecular architecture, phase morphology, or interfacial effects and are not unique evidence of miscibility or immiscibility.	Use data obtained at the same temperature, frequency, strain amplitude, and thermal history. Cover a sufficiently broad composition range. Use only as a preliminary test and combine with TTS, Han or VGP plots, thermal analysis, and morphological observations.	[11,16,17,18,20,31,32,33,34,35,36]
**Cole–Cole plot (CCP)**	The loss modulus G″ is plotted against the storage modulus G′ while frequency is varied; frequency is therefore implicit in the plot. Depending on the convention used in the literature, analogous Cole–Cole representations may also be constructed from the real and imaginary components of the complex viscosity.	A smooth single arc, or a blend curve lying between those of the neat components, may be consistent with a common or strongly overlapping relaxation spectrum and considered as the result of apparent miscibility. Multiple arcs, shoulders, or systematic departures from the component curves can indicate distinct relaxation processes or phase heterogeneity.	Provides a compact visual representation of the relaxation response without an explicit frequency axis and is less dependent on a single rheological value than the additivity rule.	An intermediate blend curve is not, by itself, proof of thermodynamic miscibility The observations support only apparent melt miscibility and do not prove molecular-level miscibility. Frequency information is lost. The plot is temperature dependent and may be difficult to interpret for broad molecular weight distributions, long-chain branching, or several overlapping relaxation modes.	Obtain all data in the linear viscoelastic regime at the same temperature and over a broad frequency range. Compare each blend with both neat components and use the method together with TTS, Han or VGP analyses and independent thermal or morphological characterization.	[10,11,17,20,32,36]
**Time–temperature superposition (TTS)**	G′, G″, complex viscosity, or related linear viscoelastic data measured at several temperatures are shifted horizontally to construct a master curve at a reference temperature. Shift factors may be described using the Williams-Landel-Ferry equation near the glass-transition region or an Arrhenius-type temperature dependence when appropriate.	Successful superposition indicates that the overall linear viscoelastic behavior of the material is thermorheologically simple over the experimental window (temperature and frequencies) considered. This indicates that the relaxation processes to the response have similar temperature dependences. For a blend, thermorheological simplicity may support apparent melt homogeneity or apparent miscibility, but it does not demonstrate thermodynamic miscibility because immiscible components can have similar activation energies.	Extends the accessible frequency or time window and directly tests the temperature dependence of the relaxation spectrum. It is more informative than a single-temperature graphical criterion.	Requires measurements at several temperatures and sufficient overlap between adjacent frequency sweeps. Degradation, crystallization, phase separation, or morphology evolution can invalidate shifting. Successful superposition can also occur in an immiscible blend when the components have similar temperature dependences, whereas failure may arise from branching or a broad relaxation spectrum rather than phase separation.	Work within the linear viscoelastic regime and verify thermal stability and equilibration at every temperature. Avoid crystallization and degradation. Use the same reference temperature and confirm that the same shift factors satisfactorily superpose both G′ and G″. Report the shift-factor dependence and any vertical shifts used.	[26,33]
**Han plot (HP)**	Log G′ is plotted against log G″ using data obtained over a frequency sweep. To test thermorheological simplicity, Han plots obtained at several temperatures for the same material and blend composition are compared on the same graph; frequency is implicit.	Superposition of the curves obtained at different temperatures for a given composition indicates thermorheological simplicity. Temperature-dependent displacement or changes in slope indicate thermorheological complexity and may reveal distinct relaxation processes. Curve collapse may support apparent melt miscibility, but it does not establish thermodynamic miscibility.	Provides a straightforward graphical test of thermorheological simplicity without first calculating horizontal shift factors. It can highlight changes in the shape of the relaxation spectrum.	Frequency information is removed, and the interpretation remains qualitative. Immiscible components with similar temperature dependences may still produce superposed curves. Importantly, comparing different blend compositions at only one temperature is not equivalent to the Han temperature-invariance criterion and should not be used alone to infer miscibility.	For each composition, acquire frequency sweeps at several temperatures in the linear viscoelastic regime and with the same thermal history. Use a sufficiently broad frequency range and compare the results with those of the neat components. Confirm conclusions using TTS, VGP, thermal, and morphological analyses.	[10,11,20,36,37]
**Van Gurp–Palmen plot (VGP)**	The phase plotted against the magnitude of the complex modulus, |G*|, generally on a logarithmic modulus scale, using measurements obtained at several temperatures. To test thermorheological simplicity, van Gurp plots obtained at several temperatures for the same material and blend composition are compared on the same graph; frequency is implicit.	Superposition of the isothermal curves indicates thermorheological simplicity, whereas lack of superposition reveals relaxation mechanisms with different temperature dependences. In blends, this can identify thermorheological complexity associated with distinct phases or interfacial structures, but curve superposition supports only apparent miscibility and not thermodynamic miscibility.	Provides a straightforward graphical test of thermorheological simplicity without first calculating horizontal shift factors. It can highlight changes in the shape of the relaxation spectrum.	The interpretation is not unique: immiscible components with similar temperature dependences may superpose, while non-superposition can also result from long-chain branching, polydispersity, degradation, or a network structure. Data at very low or very high moduli may be affected by experimental uncertainty.	Same as for HP	[11,26]
**Palierne and Bousmina emulsion models (PB)**	The linear viscoelastic response of the blend is measured and compared with that predicted by the Palierne constitutive equation. This model relates the complex modulus of an immiscible matrix–droplet blend to the complex moduli of the matrix and dispersed phase, the dispersed-phase volume fraction, droplet size or size distribution, and interfacial tension. By fitting the model to the measured blend response, the interfacial tension can be estimated when the droplet size is known from microscopy, or the droplet size can be estimated when the interfacial tension is known independently.	Agreement between the experimental and modeled moduli supports an approximately spherical dispersed-droplet morphology and permits quantification of the interfacial relaxation contribution. The fitted parameter can be used to relate linear rheology to droplet size and interfacial tension. Repeated measurements can be used to follow coarsening, refinement, or stabilization of the co-continuous morphology during annealing or deformation.	Provides a quantitative connection between rheology, morphology, and interfacial properties. It can estimate an otherwise difficult-to-measure interfacial tension or characteristic droplet size. It can also follow structural evolution without relying solely on ex-situ microscopy.	Assumes an immiscible matrix-droplet morphology, small-amplitude deformation, and sufficiently spherical droplets. Accuracy decreases for broad size distributions, irregular or co-continuous morphologies, strong coalescence during the test, interfacial viscoelasticity not included in the selected model, or highly correlated fitting parameters. Very small droplets may relax below the experimentally accessible frequency range.	Verify the dispersed-droplet morphology independently. Measure the neat-component moduli at the same temperature and within the linear viscoelastic regime and with materials having undergone the same thermomechanical treatment. Use reliable phase volume fractions and either independent microscopy or an independent interfacial-tension value. Include sufficiently low frequencies and ensure that morphology does not evolve significantly during the test. For evolving systems, use controlled time sweeps or annealing protocols and distinguish time-dependent morphology changes from frequency dependence.	[26,31,33,34,38]
**Yu–Zhou–Zhou model (YZZ)**	The linear viscoelastic response of the co-continuous blend is measured and compared with that predicted by the Yu–Zhou–Zhou constitutive equation. This model represents the complex modulus of an immiscible blend as the sum of the contributions of the two neat components and an additional interfacial contribution. By fitting the model to the measured blend response, a characteristic domain size can be estimated when the component moduli, blend composition, and interfacial tension are known.	A satisfactory fit supports the use of a co-continuous rheological description and enables the characteristic domain size to be estimated. Repeated measurements can be used to follow coarsening, refinement, or stabilization of the co-continuous morphology during annealing or deformation.	Provides a quantitative rheology-morphology relationship specifically for co-continuous blends and can follow structural evolution without relying solely on ex situ microscopy.	Requires a genuinely co-continuous morphology and reliable values for the neat-component moduli, blend composition, and interfacial tension.	Confirm co-continuity independently by microscopy, image analysis, or selective extraction. Use linear viscoelastic measurements at the same temperature as the component data and a known or independently estimated interfacial tension. For evolving systems, use controlled time sweeps or annealing protocols and distinguish time-dependent morphology changes from frequency dependence.	[15,29,30]

Abbreviations: G′, storage modulus; G″, loss modulus; G*, complex modulus; delta, phase angle; TTS, time–temperature superposition.

**Table 3:** Rheological characterization of HDPE/LDPE blends.

HDPE/LDPE										
Study Number	Material Information			Processing Conditions		Rheological Characterization		Results and Discussion		Ref.
	Info.	Polymer 1 HDPE	Polymer 2 LDPE					Rheological Observation	Authors Conclusion	
S9	ρ	0.961	0.923	Pr.	Haake Polydrive melt blender	AR	A	AR: Follows the log-additivity rules CC P.: semicircular shape + all the blends compositions lie between pure resins Steady Shear: N1 according to the composition is almost linear	Miscible	[17]
	$\bar{M_{w}}$	116,000	100,000			TTS	NA			
	$\bar{M_{n}}$	NA	NA	T	NA	Cross.	NA			
	PDI	6.5	4.1			CC P.	A			
	Cat.	NA	NA							
	Com.	X	X	RPM	NA	Han P.	NA			
	SCB	0	9 SCB/1000C			VGP	NA			
	LCB	0	3 SCB/1000C	RT	NA	PM	NA			
	Add.	50/50 mixture of Irganox I1010 and Irgafox 168				Ext. Visco	NA			
	MFI	1.2	1.2	Comp.	10/90; 30/70;50/50; 70/30; 90/10	Others	Steady shear measurement			
	$\eta_{0}\;(T)$	NA	NA							
S9	ρ	0.961	0.921	Pr.	Haake Polydrive melt blender	AR	A	AR: positive deviation CC P.: no semicircular shape for all the blends compositions + all the blends compositions were higher than the pure resins Steady Shear: N1 according to the composition showed a positive deviation	Immiscible	[17]
	$\bar{M_{w}}$	116,000	100,000			TTS	NA			
	$\bar{M_{n}}$	NA	NA	T	NA	Cross.	NA			
	PDI	6.5	4.1			CC P.	A			
	Cat.	NA	NA							
	Com.	X	X	RPM	NA	Han P.	NA			
	SCB	0	9 SCB/1000C			VGP	NA			
	LCB	0	3 SCB/1000C	RT	NA	PM	NA			
	Add.	50/50 mixture of Irganox I1010 and Irgafox 168				Ext. Visco	NA			
	MFI	1.2	1.2	Comp.	10/90; 30/70;50/50; 70/30; 90/10	Others	Steady shear measurement			
	$\eta_{0}\;(T)$	NA	NA							
S2	ρ	0.937	0.921	Pr.	Single screw extruder	AR	**A**	AR: negative deviation for 20/80 and 60/40 (HDPE-LDPE) Han Plot: Blend resin are compatible with pure resin + phase separation at 80/20 CC P.: no superposition	Immiscible	[10]
	$\bar{M_{w}}$	NA	NA			TTS	NA			
	$\bar{M_{n}}$	NA	NA	T	190; 200; 220	Cross.	NA			
	PDI	NA	NA			CC P.	**A**			
	Cat.	NA	NA							
	Com.	Hexene	NA	RPM	80	Han P.	**A**			
	SCB	NA	NA			VGP	NA			
	LCB	X	NA	RT	NA	PM	NA			
	Add.	NA	NA			Ext. Visco	NA			
	MFI	0.28	0.75	Comp.	20/80; 40/60; 60/40; 80/20	Others	NA			
	$\eta_{0}\;(T)$	NA	NA							
S3	ρ	NA	NA	Pr.	PRISM 16-mm co-rotating twin screw extruder	AR	A	Double reptation model: Good fit	Miscible	[38]
	$\bar{M_{w}}$	40,000	388,400			TTS	NA			
	$\bar{M_{n}}$	NA	NA	T	160 °C in barrel zone and 155 °C for the die	Cross.	NA			
	PDI	3	10.0			CC P.	NA			
	Cat.	NA	NA							
	Com.	NA	NA	RPM	50	Han P.	NA			
	SCB	X	X			VGP	NA			
	LCB	X	3.2 CB/100C	RT	NA	PM	A			
	Add.	NA	NA			Ext. Visco	NA			
	MFI	NA	NA	Comp.	10/90; 20/80; 40/60; 60/40; 80/20; 90/10	Others	Double reptation model of Tsenoglou and des cloiseaux (92–94)			
	$\eta_{0}\;(T)$	448 at 160 °C	32.60 at 160 °C							
S7	ρ	NA	NA	Pr.	Thermo-Scientific HAAKE Polylab QC Rheomix	AR	A	AR: follows the log additivity rules CC P.: semicircular shape Han P.: Absence of superposition VGP: Absence of superposition + δ at the lowest G* decrease with the increase in the LDPE content	Immiscible	[11]
	$\bar{M_{w}}$	NA	NA			TTS	NA			
	$\bar{M_{n}}$	NA	NA	T	180	Cross.	A			
	PDI	NA	NA			CC P.	A			
	Cat.	NA	NA							
	Com.	NA	NA	RPM	60	Han P.	A			
	SCB	NA	NA			VGP	A			
	LCB	NA	NA	RT	5	PM	NA			
	Add.	NA	NA			Ext. Visco	NA			
	MFI	4.5	2.7	Comp.	10/90; 30/70; 50/50; 70/30; 90/10	Others	Relaxation Spectrum			
	$\eta_{0}\;(T)$	NA	NA							
S10	ρ	0.952	0.922	Pr.	Haake Rheocord-900	AR	A	AR: For HDPE-rich blends positive deviation, while for LDPE-rich blends follow the log-additivity rules CC. P.: For LDPE-rich blends, semicircular shape is obtained. Whereas for HDPE-rich blends, absence of semi-circulars hape	Partial miscibility according to the composition: Miscible for LDPE-rich blends Immiscible for HDPE-rich blend	[20]
	$\bar{M_{w}}$	NA	NA			TTS	NA			
	$\bar{M_{n}}$	NA	NA	T	230	Cross.	NA			
	PDI	NA	NA			CC P.	A			
	Cat.	NA	NA							
	Com.	NA	NA	RPM	NA	Han P.	NA			
	SCB	NA	NA			VGP	NA			
	LCB	NA	NA	RT	NA	PM	NA			
	Add.	NA	NA			Ext. Visco	NA			
	MFI	0.4	4	Comp.	20/80; 30/70; 40/60; 60/40; 70/30; 80/20	Others	NA			
	$\eta_{0}\;(T)$	NA	NA							

**Table 4:** Rheological characterization of HDPE/LLDPE blends.

HDPE/LLDPE										
Study Number	Material Information			Processing Conditions		Rheological Characterization		Results and Discussion		Ref.
	Info.	Polymer 1 HDPE	Polymer 2 LLDPE					Rheological Observation	Authors Conclusion	
S1	ρ	0.959	0.902	Pr.	DSM Twin Screw extruder	AR	A	Do not follow log-additivity rules Sigmoidal behavior could indicate structural change in the molten state	Immiscible in molten state	[18]
	$\bar{M_{w}}$	174,000	104,000			TTS	NA			
	$\bar{M_{n}}$	14,000	50,000	T	220	Cross.	NA			
	PDI	NA	NA			CC P.	NA			
	Cat.	ZN	metallocene							
	Com.	Hexene	Octene	RPM	100	Han P.	NA			
	SCB	0.5 SCB/1000C	25 SCB/1000C			VGP	NA			
	LCB	X	X	RT	10	PM	NA			
	Add.	NA	NA			Ext. Visco	NA			
	MFI	NA	NA	Comp.	25/75; 50/50; 75/25	Others	NA			
	$\eta_{0}\;(T)$	115 at 180 °C	78 at 180 °C							
S2	ρ	0.937	0.918	Pr.	Single screw extruder	AR	A	AR: Small negative deviation for Han Plot: superposition blends with different composition CC P.: According to the composition, different slope	Probably miscible (depends on the composition)	[10]
	$\bar{M_{w}}$	NA	NA			TTS	NA			
	$\bar{M_{n}}$	NA	NA	T	190; 200; 220	Cross.	NA			
	PDI	NA	NA			CC P.	A			
	Cat.	NA	NA							
	Com.	Hexene	Hexane	RPM	80	Han P.	A			
	SCB	NA	NA			VGP	NA			
	LCB	X	X	RT	NA	PM	NA			
	Add.	NA	NA			Ext. Visco	NA			
	MFI	0.28	3.5	Comp.	20/80; 40/60; 60/40; 80/20	Others	NA			
	$\eta_{0}\;(T)$	NA	NA							
S2	ρ	0.937	0.918	Pr.	Single screw extruder	AR	A	AR: Small negative deviation Han P.: superposition blends with different composition CC P.: According to the composition, different slope	Probably miscible (depends on the composition)	[10]
	$\bar{M_{w}}$	NA	NA			TTS	NA			
	$\bar{M_{n}}$	NA	NA	T	190; 200; 220	Cross.	NA			
	PDI	NA	NA			CC P.	A			
	Cat.	NA	NA							
	Com.	Hexene	Butylene	RPM	80	Han P.	A			
	SCB	NA	NA			VGP	NA			
	LCB	X	X	RT	NA	PM	NA			
	Add.	NA	NA			Ext. Visco	NA			
	MFI	0.28	2.5	Comp.	20/80; 40/60; 60/40; 80/20	Others	NA			
	$\eta_{0}\;(T)$	NA	NA							
S3	ρ	NA	NA	Pr.	PRISM 16-mm co-rotating twin screw extruder	AR	A	Double reptation model: good fit	Miscible	[38]
	$\bar{M_{w}}$	40,000	68,320			TTS	NA			
	$\bar{M_{n}}$	13,000	NA	T	160 °C in barrel zone and 155 °C for the die	Cross.	NA			
	PDI	3	3.2			CC P.	NA			
	Cat.	NA	NA							
	Com.	NA	Ethene/hexene	RPM	50	Han P.	NA			
	SCB	X	1 SCB/100C			VGP	NA			
	LCB	X	X	RT	NA	PM	A			
	Add.	NA	NA			Ext. Visco	NA			
	MFI	NA	NA	Comp.	10/90; 20/80; 40/60; 60/40; 80/20; 90/10	Others	Double reptation model of Tsenoglou and des cloiseaux (92–94)			
	$\eta_{0}\;(T)$	448 at 160 °C	3.330 at 160 °C							
S3	ρ	NA	NA	Pr.	PRISM 16-mm co-rotating twin screw extruder	AR	A	Double reptation model: good fit with a slight difference could be a consequence of a higher degree of branching	Miscible	[38]
	$\bar{M_{w}}$	40,000	134,000			TTS	NA			
	$\bar{M_{n}}$	13,000	NA	T	160 °C in barrel zone and 155 °C for the die	Cross.	NA			
	PDI	3	3.8			CC P.	NA			
	Cat.	NA	NA							
	Com.	NA	Ethene/hexene	RPM	50	Han P.	NA			
	SCB	X	1.6 SCB/100C			VGP	NA			
	LCB	X	X	RT	NA	PM	A			
	Add.	NA	NA			Ext. Visco	NA			
	MFI	NA	NA	Comp.	10/90; 20/80; 40/60; 60/40; 80/20; 90/10	Others	Double reptation model of Tsenoglou and des cloiseaux (92–94)			
	$\eta_{0}\;(T)$	448 at 160 °C	37.800 at 160 °C							
S4	ρ	0.961	0.918	Pr.	Haake Polydrive melt blender	AR	A	AR: follows log-additivity rules PM: interfacial tension is equal to zero for the different compositions No influence of the composition distribution No influence of catalysts	Miscible	[31]
	$\bar{M_{w}}$	102,000	108,000			TTS	NA			
	$\bar{M_{n}}$	15,200	39,000	T	190	Cross.	NA			
	PDI	6.7	3.1			CC P.	NA			
	Cat.	NA	ZN							
	Com.	X	Butene	RPM	50	Han P.	NA			
	SCB	X	13.2 ${CH}_{3}$/1000C			VGP	NA			
	LCB	0	X	RT	10 min	PM	A			
	Add.	NA	NA			Ext. Visco	NA			
	MFI	0.7	1.0	Comp.	10/90; 30/70; 50/50; 70/30; 90/10	Others	First normal stress difference			
	$\eta_{0}\;(T)$	NA	NA							
S4	ρ	0.961	0.910	Pr.	Haake Polydrive melt blender	AR	A	AR: follows log-additivity rules PM: interfacial tension is equal to zero for the different compositions No influence of the composition distribution No influence of catalysts	Miscible	[31]
	$\bar{M_{w}}$	102,000	108,000			TTS	NA			
	$\bar{M_{n}}$	15,200	55,000	T	190	Cross.	NA			
	PDI	6.7	2.0			CC P.	NA			
	Cat.	NA	Metallocene							
	Com.	X	Butene	RPM	50	Han P.	NA			
	SCB	X	14.5 ${CH}_{3}$/1000C			VGP	NA			
	LCB	0	X	RT	10	PM	A			
	Add.	NA	NA			Ext. Visco	NA			
	MFI	0.7	1.2	Comp.	10/90; 30/70; 50/50; 70/30; 90/10	Others	First normal stress difference			
	$\eta_{0}\;(T)$	NA	NA							
S4	ρ	0.961	0.8800	Pr.	Haake Polydrive melt blender	AR	A	AR: Negative deviation Cross model: immiscibility in LLDPE-rich blend PM: poor fitting for LLDPE-rich blend Influence of high branching content on the miscibility	Immiscibility for LLDPE rich blends	[31]
	$\bar{M_{w}}$	102,000	125,000			TTS	NA			
	$\bar{M_{n}}$	15,200	69,400	T	190	Cross.	NA			
	PDI	6.7	1.8			CC P.	NA			
	Cat.	NA	Metallocene							
	Com.	X	Butene	RPM	50	Han P.	NA			
	SCB	X	42 ${CH}_{3}$/1000C			VGP	NA			
	LCB	0	X	RT	10	PM	A			
	Add.	NA	NA			Ext. Visco	NA			
	MFI	0.7	0.80	Comp.	10/90; 30/70; 50/50; 70/30; 90/10	Others	First normal stress difference, cross model			
	$\eta_{0}\;(T)$	NA	NA							
S5	ρ	0.961	0.910	Pr.	Haake Polydrive melt blender	AR	A	AR: follows log-additivity rules CC P.: semicircular shape + data for the blends lying between those of the pure resins No effect of type of comonomer	Miscible	[32]
	$\bar{M_{w}}$	102,000	108,000			TTS	NA			
	$\bar{M_{n}}$	NA	NA	T	190	Cross.	NA			
	PDI	NA	NA			CC P.	A			
	Cat.	NA	metallocene							
	Com.	X	Butene	RPM	50	Han P.	NA			
	SCB	X	14/1000C			VGP	NA			
	LCB	X	X	RT	10	PM	NA			
	Add.	1000 ppm of extra antioxidant	1000 ppm of extra antioxidant			Ext. Visco	NA			
	MFI	0.7	1.2	Comp.	10/90; 30/70; 50/50; 70/30; 90/10	Others	NA			
	$\eta_{0}\;(T)$	NA	NA							
S5	ρ	0.961	0.902	Pr.	Haake Polydrive melt blender	AR	A	AR: follows log-additivity rules CC P.: semicircular shape + data for the blends lying between those of the pure resins No effect of type of comonomer	Miscible	[32]
	$\bar{M_{w}}$	102,000	90,000			TTS	NA			
	$\bar{M_{n}}$	NA	NA	T	190	Cross.	NA			
	PDI	NA	NA			CC P.	A			
	Cat.	NA	Metallocene							
	Com.	X	Octene	RPM	50	Han P.	NA			
	SCB	X	17 ${CH}_{3}$/1000C			VGP	NA			
	LCB	X	X	RT	10	PM	NA			
	Add.	1000 ppm of extra antioxidant	1000 ppm of extra antioxidant			Ext. Visco	NA			
	MFI	0.7	1.2	Comp.	10/90; 30/70; 50/50; 70/30; 90/10	Others	NA			
	$\eta_{0}\;(T)$	NA	NA							
S6	ρ	0.961	0.9	Pr.	Haake Polydrive melt blender	AR	A	AR: follows log-additivity rules	Miscible	[39]
	$\bar{M_{w}}$	101.000	67.000			TTS	NA			
	$\bar{M_{n}}$	NA	NA	T	190	Cross.	NA			
	PDI	6.7	1.85			CC P.	NA			
	Cat.	NA	Metallocene							
	Com.	NA	Hexene	RPM	50	Han P.	NA			
	SCB	0	20.7 ${CH}_{3}$/1000C			VGP	NA			
	LCB	X	X	RT	10	PM	NA			
	Add.	1000 ppm of extra antioxidant	1000 ppm of extra antioxidant			Ext. Visco	NA			
	MFI	0.7	7.5	Comp.	10/90; 30/70; 50/50; 70/30; 90/10	Others	NA			
	$\eta_{0}\;(T)$	NA	NA							
S6	ρ	0.961	0.9	Pr.	Haake Polydrive melt blender	AR	A	AR: negative deviation	Immiscible	[39]
	$\bar{M_{w}}$	101.000	107.000			TTS	NA			
	$\bar{M_{n}}$	NA	NA	T	190	Cross.	NA			
	PDI	6.7	1.83			CC P.	NA			
	Cat.	NA	Metallocene							
	Com.	NA	Hexene	RPM	50	Han P.	NA			
	SCB	0	18 ${CH}_{3}$/1000C			VGP	NA			
	LCB	X	X	RT	10	PM	NA			
	Add.	1000 ppm of extra antioxidant	1000 ppm of extra antioxidant			Ext. Visco	NA			
	MFI	0.7	1.2	Comp.	10/90; 30/70; 50/50; 70/30; 90/10	Others	NA			
	$\eta_{0}\;(T)$	NA	NA							
S6	ρ	0.961	0.880	Pr.	Haake Polydrive melt blender	AR	A	AR: follows the log-additivity rules	Miscible	[39]
	$\bar{M_{w}}$	101,000	62,000			TTS	NA			
	$\bar{M_{n}}$	NA	NA	T	190 °C	Cross.	NA			
	PDI	6.7	2.01			CC P.	NA			
	Cat.	NA	Metallocene							
	Com.	NA	Butene	RPM	50	Han P.	NA			
	SCB	0	45 ${CH}_{3}$/1000C			VGP	NA			
	LCB	X	X	RT	10	PM	NA			
	Add.	1000 ppm of extra antioxidant	1000 ppm of extra antioxidant			Ext. Visco	NA			
	MFI	0.7	10	Comp.	10/90; 30/70; 50/50; 70/30; 90/10	Others	NA			
	$\eta_{0}\;(T)$	NA	NA							
S6	ρ	0.961	0.880	Pr.	Haake Polydrive melt blender	AR	A	AR: negative deviation	Immiscible	[39]
	$\bar{M_{w}}$	101,000	125,000			TTS	NA			
	$\bar{M_{n}}$	NA	NA	T	190	Cross.	NA			
	PDI	6.7	1.81			CC P.	NA			
	Cat.	NA	Metallocene							
	Com.	NA	Butene	RPM	50	Han P.	NA			
	SCB	0	42 ${CH}_{3}$/1000C			VGP	NA			
	LCB	X	X	RT	10	PM	NA			
	Add.	1000 ppm of extra antioxidant	1000 ppm of extra antioxidant			Ext. Visco	NA			
	MFI	0.7	0.8	Comp.	10/90; 30/70; 50/50; 70/30; 90/10	Others	NA			
	$\eta_{0}\;(T)$	NA	NA							
S7	ρ	NA	NA	Pr.	Thermo-Scientific HAAKE Polylab QC Rheomix	AR	A	AR: follows the log-additivity rules CC P.: Semicircular shape Han P: Supeposition of the curves for all compositions VGP: Superposition of the curves for all compositions + value of δ at the lowest G* is close to 90° No difference between hexene and butene	Miscible	[11]
	$\bar{M_{w}}$	NA	NA			TTS	NA			
	$\bar{M_{n}}$	NA	NA	T	180	Cross.	A			
	PDI	NA	NA			CC P.	A			
	Cat.	NA	NA							
	Com.	NA	butene	RPM	60	Han P.	A			
	SCB	NA	NA			VGP	A			
	LCB	NA	NA	RT	5	PM	NA			
	Add.	NA	NA			Ext. Visco	NA			
	MFI	4.5	2.7	Comp.	10/90; 30/70; 50/50; 70/30; 90/10	Others	Relaxation Spectrum			
	$\eta_{0}\;(T)$	NA	NA							
S7	ρ	NA	NA	Pr.	Thermo-Scientific HAAKE Polylab QC Rheomix	AR	A	AR: follows the log-additivity rules CC P.: Semicircular shape Han P: Supeposition of the curves for all compositions VGP: Superposition of the curves for all compositions + value of δ at the lowest G* is close to 90° No difference between hexene and butene	Miscible	[11]
	$\bar{M_{w}}$	NA	NA			TTS	NA			
	$\bar{M_{n}}$	NA	NA	T	180	Cross.	A			
	PDI	NA	NA			CC P.	A			
	Cat.	NA	NA							
	Com.	NA	hexene	RPM	60	Han P.	A			
	SCB	NA	NA			VGP	A			
	LCB	NA	NA	RT	5	PM	NA			
	Add.	NA	NA			Ext. Visco	NA			
	MFI	4.5	2.3	Comp.	10/90; 30/70; 50/50; 70/30; 90/10	Others	Relaxation Spectrum			
	$\eta_{0}\;(T)$	NA	NA							
S8	ρ	0.947	0.928	Pr.	Haake Rheomix600 mixer	AR	A	AR: follows the log-additivity rules	Miscible	[19]
	$\bar{M_{w}}$	83,100	45,700			TTS	NA			
	$\bar{M_{n}}$	NA	NA	T	160	Cross.	NA			
	PDI	2.0	2.1			CC P.	NA			
	Cat.	Metallocene	Metallocene							
	Com.	NA	Butene	RPM	NA	Han P.	NA			
	SCB	0	2.7%			VGP	NA			
	LCB	X	X	RT	5 min	PM	NA			
	Add.	0.5% Irganox 1010	0.5% Irganox 1010			Ext. Visco	NA			
	MFI	2.2	1.7	Comp.	20/80; 40/60; 60/40; 80/20	Others	NA			
	$\eta_{0}\;(T)$	X	X							

Index: ρ (g/cm^3^); Mw and Mn (g/mol); Cat.: catalyst type; Com.: comonomer type; Add.: type of additive; MFI (g/10 min); $\eta_{0}\;(T)$ (Pa.s); Pr.: processing device type; T: temperature (°C); RT: residence time (min); Comp.: composition; AR: additivity rules; Cross.: crossover analysis; CC P.: Cole–Cole Plot; Han P. Han Plot; VGP: Van Gurp–Palmen Plot; PM: Palierne model; Ext Visco: extensional viscosity; A: information are available; NA: information not available.

**Table 5:** Rheological characterization of LDPE/LLDPE blends.

LDPE/LLDPE										
Study Number	Material Information			Processing Conditions		Rheological Characterization		Results and Discussion		Ref.
	Info.	Polymer 1 LDPE	Polymer 2 LLDPE					Rheological Observation	Authors Conclusion	
S3	ρ	NA	NA	Pr.	PRISM 16-mm co-rotating twin screw extruder	AR	A	Storage modulus: At low frequencies, G’ of all the blend compositions is above G’ of pure resins AR: Positive deviation Double reptation model; Fit with the model of partial miscible blends HDPE could be used as a compatibilizer between LLDPE and LDPE	Partial miscibility	[38]
	$\bar{M_{w}}$	388,400	134,000			TTS	NA			
	$\bar{M_{n}}$	NA	NA	T	160 °C in barrel zone and 155 °C for the die	Cross.	NA			
	PDI	10	3.8			CC P.	NA			
	Cat.	NA	NA							
	Com.	NA	Ethene/hexene	RPM	50	Han P.	NA			
	SCB	NA	1.6 SCB/100C			VGP	NA			
	LCB	3.2 CB/100C	X	RT	NA	PM	A			
	Add.	NA	NA			Ext. Visco	NA			
	MFI	NA	NA	Comp.	10/90; 20/80; 40/60; 60/40; 80/20; 90/10	Others	Double reptation model of Tsenoglou and des Cloiseaux (92–94)			
	$\eta_{0}\;(T)$	32,600 (160 °C)	37,800 at 160 °C							
S8	ρ	0.920	0.928	Pr.	Haake Rheomix600 mixer	AR	A	AR: Positive deviation	Immiscible	[19]
	$\bar{M_{w}}$	10,200	45,700			TTS	NA			
	$\bar{M_{n}}$	NA	NA	T	160	Cross.	NA			
	PDI	6.8	2.1			CC P.	NA			
	Cat.	NA	Metallocene							
	Com.	NA	Butene	RPM	NA	Han P.	NA			
	SCB	NA	2.7%			VGP	NA			
	LCB	5.5%	X	RT	5 min	PM	NA			
	Add.	0.5% Irganox 1010				Ext. Visco	NA			
	MFI	7.8	1.7	Comp.	20/80; 40/60; 60/40; 80/20	Others	NA			
	$\eta_{0}\;(T)$	X	X							
S11	ρ	0.919	0.900	Pr.	Single-screw extruder	AR	A	AR: Positive deviation after 50% of LDPE TTS: Until 20% of LDPE, good superposition VGP: Until 20% of LDPE, good superposition	Partially miscible Immiscibility at higher concentration of LDPE	[26]
	$\bar{M_{w}}$	NA	NA			TTS	A			
	$\bar{M_{n}}$	NA	NA	T	160	Cross.	NA			
	PDI	NA	NA			CC P.	NA			
	Cat.	NA	Metallocene							
	Com.	NA	Butane	RPM	25	Han P.	NA			
	SCB	NA	NA			VGP	A			
	LCB	NA	NA	RT	NA	PM	A			
	Add.	NA	NA			Ext. Visco	A			
	MFI	0.47	1.3	Comp.	LLDPE/LDPE 99/1, 95/5; 90/10; 80/20; 50/50; 25/75	Others	NA			
	$\eta_{0}\;(T)$	127,900 (150 °C)	11,383 (150 °C)							
S11	ρ	0.919	0.920	Pr.	Single-screw extruder	AR	A	AR: Positive deviation after 50% of LDPE TTS: Until 20% of LDPE, good superposition VGP: Until 20% of LDPE, good superposition	Partially miscible Immiscibility at higher concentration of LDPE	[26]
	$\bar{M_{w}}$	NA	NA			TTS	A			
	$\bar{M_{n}}$	NA	NA	T	160	Cross.	NA			
	PDI	NA	NA			CC P.	NA			
	Cat.	NA	Metallocene							
	Com.	NA	Octane	RPM	25	Han P.	NA			
	SCB	NA	NA			VGP	A			
	LCB	NA	NA	RT	NA	PM	A			
	Add.	NA	NA			Ext. Visco	A			
	MFI	0.47	1	Comp.	LLDPE/LDPE 99/1, 95/5; 90/10; 80/20; 50/50; 25/75	Others	NA			
	$\eta_{0}\;(T)$	127,900 (150 °C)	34,116 (150 °C)							
S11	ρ	0.919	0.917	Pr.	Single-screw extruder	AR	A	AR: Positive deviation after 50% of LDPE TTS: Until 20% of LDPE, good superposition VGP: Until 20% of LDPE, good superposition	Partially miscible Immiscibility at higher concentration of LDPE	[26]
	$\bar{M_{w}}$	NA	NA			TTS	A			
	$\bar{M_{n}}$	NA	NA	T	160	Cross.	NA			
	PDI	NA	NA			CC P.	NA			
	Cat.	NA	ZN							
	Com.	NA	Hexane	RPM	25	Han P.	NA			
	SCB	NA	NA			VGP	A			
	LCB	NA	NA	RT	NA	PM	A			
	Add.	NA	NA			Ext. Visco	A			
	MFI	0.47	1	Comp.	LLDPE/LDPE 99/1, 95/5; 90/10; 80/20; 50/50; 25/75	Others	NA			
	$\eta_{0}\;(T)$	127,900 (150 °C)	34,116 (150 °C)							
S11	ρ	0.919	0.920	Pr.	Single-screw extruder	AR	A	AR: Positive deviation after 50% of LDPE TTS: Until 10% of LDPE, good superposition VGP: Until 10% of LDPE, good superposition	Partially miscible Immiscibility even at lower concentration of LDPE	[26]
	$\bar{M_{w}}$	NA	NA			TTS	A			
	$\bar{M_{n}}$	NA	NA	T	160	Cross.	NA			
	PDI	NA	NA			CC P.	NA			
	Cat.	NA	ZN							
	Com.	NA	Octane	RPM	25	Han P.	NA			
	SCB	NA	NA			VGP	A			
	LCB	NA	NA	RT	NA	PM	A			
	Add.	NA	NA			Ext. Visco	A			
	MFI	0.47	1	Comp.	LLDPE/LDPE 99/1, 95/5; 90/10; 80/20; 50/50; 25/75	Others	NA			
	$\eta_{0}\;(T)$	127,900 (150 °C)	16,994 (150 °C)							
S12	ρ	0.923	0.9	Pr.	Haake Polydrive melt blender	AR	A	Storage modulus: At low frequencies G’ of all the blend composition are in between G’ of pure resin AR: weak positive deviation PM: suggest weak interface at LDPE-rich blends Normal stress: N1 increase in proportion to the more elastic component	Almost miscible in the all range of composition	[33]
	$\bar{M_{w}}$	100,000	67,000			TTS	NA			
	$\bar{M_{n}}$	NA	NA	T	190	Cross.	NA			
	PDI	4.14	1.85			CC P.	NA			
	Cat.	NA	Metallocene							
	Com.	NA	Hexene	RPM	50	Han P.	NA			
	SCB	7.8 ${CH}_{3}/1000C$	21.1 ${CH}_{3}/1000C$			VGP	NA			
	LCB		X	RT	10	PM	A			
	Add.	1000 ppm antioxidant				Ext. Visco	NA			
	MFI	1.2	7.5	Comp.	10/90; 20/80; 30/70; 50/50; 70/30; 80/20	Others	Normal stress difference			
	$\eta_{0}\;(T)$	NA	NA							
S12	ρ	0.923	0.9	Pr.	Haake Polydrive melt blender	AR	A	Storage modulus: At low frequencies G’ of all the LDPE-rich composition are above G’ of pure resin AR: positive deviation for 50/50 and LDPE-rich blends PM: suggest high interface Normal stress: The 50/50 and LDPE-rich blend are higher than LDPE	Partially miscible Total immiscibility for LDPE-rich blend	[33]
	$\bar{M_{w}}$	100,000	108,000			TTS	NA			
	$\bar{M_{n}}$	NA	NA	T	190	Cross.	NA			
	PDI	4.14	1.83			CC P.	NA			
	Cat.	NA	Metallocene							
	Com.	NA	Hexene	RPM	50	Han P.	NA			
	SCB	7.8 ${CH}_{3}/1000C$	18 ${CH}_{3}/1000C$			VGP	NA			
	LCB		X	RT	10	PM	A			
	Add.	1000 ppm antioxidant				Ext. Visco	NA			
	MFI	1.2	1.2	Comp.	10/90; 20/80; 30/70; 50/50; 70/30; 80/20	Others	Normal stress difference			
	$\eta_{0}\;(T)$	NA	NA							
S12	ρ	0.923	0.9	Pr.	Haake Polydrive melt blender	AR		Storage modulus: At low frequencies, G’ of blends with 50, 30 and 10% are higher than the pure resin. AR: positive deviation for 50/50 and LDPE-rich blends PM: suggest high interface Normal stress: The 50/50 and LDPE-rich blend are higher than LDPE No difference of behavior between hexene and butene copolymer	Partially miscible Total immiscibility for LDPE-rich blend	[33]
	$\bar{M_{w}}$	100,000	110,000			TTS	A			
	$\bar{M_{n}}$	NA	NA	T	190	Cross.	NA			
	PDI	4.14	1.78			CC P.	NA NA			
	Cat.	NA	Metallocene							
	Com.	NA	Butene	RPM	50	Han P.				
	SCB	7.8 ${CH}_{3}/1000C$	20.4 ${CH}_{3}/1000C$			VGP	NA			
	LCB		X	RT	10	PM	NA			
	Add.	1000 ppm antioxidant				Ext. Visco	A			
	MFI	1.2	1.2	Comp.	10/90; 20/80; 30/70; 50/50; 70/30; 80/20	Others	NA Normal stress difference			
	$\eta_{0}\;(T)$	NA	NA							
S13	ρ	0.923	0.912	Pr.	Haake Polydrive melt blender	AR	A	Storage modulus: G’ of LLDPE rich blend are in between G’ of pure resins, whereas LDPE rich blend are above G’ of pure resin. AR: Positive deviation, mostly for LDPE rich blend Normal stress: The 50/50 and LDPE-rich blend are higher than LDPE	Partially miscible Total immiscibility for LDPE-rich blend	[40]
	$\bar{M_{w}}$	100,000	102,000			TTS	NA			
	$\bar{M_{n}}$	NA	Na	T	190	Cross.	NA			
	PDI	4.14	2.14			CC P.	NA			
	Cat.	NA	Metallocene							
	Com.	NA	Hexene	RPM	50	Han P.	NA			
	SCB	11 ${CH}_{3}/1000C$	14.4 ${CH}_{3}/1000C$			VGP	NA			
	LCB		X	RT	10	PM	NA			
	Add.	1000 ppm of antioxidant				Ext. Visco	NA			
	MFI	1.2	1.2	Comp.	10/90; 20/80; 30/70; 50/50; 70/30; 80/20	Others	Normal stress difference			
	$\eta_{0}\;(T)$	NA	NA							
S13	ρ	0.923	0.883	Pr.	Haake Polydrive melt blender	AR	A	Storage modulus: G’ of all the blend composition are in between G’ of pure resins. AR: Weak positive deviation Normal stress: N1 increase with increase in concentration of LDPE + N1 of all blends are in between the N1 of pure resins.	Almost miscible in the whole composition range	[40]
	$\bar{M_{w}}$	100,000	97,000			TTS	NA			
	$\bar{M_{n}}$	NA	NA	T	190	Cross.	NA			
	PDI	4.14	2.02			CC P.	NA			
	Cat.	NA	Metallocene							
	Com.	NA	Hexene	RPM	50	Han P.	NA			
	SCB	11 ${CH}_{3}/1000C$	32.2 ${CH}_{3}/1000C$			VGP	NA			
	LCB		X	RT	10	PM	NA			
	Add.	1000 ppm of antioxidant				Ext. Visco	NA			
	MFI	1.2	2.2	Comp.	10/90; 20/80; 30/70; 50/50; 70/30; 80/20	Others	Normal stress difference			
	$\eta_{0}\;(T)$	NA	NA							
S13	ρ	0.923	0.917	Pr.	Haake Polydrive melt blender	AR	A	Storage modulus: G’ of all the blend composition are in between G’ of pure resins. AR: Weak positive deviation LDPE-rich blend and almost follw the log-additivity rules for the other compositions ZN-LLDPE and LDPE are more miscible than m-LLDPE and LDPE	Miscible	[40]
	$\bar{M_{w}}$	100,000	107,000			TTS	NA			
	$\bar{M_{n}}$	NA	NA	T	190	Cross.	NA			
	PDI	4.14	4.01			CC P.	NA			
	Cat.	NA	ZN							
	Com.	NA	Hexene	RPM	50	Han P.	NA			
	SCB	11 ${CH}_{3}/1000C$	16.7 ${CH}_{3}/1000C$			VGP	NA			
	LCB		X	RT	10	PM	NA			
	Add.	1000 ppm of antioxidant				Ext. Visco	NA			
	MFI	1.2	2.8	Comp.	10/90; 20/80; 30/70; 50/50; 70/30; 80/20	Others	Normal stress difference			
	$\eta_{0}\;(T)$	NA	NA							
S14	ρ	0.918	0.924	Pr.	Haake Rheocord 90	AR	A	AR: follow the log-additivity rules for all the blend composition	Miscibility	[34]
	$\bar{M_{w}}$	71,800	50,800			TTS	NA			
	$\bar{M_{n}}$	13,400	14,700	T	190	Cross.	NA			
	PDI	5.37	3.46			CC P.	NA			
	Cat.	NA	ZN							
	Com.	NA	Butene	RPM	50	Han P.	NA			
	SCB	23 ${CH}_{3}/1000C$	20.7 ${CH}_{3}/1000C$			VGP	NA			
	LCB		X	RT	10	PM	A			
	Add.	1000 ppm of antioxidant mixture				Ext. Visco	NA			
	MFI	7	20	Comp.	10/90; 30/70; 50/50; 70/30; 90/10	Others	NA			
	$\eta_{0}\;(T)$	NA	NA							
S14	ρ	0.919	0.918	Pr.	Haake Rheocord 90	AR	A	AR: Strong positive deviation for LDPE-rich and 50/50 blends PM: Strong interfacial tension for LDPE-rich blends	Partial miscibility Miscibility in LLDPE-rich blends Immiscibility in LDPE-rich blends	[34]
	$\bar{M_{w}}$	99,500	105,300			TTS	NA			
	$\bar{M_{n}}$	15,500	29,500	T	190	Cross.	NA			
	PDI	6.45	3.56			CC P.	NA			
	Cat.	NA	ZN							
	Com.	NA	Butene	RPM	50	Han P.	NA			
	SCB	22 ${CH}_{3}/1000C$	22.1 ${CH}_{3}/1000C$			VGP	NA			
	LCB		X	RT	10	PM	A			
	Add.	1000 ppm of antioxidant mixture				Ext. Visco	NA			
	MFI	0.75	1.0	Comp.	10/90; 30/70; 50/50; 70/30; 90/10	Others	NA			
	$\eta_{0}\;(T)$	NA	NA							
S14	ρ	0.919	0.920	Pr.	Haake Rheocord 90	AR	A	AR: Strong positive deviation for LDPE-rich and 50/50 blends PM: Strong interfacial tension for LDPE-rich blends	Partial miscibility Miscibility in LLDPE-rich blendsImmiscibility in LDPE-rich blends	[34]
	$\bar{M_{w}}$	99,500	106,000			TTS	NA			
	$\bar{M_{n}}$	15,500	17,000	T	190	Cross.	NA			
	PDI	6.45	6.2			CC P.	NA			
	Cat.	NA	ZN							
	Com.	NA	Octene	RPM	50	Han P.	NA			
	SCB	22 ${CH}_{3}/1000C$	14.7 ${CH}_{3}/1000C$			VGP	NA			
	LCB		X	RT	10	PM	A			
	Add.	1000 ppm of antioxidant mixture				Ext. Visco	NA			
	MFI	0.75	1.0	Comp.	10/90; 30/70; 50/50; 70/30; 90/10	Others	NA			
	$\eta_{0}\;(T)$	NA	NA							
S15	ρ	0.923	0.947	Pr.	Solution Blending in xylene	AR	A	AR: Positive deviation	Immiscible	[35]
	$\bar{M_{w}}$	96,500	111,000			TTS	NA			
	$\bar{M_{n}}$	NA	NA	T	X	Cross.	NA			
	PDI	4.85	2.28			CC P.	NA			
	Cat.	NA	Metallocene							
	Com.	NA	NA	RPM	X	Han P.	NA			
	SCB	11.7 ${CH}_{3}/1000C$	1.9 ${CH}_{3}/1000C$			VGP	NA			
	LCB	1.6 ${CH}_{3}/1000C$	0 ${CH}_{3}/1000C$	RT	X	PM	NA			
	Add.	NA	NA			Ext. Visco	NA			
	MFI	NA	NA	Comp.	10/90; 30/70; 50/50; 90/10	Others	NA			
	$\eta_{0}\;(T)$	NA	NA							
S15	ρ	0.923	0.931	Pr.	Solution Blending in xylene	AR	A	AR: Positive deviation	Immiscible	[35]
	$\bar{M_{w}}$	96,500	80,100			TTS	NA			
	$\bar{M_{n}}$	NA	NA	T	X	Cross.	NA			
	PDI	4.85	2.72			CC P.	NA			
	Cat.	NA	Metallocene							
	Com.	NA	NA	RPM	X	Han P.	NA			
	SCB	11.7 ${CH}_{3}/1000C$	5.8 ${CH}_{3}/1000C$			VGP	NA			
	LCB	1.6 ${CH}_{3}/1000C$	0 ${CH}_{3}/1000C$	RT	X	PM	NA			
	Add.	NA	NA			Ext. Visco	NA			
	MFI	NA	NA	Comp.	10/90; 30/70; 50/50; 90/10	Others	NA			
	$\eta_{0}\;(T)$	NA	NA							
S15	ρ	0.923	0.949	Pr.	Solution Blending in xylene	AR	A	AR: Positive deviation	Immiscible	[35]
	$\bar{M_{w}}$	96,500	138,600			TTS	NA			
	$\bar{M_{n}}$	NA	NA	T	X	Cross.	NA			
	PDI	4.85	6.49			CC P.	NA			
	Cat.	NA	Metallocene							
	Com.	NA	NA	RPM	X	Han P.	NA			
	SCB	11.7 ${CH}_{3}/1000C$	2.9 ${CH}_{3}/1000C$			VGP	NA			
	LCB	1.6 ${CH}_{3}/1000C$	0 ${CH}_{3}/1000C$	RT	X	PM	NA			
	Add.	NA	NA			Ext. Visco	NA			
	MFI	NA	NA	Comp.	10/90; 30/70; 50/50; 90/10	Others	NA			
	$\eta_{0}\;(T)$	NA	NA							

**Table 6:** Rheological characterization of HDPE/UHMWPE blends.

HDPE/UHMWPE										
Study Number	Material Information			Processing Conditions		Rheological Characterization		Results and Discussion		Ref.
	Info.	Polymer 1 HDPE	Polymer 2 UHMWPE					Rheological Observation	Authors Conclusion	
S16	ρ	0.96	0.92	Pr.	Standard Leistritz ZSE-18 twin-screw extruder	AR	**NA**	Storage modulus: At low frequencies, G’ of the blends are in between the G’ of pure resins Han P.: No superposition of all the blends	Immiscible	[37]
	$\bar{M_{w}}$	78,000	5,000,000			TTS	NA			
	$\bar{M_{n}}$	NA	NA	T	180–170	Cross.	NA			
	PDI	NA	NA			CC P.	NA			
	Cat.	NA	NA							
	Com.	NA	NA	RPM	80	Han P.	A			
	SCB	NA	NA			VGP	NA			
	LCB	NA	NA	RT	NA	PM	NA			
	Add.	NA	NA			Ext. Visco	NA			
	MFI	7.6	No measurable	Comp.	HDPE/UHMWPE 5/95; 10/90; 20/80; 30/70; 40/60; 50/50	Others	NA			
	$\eta_{0}\;(T)$	NA	NA							
S17	ρ	0.961	0.937	Pr.	Haake Rheomix 600 mixer	AR	**A**	AR: do not follow the log additivity rules (negative and positive deviation) CC P.: Semicircular shape for blends with 30% and 40% of UHMWPE Absence of semicircular shape for blend with 10% and 20% of UHMWPE Han P.: no superposition	Immiscible	[36]
	$\bar{M_{w}}$	74,000	2,500,000			TTS	NA			
	$\bar{M_{n}}$	NA	NA	T	190	Cross.	NA			
	PDI	NA	NA			CC P.	**A**			
	Cat.	NA	NA							
	Com.	NA	NA	RPM	NA	Han P.	**A**			
	SCB	NA	NA			VGP	NA			
	LCB	NA	NA	RT	10	PM	NA			
	Add.	NA	NA			Ext. Visco	NA			
	MFI	7.2	Not measurable	Comp.	HDPE/UHMWPE 90/10; 80/20; 70/30; 60/40	Others	NA			
	$\eta_{0}\;(T)$	NA	NA							

**Table 7:** Rheological characterization of LLDPE/UHMWPE blends.

LLDPE/UHMWPE										
Study Number	Material Information			Processing Conditions		Rheological Characterization		Results and Discussion		Ref.
	Info.	Polymer 1 LLDPE	Polymer 2 UHMWPE					Rheological Observation	Authors Conclusion	
S17	ρ	0.918	0.937	Pr.	Haake Rheomix 600 mixer	AR	A	AR: do not follow the log additivity rules (negative and positive deviation) CC P.: Semicircular shape for blends with 30% and 40% of UHMWPE Absence of semicircular shape for blend with 10% and 20% of UHMWPE Han P.: no superposition	Immiscible	[36]
	$\bar{M_{w}}$	96,000	2,500,000			TTS	NA			
	$\bar{M_{n}}$	NA	NA	T	190	Cross.	NA			
	PDI	NA	NA			CC P.	A			
	Cat.	NA	NA							
	Com.	NA	NA	RPM	NA	Han P.	A			
	SCB	NA	NA			VGP	NA			
	LCB	NA	NA	RT	10	PM	NA			
	Add.	NA	NA			Ext. Visco	NA			
	MFI	2.0	Not measurable	Comp.	LLDPE/UHMWPE 90/10; 80/20; 70/30; 60/40	Others	NA			
	$\eta_{0}\;(T)$	NA	NA							

**Table 8:** Rheological characterization of LDPE/UHMWPE blends.

LLDPE/UHMWPE										
Study Number	Material Information			Processing Conditions		Rheological Characterization		Results and Discussion		Ref.
	Info.	Polymer 1 LLDPE	Polymer 2 UHMWPE					Rheological Observation	Authors Conclusion	
S17	ρ	0.918	0.937	Pr.	Haake Rheomix 600 mixer	AR	A	AR: follows the log additivity rules CC P.: Semi-circular shape for all the blend composition Han P.: Superposition	Miscible	[36]
	$\bar{M_{w}}$	96,000	2,500,000			TTS	NA			
	$\bar{M_{n}}$	NA	NA	T	190	Cross.	NA			
	PDI	NA	NA			CC P.	A			
	Cat.	NA	NA							
	Com.	NA	NA	RPM	NA	Han P.	A			
	SCB	NA	NA			VGP	NA			
	LCB	NA	NA	RT	10	PM	NA			
	Add.	NA	NA			Ext. Visco	NA			
	MFI	2.0	Not measurable	Comp.	LLDPE/UHMWPE 90/10; 80/20; 70/30; 60/40	Others	NA			
	$\eta_{0}\;(T)$	NA	NA							

**Table 9:** Rheological characterization of HDPE/ULDPE blends.

HDPE/ULDPE										
Study Number	Material Information			Processing Conditions		Rheological Characterization		Results and Discussion		Ref.
	Info.	Polymer 1 HDPE	Polymer 2 ULDPE					Rheological Observation	Authors Conclusion	
S1	ρ	0.959	0.870	Pr.	DSM twin-screw extruder	AR	**A**	AR: Negative deviation for the ULDPE rich blend and positive deviation for the HDPE rich blend	Immiscible	[18]
	$\bar{M_{w}}$	174,000	150,000			TTS	NA			
	$\bar{M_{n}}$	14,000	75,000	T	220	Cross.	NA			
	PDI	NA	NA			CC P.	NA			
	Cat.	ZN	NA							
	Com.	Hexene	Octene	RPM	100	Han P.	NA			
	SCB	0.5 SCB/1000C	60 SCB/1000C			VGP	NA			
	LCB	X	X	RT	10	PM	NA			
	Add.	NA	NA			Ext. Visco	NA			
	MFI	NA	NA	Comp.	25/75; 50/50; 75/25	Others	NA			
	$\eta_{0}\;(T)$	115 at 180 °C	22							

**Table 10:** Rheological characterization of LLDPE/ULDPE blends.

LLDPE/ULDPE										
Study Number	Material Information			Processing Conditions		Rheological Characterization		Results and Discussion		Ref.
	Info.	Polymer 1 LLDPE	Polymer 2 ULDPE					Rheological Observation	Authors Conclusion	
S1	ρ	0.902	0.870	Pr.	DSM twin-screw extruder	AR	**A**	AR: Weak negative deviation until for the blend with 25% of LLDPE and strong positive deviation for all the blends after 25% of LLDPE	Immiscible	[18]
	$\bar{M_{w}}$	104,000	150,000			TTS	NA			
	$\bar{M_{n}}$	50,000	75,000	T	220	Cross.	NA			
	PDI	NA	NA			CC P.	NA			
	Cat.	metallocene	NA							
	Com.	Octene	Octene	RPM	100	Han P.	NA			
	SCB	25 SCB/1000C	60 SCB/1000C			VGP	NA			
	LCB	X	X	RT	10	PM	NA			
	Add.	NA	NA			Ext. Visco	NA			
	MFI	NA	NA	Comp.	25/75; 50/50; 75/25	Others	NA			
	$\eta_{0}\;(T)$	78 at 180 °C	22							

**Table 11:** Apparent miscibility of different types of PEs evaluated via linear viscoelastic data.

Polymer Pair	General Apparent Miscibility as Inferred from Rheological Measurements	Key Parameters Evaluated	Conclusions	References
HDPE/LDPE	Immiscible		All papers report apparent immiscibility	[10,11,17,20]
HDPE/LLDPE	Conflicting results	SCB < 15/1000C, Low Mw LLDPE	Higher Mw of LLDPE leads to phase separation	[10,11,19,31,32,38,39]
LDPE/LLDPE	Conflicting results	Mw of LLDPE, SCB content, hexene content, catalyst type (debated)	Higher SCB and hexene promote apparent miscibility	[26,33,34,35,40]
UHMWPE/LDPE	Miscible		Only one study	[36]
UHMWPE/LLDPE	Immiscible		Only one study	[36]
UHMWPE/HDPE	Immiscible		Only two studies	[36,37]

**Table 12:** Comparison of the different techniques to evaluate the crystalline structure and the references which use it for studying the crystallinity of polyethylene blends.

Technique	% Crystallinity	Crystalline Cell	Lamellar Thickness	Spherulite Size	Tc, Tm	Other	Ref.
DSC	X				X	Kinetics of crystallization	[10,18,19,20,26,32,34,36,37,39,48,52,53,54,64,65,81]
SSA						Cristals population	[48,53,65,67,68,69,70,71,72,73,74,75,76,77]
WAXS	X	X					[18,20,45,49,51]
SAXS			X				[18,20,45,49,51]
OM				X	X	Less precise than DSC for Tc and Tm as rely on visualisation	[52,53]
SEM			X	X			[38]
TEM			X	X		Better resolution than SEM but difficult sample preparation	[47,52,64,65,66]
AFM			X	X			[18,52,54]
Rheology						Crystalization under shear Can be coupled with spectroscopic analysis and WAXS and SAXS	[56,57,58,59,60,61,78]

**Table 13:** Crystallization and mechanical properties of HDPE/LDPE blends.

HDPE/LDPE										
Study Number	Material Information			Processing Conditions		Crystalization and Mechanical Properties		Results and Discussion		Ref.
	Info.	Polymer 1 HDPE	Polymer 2 LDPE					Observation	Authors Conclusion	
S2	ρ	0.937	0.921	Pr.	Single-screw extruder	DSC	A	DSC: For 80/20: An endotherm peak For other compositions: 2 melting endotherm peaks	Partial miscibility according to the composition	[10]
	$\bar{M_{w}}$	NA	NA			SSA	NA			
	$\bar{M_{n}}$	NA	NA	T	190; 200; 220	POM	NA			
	PDI	NA	NA			TEM	NA			
	Cat.	NA	NA							
	Com.	Hexene	NA	RPM	80	SAXS	NA			
	SCB	NA	NA			AFM	NA			
	LCB	X	NA	RT	NA					
	Add.	NA	NA			Mechanical charact.	NA			
	MFI	0.28	0.75	Comp.	20/80; 40/60; 60/40; 80/20	Others	NA			
	$\eta_{0}\;(T)$	NA	NA							
S10	ρ	0.952	0.922	Pr.	Haake Rheocord-900	DSC	A	DSC: % of crystallization decrease with the addition of LDPE One single endotherm peak Tensile properties: Decrease in tensile strength and tensile modulus with addition of LDPE Impact Strength: Impact strength increase with the addition of LDPE	Miscibility (co-crystallization)	[20]
	$\bar{M_{w}}$	NA	NA			SSA	NA			
	$\bar{M_{n}}$	NA	NA	T	230	POM	NA			
	PDI	NA	NA			TEM	NA			
	Cat.	NA	NA							
	Com.	NA	NA	RPM	NA	SAXS	NA			
	SCB	NA	NA			AFM	NA			
	LCB	NA	NA	RT	NA					
	Add.	NA	NA			Mechanical charact.	A			
	MFI	0.4	4	Comp.	20/80; 30/70; 40/60; 60/40; 70/30; 80/20	Others	WAXS			
	$\eta_{0}\;(T)$	NA	NA							
S25	ρ	6 different HDPEs with different Mw	2 different LLDPE with different value of LCB and SCB	Pr.	Dissolution in xylene	DSC	NA	SAXS: Co-crystallization at low branching content Phase separation ate high LCB content	Miscibility depending on the branching content	[42]
	$\bar{M_{w}}$					SSA	NA			
	$\bar{M_{n}}$			T	NA	POM	NA			
	PDI					TEM	MA			
	Cat.									
	Com.			RPM	NA	SAXS	NA			
	SCB					AFM	NA			
	LCB			RT	NA					
	Add.					Mechanical charact.	NA			
	MFI			Comp.	NA	Others	SANS			
	$\eta_{0}\;(T)$									
S26	ρ	NA	NA	Pr.	Prism TSE 24TC extruder	DSC	A	DSC and SAXS: 3 melting endotherms peaks Crystallization rate affects the pase separation Mechanical properties: Addition of HDPE increase tensile modulus and creep resistance of the blends	Partial Miscibility	[49]
	$\bar{M_{w}}$	58,000	117,000			SSA	NA			
	$\bar{M_{n}}$	NA	NA	T	80 to 180	POM	NA			
	PDI	6	9			TEM	NA			
	Cat.	NA	NA							
	Com.	NA	NA	RPM	225	SAXS	A			
	SCB	NA	NA			AFM	NA			
	LCB	0	1.9 LCB/1000C	RT	Na					
	Add.	NA	NA			Mechanical charact.	A			
	MFI	NA	NA	Comp.	HDPE%; 1, 5, 10, 20, 40, 80	Others				
	$\eta_{0}\;(T)$	NA	NA							

**Table 14:** Crystallization and mechanical properties of HDPE/LLDPE blends.

HDPE/LLDPE										
Study Number	Material Information			Processing Conditions		Crystallization and Mechanical Properties		Results and Discussion		Ref.
	Info.	Polymer 1 HDPE	Polymer 2 LLDPE					Observation	Authors Conclusion	
S1	ρ	0.959	0.902	Pr.	DSM twin-screw extruder	DSC	A	Partial miscibility in molten state entails Tm depression without necessarily implying co-crystallization and crystal thickness reduction Nucleating effect from interface of phase separated domains	Partial miscibility	[18]
	$\bar{M_{w}}$	174,000	104,000			SSA	NA			
	$\bar{M_{n}}$	14,000	50,000	T	220	POM	NA			
	PDI	NA	NA			TEM	NA			
	Cat.	ZN	metallocene							
	Com.	Hexene	Octene	RPM	100	SAXS	A			
	SCB	0.5 SCB/1000C	25 SCB/1000C			AFM	A			
	LCB	X	X	RT	10					
	Add.	NA	NA			Mechanical charact.	NA			
	MFI	NA	NA	Comp.	25/75; 50/50; 75/25	Others	NA			
	$\eta_{0}\;(T)$	115 at 180 °C	78 at 180 °C							
S2	ρ	0.937	0.918	Pr.	Single-screw extruder	DSC	A	One single melting endotherm for all blends	Co-crystallization	[10]
	$\bar{M_{w}}$	NA	NA			SSA	NA			
	$\bar{M_{n}}$	NA	NA	T	190; 200; 220	POM	NA			
	PDI	NA	NA			TEM	NA			
	Cat.	NA	NA							
	Com.	Hexene	Hexane	RPM	80	SAXS	NA			
	SCB	NA	NA			AFM	NA			
	LCB	X	X	RT	NA					
	Add.	NA	NA			Mechanical charact.	NA			
	MFI	0.28	3.5	Comp.	20/80; 40/60; 60/40; 80/20	Others	NA			
	$\eta_{0}\;(T)$	NA	NA							
S2	ρ	0.937	0.918	Pr.	Single-screw extruder	DSC	A	Co-crystallization depends of the composition Presence of 2 peaks for 40/60 HDPE-LLDPE butylene blend		[10]
	$\bar{M_{w}}$	NA	NA			SSA	NA			
	$\bar{M_{n}}$	NA	NA	T	190; 200; 220	POM	NA			
	PDI	NA	NA			TEM	NA			
	Cat.	NA	NA							
	Com.	Hexene	Butylene	RPM	80	SAXS	NA			
	SCB	NA	NA			AFM	NA			
	LCB	X	X	RT	NA					
	Add.	NA	NA			Mechanical charact.	NA			
	MFI	0.28	2.5	Comp.	20/80; 40/60; 60/40; 80/20	Others	NA			
	$\eta_{0}\;(T)$	NA	NA							
S5	ρ	0.961	0.910	Pr.	Haake Polydrive melt blender	DSC	A	DSC: Crystallinity blends follows additivity rule; HDPE melting peak shifts towards that of m-LLDPE when %m-LLDPE; Mechanical characterization: UTS for HDPE rich lower that predicted by linear additivity rule; % of crystallinity does not correlate with mechanical properties;	Partial Miscibility HDPE-rich: Phase separation	[32]
	$\bar{M_{w}}$	102,000	108,000			SSA	NA			
	$\bar{M_{n}}$	NA	NA	T	190	POM	NA			
	PDI	NA	NA			TEM	NA			
	Cat.	NA	metallocene							
	Com.	X	Butene	RPM	50	SAXS	NA			
	SCB	X	14/1000C			AFM	NA			
	LCB	X	X	RT	10					
	Add.	1000 ppm of extra antioxidant	1000 ppm of extra antioxidant			Mechanical charact.	A			
	MFI	0.7	1.2	Comp.	10/90; 30/70; 50/50; 70/30; 90/10	Others	NA			
	$\eta_{0}\;(T)$	NA	NA							
S5	ρ	0.961	0.902	Pr.	Haake Polydrive melt blender	DSC	A	DSC: Crystallinity blends follows additivity rule; HDPE melting peak shifts towards that of m-LLDPE when %m-LLDPE; Mechanical characterization: UTS for HDPE rich lower that predicted by linear additivity rule; % of crystallinity does not correlate with mechanical properties;	Partial Miscibility HDPE-rich: Phase separation	[32]
	$\bar{M_{w}}$	102,000	90,000			SSA	NA			
	$\bar{M_{n}}$	NA	NA	T	190	POM	NA			
	PDI	NA	NA			TEM	NA			
	Cat.	NA	Metallocene							
	Com.	X	Octene	RPM	50	SAXS	NA			
	SCB	X	17 ${CH}_{3}$/1000C			AFM	NA			
	LCB	X	X	RT	10					
	Add.	1000 ppm of extra antioxidant	1000 ppm of extra antioxidant			Mechanical charact.	A			
	MFI	0.7	1.2	Comp.	10/90; 30/70; 50/50; 70/30; 90/10	Others	NA			
	$\eta_{0}\;(T)$	NA	NA							
S6	ρ	0.961	0.9	Pr.	Haake Polydrive melt blender	DSC	A	Immiscibility increases with M_w_ and branch content HDPE rich blends present co-crystallization m-LLDPE rich blends present 2 melting peaks	Partial Miscibility	[39]
	$\bar{M_{w}}$	101,000	67,000			SSA	NA			
	$\bar{M_{n}}$	NA	NA	T	190	POM	NA			
	PDI	6.7	1.85			TEM	NA			
	Cat.	NA	Metallocene							
	Com.	NA	Hexene	RPM	50	SAXS	NA			
	SCB	0	20.7 ${CH}_{3}$/1000C			AFM	NA			
	LCB	X	X	RT	10					
	Add.	1000 ppm of extra antioxidant	1000 ppm of extra antioxidant			Mechanical charact.	A			
	MFI	0.7	7.5	Comp.	10/90; 30/70; 50/50; 70/30; 90/10	Others	NA			
	$\eta_{0}\;(T)$	NA	NA							
S6	ρ	0.961	0.9	Pr.	Haake Polydrive melt blender	DSC	A	Immiscibility increases with M_w_ and branch content HDPE rich blends present co-crystallization m-LLDPE rich blends present 2 melting peaks	Partial Miscibility	[39]
	$\bar{M_{w}}$	101,000	107,000			SSA	NA			
	$\bar{M_{n}}$	NA	NA	T	190	POM	NA			
	PDI	6.7	1.83			TEM	NA			
	Cat.	NA	Metallocene							
	Com.	NA	Hexene	RPM	50	SAXS	NA			
	SCB	0	18 ${CH}_{3}$/1000C			AFM	NA			
	LCB	X	X	RT	10					
	Add.	1000 ppm of extra antioxidant	1000 ppm of extra antioxidant			Mechanical charact.	A			
	MFI	0.7	1.2	Comp.	10/90; 30/70; 50/50; 70/30; 90/10	Others	NA			
	$\eta_{0}\;(T)$	NA	NA							
S6	ρ	0.961	0.880	Pr.	Haake Polydrive melt blender	DSC	A	Immiscibility increases with M_w_ and branch content HDPE rich blends present co-crystallization m-LLDPE rich blends present 2 melting peaks	Partial Miscibility	[39]
	$\bar{M_{w}}$	101,000	62,000			SSA	NA			
	$\bar{M_{n}}$	NA	NA	T	190	POM	NA			
	PDI	6.7	2.01			TEM	NA			
	Cat.	NA	Metallocene							
	Com.	NA	Butene	RPM	50	SAXS	NA			
	SCB	0	45 ${CH}_{3}$/1000C			AFM	NA			
	LCB	X	X	RT	10					
	Add.	1000 ppm of extra antioxidant	1000 ppm of extra antioxidant			Mechanical charact	A			
	MFI	0.7	10	Comp.	10/90; 30/70; 50/50; 70/30; 90/10	Others	NA			
	$\eta_{0}\;(T)$	NA	NA							
S6	ρ	0.961	0.880	Pr.	Haake Polydrive melt blender	DSC	A	Immiscibility increases with M_w_ and branch content HDPE rich blends present co-crystallization m-LLDPE rich blends present 2 melting peaks	Partial Miscibility	[39]
	$\bar{M_{w}}$	101,000	125,000			SSA	NA			
	$\bar{M_{n}}$	NA	NA	T	190	POM	NA			
	PDI	6.7	1.81			TEM	NA			
	Cat.	NA	Metallocene							
	Com.	NA	Butene	RPM	50	SAXS	NA			
	SCB	0	42 ${CH}_{3}$/1000C			AFM	NA			
	LCB	X	X	RT	10					
	Add.	1000 ppm of extra antioxidant	1000 ppm of extra antioxidant			Mechanical charact	A			
	MFI	0.7	0.8	Comp.	10/90; 30/70; 50/50; 70/30; 90/10	Others	NA			
	$\eta_{0}\;(T)$	NA	NA							
S18	ρ	NA	NA	Pr.	Solution blending in xylene and precipitating in acetone at −20 °C	DSC	A	Phase diagram was drawn Phase sepration in the melt	Immiscibility	[80]
	$\bar{M_{w}}$	98,000	208,000			SSA	NA			
	$\bar{M_{n}}$	28,200	25,300	T	NA	POM	NA			
	PDI	NA	NA			TEM	A			
	Cat.	NA	NA							
	Com.	NA	NA	RPM	NA	SAXS	NA			
	SCB	NA	16 SCB/1000C			AFM	NA			
	LCB	NA	10 LCB/1000C	RT	NA					
	Add.	NA	NA			Mechanical charact	NA			
	MFI	NA	NA	Comp.	All concentration	Others	Replica by using permanganic etching TEM by disoving xylene			
	$\eta_{0}\;(T)$	NA	NA							
S19	ρ	0.960	0.900	Pr.	Solution blending in xylene and precipitating in acetone at −20 °C	DSC	A	DSC: LLDPE rich blends: 2 melting peaks TEM: LLDPE-rich blends: 2 types of crystals species Occurrence of the 2 phases depends on the annealing temperature No influence of type of comonomer	Phase separation in the LLDPE rich blends	[64]
	$\bar{M_{w}}$	100,000	132,000			SSA	NA			
	$\bar{M_{n}}$	NA	NA	T	NA	POM	NA			
	PDI	3.5	1.95			TEM	A			
	Cat.	NA	NA							
	Com.	NA	Butene	RPM	NA	SAXS	NA			
	SCB	0	5.9 mol%			AFM	NA			
	LCB	0	NA	RT	NA					
	Add.	NA	NA			Mechanical charact	NA			
	MFI	5	5.9	Comp.	All concentration	Other	NA			
	$\eta_{0}\;(T)$	NA	NA							
S19	ρ	0.960	0.901	Pr.	Solution blending in xylene and precipitating in acetone at −20 °C	DSC	A	DSC: LLDPE rich blends: 2 melting peaks TEM: LLDPE-rich blends: 2 types of crystals species Occurrence of the 2 phases depends on the annealing temperature No influence of type of comonomer	Phase separation in the LLDPE rich blends	[64]
	$\bar{M_{w}}$	100,000	58,000			SSA	NA			
	$\bar{M_{n}}$	NA	NA	T	NA	POM	NA			
	PDI	3.5	2.65			TEM	A			
	Cat.	NA	NA							
	Com.	NA	Butene	RPM	NA	SAXS	NA			
	SCB	0	6.3 mol%			AFM	NA			
	LCB	0	NA	RT	NA					
	Add.	NA	NA			Mechanical charact.	NA			
	MFI	5	27	Comp.	All concentration	Other	NA			
	$\eta_{0}\;(T)$	NA	NA							
S19	ρ	0.960	0.895	Pr.	Solution blending in xylene and precipitating in acetone at −20 °C	DSC	A	DSC: LLDPE rich blends: 2 melting peaks TEM: LLDPE-rich blends: 2 types of crystals species Occurrence of the 2 phases depends on the annealing temperature No influence of type of comonomer	Phase separation in the LLDPE rich blends	[64]
	$\bar{M_{w}}$	100,000	90,000			SSA	NA			
	$\bar{M_{n}}$	NA	NA	T	NA	POM	NA			
	PDI	3.5	1.5			TEM	A			
	Cat.	NA	Metallocene							
	Com.	NA	Hexene	RPM	NA	SAXS	NA			
	SCB	0	6.3 mol%			AFM	NA			
	LCB	0	NA	RT	NA					
	Add.	NA	NA			Mechanical charact.	NA			
	MFI	5	16.5	Comp.	All concentration	Other	NA			
	$\eta_{0}\;(T)$	NA	NA							
S20	ρ	0.970	0.937	Pr.	Solution blending in xylene and precipitating in acetone at −20 °C	DSC	A	DSC: One single exotherm TEM and AFM: One single type of lamella	Miscible (1 crystal population)	[52]
	$\bar{M_{w}}$	26,000	90,000			SSA	NA			
	$\bar{M_{n}}$	NA	NA	T	NA	POM	A			
	PDI	5	2.7			TEM	A			
	Cat.	NA	Single site							
	Com.	NA	Butene	RPM	NA	SAXS	NA			
	SCB	0	1%mol			AFM	A			
	LCB	NA	NA	RT	NA					
	Add.	NA	NA			Mechanical charact.	NA			
	MFI	NA	NA	Comp.	10/90	Other	NA			
	$\eta_{0}\;(T)$	NA	NA							
S20	ρ	0.970	0.925	Pr.	Solution blending in xylene and precipitating in acetone at −20 °C	DSC	A	DSC: One single exotherm TEM and AFM: One single type of lamella	Miscible (1 crystal population)	[52]
	$\bar{M_{w}}$	26,000	150,000			SSA	NA			
	$\bar{M_{n}}$	NA	NA	T	NA	POM	A			
	PDI	5	2.2			TEM	A			
	Cat.	NA	Single site							
	Com.	NA	Butene	RPM	NA	SAXS	NA			
	SCB	0	1.7 mol%			AFM	A			
	LCB	NA	NA	RT	NA					
	Add.	NA	NA			Mechanical charact.	NA			
	MFI	NA	NA	Comp.	10/90	Other	NA			
	$\eta_{0}\;(T)$	NA	NA							
S20	ρ	0.970	0.925	Pr.	Solution blending in xylene and precipitating in acetone at −20 °C	DSC	A	DSC: Two-crystal exotherm TEM and AFM: Two-crystal population Limited degree of co-crystallization	Immiscibility (phase separation)	[52]
	$\bar{M_{w}}$	26,000	115,000			SSA	NA			
	$\bar{M_{n}}$	NA	NA	T	NA	POM	A			
	PDI	5	2.5			TEM	A			
	Cat.	NA	Single site							
	Com.	NA	Butene	RPM	NA	SAXS	NA			
	SCB	0	1.8 mol%			AFM	A			
	LCB	NA	NA	RT	NA					
	Add.	NA	NA			Mechanical charact.	NA			
	MFI	NA	NA	Comp.	10/90	Other	NA			
	$\eta_{0}\;(T)$	NA	NA							
S20	ρ	0.970	0.928	Pr.	Solution blending in xylene and precipitating in acetone at −20 °C	DSC	A	DSC: Two-crystal exotherms Decrease in crystallization temperature of LLDPE rich phase TEM and AFM: Two-crystal population Lamellae in LLDPE rich component smaller than in pure LLDPE POM:Increasing % of HDPE disrupt spherulitic structure	Immiscibility (phase separation)	[52]
	$\bar{M_{w}}$	26,000	62,000			SSA	NA			
	$\bar{M_{n}}$	NA	NA	T	NA	POM	A			
	PDI	5	3.3			TEM	A			
	Cat.	NA	Single site							
	Com.	NA	Butene	RPM	NA	SAXS	NA			
	SCB	0	2.5 mol%			AFM	A			
	LCB	NA	NA	RT	NA					
	Add.	NA	NA			Mechanical charact.	NA			
	MFI	NA	NA	Comp.	10/90	Other	NA			
	$\eta_{0}\;(T)$	NA	NA							
S20	ρ	0.970	0.920	Pr.	Solution blending in xylene and precipitating in acetone at −20 °C	DSC	A	DSC: Two-crystal exotherm Decrease in crystallization temperature of LLDPE rich phase TEM and AFM: Two-crystal population Lamellae in LLDPE rich component smaller than in pure LLDPE POM: Increasing % of HDPE disrupt spherulitic structure	Immiscibility (phase separation)	[52]
	$\bar{M_{w}}$	26,000	105,000			SSA	NA			
	$\bar{M_{n}}$	NA	NA	T	NA	POM	A			
	PDI	5	2.2			TEM	A			
	Cat.	NA	Single site							
	Com.	NA	Butene	RPM	NA	SAXS	NA			
	SCB	0	4.2 mol%			AFM	A			
	LCB	NA	NA	RT	NA					
	Add.	NA	NA			Mechanical charact.	NA			
	MFI	NA	NA	Comp.	10/90	Other	NA			
	$\eta_{0}\;(T)$	NA	NA							
S20	ρ	0.970	0.902	Pr.	Solution blending in xylene and precipitating in acetone at −20 °C	DSC	A	DSC: Two-crystal exotherm Decrease in crystallization temperature of LLDPE rich phase TEM and AFM: Two-crystal population Lamellae in LLDPE rich component smaller than in pure LLDPE POM:Increasing % of HDPE disrupt spherulitic structure	Immiscibility (phase separation)	[52]
	$\bar{M_{w}}$	26,000	85,000			SSA	NA			
	$\bar{M_{n}}$	NA	NA	T	NA	POM	A			
	PDI	5	2.2			TEM	A			
	Cat.	NA	Single site							
	Com.	NA	Butene	RPM	NA	SAXS	NA			
	SCB	0	5.7 mol%			AFM	A			
	LCB	NA	NA	RT	NA					
	Add.	NA	NA			Mechanical charact.	NA			
	MFI	NA	NA	Comp.	10/90	Other	NA			
	$\eta_{0}\;(T)$	NA	NA							
S20	ρ	0.970	0.903	Pr.	Solution blending in xylene and precipitating in acetone at −20 °C	DSC	A	DSC: Two-crystal exotherm Decrease in crystallization temperature of LLDPE rich phase TEM and AFM: Two-crystal population Lamellae in LLDPE rich component smaller than in pure LLDPE POM: Increasing % of HDPE disrupt spherulitic structure	Immiscibility (phase separation)	[52]
	$\bar{M_{w}}$	26,000	58,000			SSA	NA			
	$\bar{M_{n}}$	NA	NA	T	NA	POM	A			
	PDI	5	10			TEM	A			
	Cat.	NA	Single site							
	Com.	NA	Butene	RPM	NA	SAXS	NA			
	SCB	0	7.5 mol%			AFM	A			
	LCB	NA	NA	RT	NA					
	Add.	NA	NA			Mechanical charact.	NA			
	MFI	NA	NA	Comp.	10/90	Other	NA			
	$\eta_{0}\;(T)$	NA	NA							
S21	ρ	0.968	0.922	Pr.	Haake Rheocord EU10 co-rotating double screw	DSC	A	DSC: Two-crystal exotherm peaks SSA: Nucleating effect of HDPE on LLDPE Molecular segregation without significant miscibility	Immiscibility (phase separation)	[81]
	$\bar{M_{w}}$	168,000	159,700			SSA	A			
	$\bar{M_{n}}$	28,500	37,800	T	210	POM	NA			
	PDI	NA	NA			TEM	NA			
	Cat.	NA	NA							
	Com.	NA	Butene	RPM	40	SAXS	NA			
	SCB	NA	12.7 CH_3_/1000C			AFM	NA			
	LCB	NA	NA	RT	NA					
	Add.	NA	NA			Mechanical charact.	NA			
	MFI	5.8	1.4	Comp.	20/80; 30/70; 50/50; 70/30; 80/20	Other	NA			
	$\eta_{0}\;(T)$	NA	NA							
S21	ρ	0.922	0.922	Pr.	Haake Rheocord EU10 co-rotating double screw	DSC	A	DSC: Two-crystal exotherm peaks SSA: Partial miscibility between the chains of specific SCB of each copolymer for compositions of LLDPE-rich blends	Partial miscibility	[81]
	$\bar{M_{w}}$	23,000	159,700			SSA	A			
	$\bar{M_{n}}$	12,500	37,800	T	210	POM	NA			
	PDI	NA	NA			TEM	NA			
	Cat.	NA	NA							
	Com.	Butene	Butene	RPM	40	SAXS	NA			
	SCB	4.4 CH_3_/1000C	12.7 CH_3_/1000C			AFM	NA			
	LCB	NA	NA	RT	NA					
	Add.	NA	NA			Mechanical charact.	NA			
	MFI	1.4	1.4	Comp.	20/80; 30/70; 50/50; 70/30; 80/20	Other	NA			
	$\eta_{0}\;(T)$	NA	NA							
S22	ρ	0.968	0.888	Pr.	Haake Rheocord EU10	DSC	A	DSC: Simple cooling: Two crystal exotherm peaks Fast cooling: one single crystal peaks SSA and TEM: Fas cooling: Nycleation of LLDPE by HDPE Melt point depression of HDPE rich phase, dilution effect	Miscibility depends on the speed of cooling Standard cooling: Phase separation Fast cooling: Co-crystallization	[65]
	$\bar{M_{w}}$	168,000	NA			SSA	A			
	$\bar{M_{n}}$	28,500	23,000	T	210	POM	NA			
	PDI	NA	NA			TEM	A			
	Cat.	NA	NA							
	Com.	NA	NA	RPM	40	SAXS	NA			
	SCB	NA	NA			AFM	NA			
	LCB	NA	NA	RT	NA					
	Add.	NA	NA			Mechanical charact.	NA			
	MFI	5.8	2.05	Comp.	20/80; 30/70; 50/50; 70/30; 80/20	Other	NA			
	$\eta_{0}\;(T)$	NA	NA							
S23	ρ	0.947	0.928	Pr.	Haake Rheomix	DSC	A	DSC: One single exotherm peak	Miscible	[19]
	$\bar{M_{w}}$	83,100	45,700			SSA	NA			
	$\bar{M_{n}}$	NA	NA	T	160	POM	NA			
	PDI	2.0	2.1			TEM	NA			
	Cat.	Metallocene	Metallocene							
	Com.	NA	NA	RPM	NA	SAXS	NA			
	SCB	0	2.7 CH_3_/1000C			AFM	NA			
	LCB	0	NA	RT	NA					
	Add.	NA	NA			Mechanical charact.	NA			
	MFI	2.2	1.7	Comp.	20/80; 30/70; 50/50; 70/30; 80/20	Other	NA			
	$\eta_{0}\;(T)$	NA	NA							
S24	ρ	0.960	0.918	Pr.	Haake PolylabOS batch mixer	DSC	A	DSC: Reduction of H Tm of HDPE One single peak		[54]
	$\bar{M_{w}}$	124,000	134,000			SSA	NA			
	$\bar{M_{n}}$	NA	NA	T	180	POM	NA			
	PDI	3.3	2.2			TEM	NA			
	Cat.	NA	NA							
	Com.	NA	NA	RPM	150	SAXS	NA			
	SCB	NA	NA			AFM	A			
	LCB	NA	NA	RT	NA					
	Add.	NA	NA			Mechanical charact.	NA			
	MFI	4	3.5	Comp.	20/80; 80/20	Other	NA			
	$\eta_{0}\;(T)$	NA	NA							

**Table 15:** Crystallization and mechanical properties of LDPE/LLDPE blends.

				LDPE/LLDPE						
Study Number	Material Information			Processing Conditions		Crystalization and Mechanical Properties		Results and Discussion		Ref.
	Info.	Polymer 1 LDPE	Polymer 2 LLDPE					Observation	Authors Conclusion	
S11	ρ	0.919	0.900	Pr.	Single screw extruder	DSC	A	DSC: One single endotherm peaks	Miscibility (co-crystallization)	[26]
	$\bar{M_{w}}$	NA	NA			SSA	NA			
	$\bar{M_{n}}$	NA	NA	T	160	POM	NA			
	PDI	NA	NA			TEM	NA			
	Cat.	NA	Metallocene							
	Com.	NA	Butane	RPM	25	SAXS	NA			
	SCB	NA	NA			AFM	NA			
	LCB	NA	NA	RT	NA					
	Add.	NA	NA			Mechanical charact.	NA			
	MFI	0.47	1.3	Comp.	LLDPE/LDPE 99/1, 95/5; 90/10; 80/20; 50/50; 25/75	Others	NA			
	$\eta_{0}\;(T)$	127,900 (150 °C)	11,383 (150 °C)							
S11	ρ	0.919	0.920	Pr.	Single screw extruder	DSC	A	DSC: One single melting peaks	Miscibility (co-crystallization)	[26]
	$\bar{M_{w}}$	NA	NA			SSA	NA			
	$\bar{M_{n}}$	NA	NA	T	160	POM	NA			
	PDI	NA	NA			TEM	NA			
	Cat.	NA	Metallocene							
	Com.	NA	Octane	RPM	25	SAXS	NA			
	SCB	NA	NA			AFM	NA			
	LCB	NA	NA	RT	NA					
	Add.	NA	NA			Mechanical charact.	NA			
	MFI	0.47	1	Comp.	LLDPE/LDPE 99/1, 95/5; 90/10; 80/20; 50/50; 25/75	Others	NA			
	$\eta_{0}\;(T)$	127,900 (150 °C)	34,116 (150 °C)							
S11	ρ	0.919	0.920	Pr.	Single screw extruder	DSC	A	DSC: Three melting peaks Better miscibility with ZN-octane LLDPE	Partial Miscibility with Phase speration, but possibility of co-crystallization at the interface	[26]
	$\bar{M_{w}}$	NA	NA			SSA	NA			
	$\bar{M_{n}}$	NA	NA	T	160	POM	NA			
	PDI	NA	NA			TEM	NA			
	Cat.	NA	Metallocene							
	Com.	NA	Octane	RPM	25	SAXS	NA			
	SCB	NA	NA			AFM	NA			
	LCB	NA	NA	RT	NA					
	Add.	NA	NA			Mechanical charact.	NA			
	MFI	0.47	1	Comp.	LLDPE/LDPE 99/1, 95/5; 90/10; 80/20; 50/50; 25/75	Others	NA			
	$\eta_{0}\;(T)$	127,900 (150 °C)	34,116 (150 °C)							
S11	ρ	0.919	0.917	Pr.	Single screw extruder	DSC	A	DSC: Three melting peaks at lower LDPE content	Partial Miscibility with Phase separation, but possibility of co-crystallization at the interface	[26]
	$\bar{M_{w}}$	NA	NA			SSA	NA			
	$\bar{M_{n}}$	NA	NA	T	160	POM	NA			
	PDI	NA	NA			TEM	NA			
	Cat.	NA	ZN							
	Com.	NA	Hexane	RPM	25	SAXS	NA			
	SCB	NA	NA			AFM	NA			
	LCB	NA	NA	RT	NA					
	Add.	NA	NA			Mechanical charact.	NA			
	MFI	0.47	1	Comp.	LLDPE/LDPE 99/1, 95/5; 90/10; 80/20; 50/50; 25/75	Others	NA			
	$\eta_{0}\;(T)$	127,900 (150 °C)	34,116 (150 °C)							
S14	ρ	0.918	0.924	Pr.	Haake Rheocord 90	DSC	A	DSC: Influence of composition in the co-crystallization 10% of LDPE: 2 melting peaks From 50% to 90% of LDPE: 1 single melting peaks	Partial Miscibility	[34]
	$\bar{M_{w}}$	71,800	50,800			SSA	NA			
	$\bar{M_{n}}$	13,400	14,700	T	190	POM	NA			
	PDI	5.37	3.46			TEM	NA			
	Cat.	NA	ZN							
	Com.	NA	Butene	RPM	50	SAXS	NA			
	SCB	23 ${CH}_{3}/1000C$	20.7 ${CH}_{3}/1000C$			AFM	NA			
	LCB		X	RT	10					
	Add.	1000 ppm of antioxidant mixture				Mechanical charact.	NA			
	MFI	7	20	Comp.	10/90; 30/70; 50/50; 70/30; 90/10	Others	NA			
	$\eta_{0}\;(T)$	NA	NA							
S14	ρ	0.918	0.918	Pr.	Haake Rheocord 90	DSC	A	DSC: Influence of composition in the co-crystallization Until 70% of LDPE: 2 melting peaks At 90% of LDPE: 1 single melting peaks	Partial Miscibility	[34]
	$\bar{M_{w}}$	71,800	105,300			SSA	NA			
	$\bar{M_{n}}$	13,400	29,500	T	190	POM	NA			
	PDI	5.37	3.56			TEM	NA			
	Cat.	NA	ZN							
	Com.	NA	Butene	RPM	50	SAXS	NA			
	SCB	23 ${CH}_{3}/1000C$	22.1 ${CH}_{3}/1000C$			AFM	NA			
	LCB		X	RT	10					
	Add.	1000 ppm of antioxidant mixture				Mechanical charact.	NA			
	MFI	7	1.0	Comp.	10/90; 30/70; 50/50; 70/30; 90/10	Others	NA			
	$\eta_{0}\;(T)$	NA	NA							
S14	ρ	0.918	0.920	Pr.	Haake Rheocord 90	DSC	A	DSC: Influence of composition in the co-crystallization At 10% of LDPE: 2 melting peaks At 50% of LDPE: 3 melting peaks At 90% of LDPE: 1 single melting peaks	Partial Miscibility	[34]
	$\bar{M_{w}}$	71,800	106,000			SSA	NA			
	$\bar{M_{n}}$	13,400	17,000	T	190	POM	NA			
	PDI	5.37	6.2			TEM	NA			
	Cat.	NA	ZN							
	Com.	NA	Octene	RPM	50	SAXS	NA			
	SCB	23 ${CH}_{3}/1000C$	14.7 ${CH}_{3}/1000C$			AFM	NA			
	LCB		X	RT	10					
	Add.	1000 ppm of antioxidant mixture				Mechanical charact.	NA			
	MFI	7	1.0	Comp.	10/90; 30/70; 50/50; 70/30; 90/10	Others	NA			
	$\eta_{0}\;(T)$	NA	NA							
S23	ρ	0.920	0.928	Pr.	Haake Rheomix 600	DSC	A	DSC: Influence of composition Above 40% of LDPE: 2 melting peaks	Partial Miscibility	[19]
	$\bar{M_{w}}$	102,000	45,700			SSA	NA			
	$\bar{M_{n}}$	NA	NA	T	160	POM	NA			
	PDI	NA	2.1			TEM	NA			
	Cat.	NA	Metallocene							
	Com.	NA	NA	RPM	NA	SAXS	NA			
	SCB	NA	2.7 SCB/1000C			AFM	NA			
	LCB	5.5 LCB/1000C	NA	RT	NA					
	Add.	NA	NA			Mechanical charact.	NA			
	MFI	7.8	1.7	Comp.	20/80;40/60; 60/40; 80/20	Others	NA			
	$\eta_{0}\;(T)$	NA	NA							
S27	ρ	0.919	0.901	Pr.	Melt extrusion with a single screw	DSC	A	DSC and SSA: 1 single melting peaks Value of Tm do not follow perfectly the log additivity rules	Miscibility: Co-crystallization	[48]
	$\bar{M_{w}}$	474,000	58,000			SSA	N			
	$\bar{M_{n}}$	NA	NA	T	140 to 200	POM	NA			
	PDI	23.3	2.65			TEM	NA			
	Cat.	Peroxide NA	Metallocene							
	Com.	NA	Butene	RPM	60	SAXS	NA			
	SCB	NA	6.3 mol%			AFM	NA			
	LCB	NA	NA	RT	NA					
	Add.	NA	NA			Mechanical charact.	NA			
	MFI	7	27	Comp.	25/75; 30/70; 50/50; 70/30; 90/10	Others	NA			
	$\eta_{0}\;(T)$	NA	NA							
S27	ρ	0.918	0.901	Pr.	Melt extrusion with a single screw	DSC	A	DSC and SSA: 1 single melting peaks	Miscibility: Co-crystallization	[48]
	$\bar{M_{w}}$	89,000	58,000			SSA	N			
	$\bar{M_{n}}$	NA	NA	T	140 to 200	POM	NA			
	PDI	22	2.65			TEM	NA			
	Cat.	Peroxide NA	Metallocene							
	Com.	NA	Butene	RPM	60	SAXS	NA			
	SCB	NA	6.3 mol%			AFM	NA			
	LCB	NA	NA	RT	NA					
	Add.	NA	NA			Mechanical charact.	NA			
	MFI	22	27	Comp.	25/75; 30/70; 50/50; 70/30; 90/10	Others	NA			
	$\eta_{0}\;(T)$	NA	NA							

**Table 16:** Crystallization and mechanical properties of HDPE/ULDPE blends.

HDPE/ULDPE										
Study Number	Material Information			Processing Conditions		Crystalization and Mechanical Properties		Results and Discussion		Ref.
	Info.	Polymer 1 HDPE	Polymer 2 ULDPE					Observation	Authors Conclusion	
S1	ρ	0.959	0.870	Pr.	DSM twin-screw extruder	DSC	A	DSC and SAXS: Depending on the composition: 2 melting peaks AFM: Nucleating effect from interface phase	Partial Miscibility	[18]
	$\bar{M_{w}}$	174,000	150,000			SSA	NA			
	$\bar{M_{n}}$	14,000	75,000	T	220	POM	NA			
	PDI	NA	NA			TEM	NA			
	Cat.	ZN	NA							
	Com.	Hexene	Octene	RPM	100	SAXS	A			
	SCB	0.5 SCB/1000C	60 SCB/1000C			AFM	A			
	LCB	X	X	RT	10					
	Add.	NA	NA			Mechanical charact.	NA			
	MFI	NA	NA	Comp.	25/75; 50/50; 75/25	Others	DMTA			
	$\eta_{0}\;(T)$	115 at 180 °C	22							
S21	ρ	0.922	0.888	Pr.	Haake Rheocord EU10 co-rotating double-screw extruder	DSC	A	DSC: 2 melting peaks SSA: Nucleating effect of HDPE on ULDPE Molecular segregation without significant miscibility	Immiscibility	[81]
	$\bar{M_{w}}$	230,000	NA			SSA	A			
	$\bar{M_{n}}$	12,500	23,000	T	210	POM	NA			
	PDI	NA	NA			TEM	NA			
	Cat.	NA	MA							
	Com.	Butene	Butene	RPM	40	SAXS	NA			
	SCB	4.4 SCB/1000C	26 SCB/1000C			AFM	NA			
	LCB	NA	NA	RT	NA					
	Add.	NA	NA			Mechanical charact.	NA			
	MFI	1.4	2.05	Comp.	20/80; 30/70; 50/50; 70/30; 80/20	Others	NA			
	$\eta_{0}\;(T)$	NA	NA							

**Table 17:** Crystallization and mechanical properties of LLDPE/ULDPE blends.

LLDPE/ULDPE										
Study Number	Material Information			Processing Conditions		Crystalization and Mechanical Properties		Results and Discussion		Ref.
	Info.	Polymer 1 LLDPE	Polymer 2 ULDPE					Observation	Authors Conclusion	
S1	ρ	0.902	0.870	Pr.	DSM twin-screw extruder	DSC	A	DSC and SAXS: Depending on the composition: 2 melting peaks AFM: Nucleating effect from interface phase	Partial Miscibility	[18]
	$\bar{M_{w}}$	104,000	150,000			SSA	NA			
	$\bar{M_{n}}$	50,000	75,000	T	220	POM	NA			
	PDI	NA	NA			TEM	NA			
	Cat.	metallocene	NA							
	Com.	Octene	Octene	RPM	100	SAXS	A			
	SCB	25 SCB/1000C	60 SCB/1000C			AFM	A			
	LCB	X	X	RT	10					
	Add.	NA	NA			Mechanical charact.	NA			
	MFI	NA	NA	Comp.	25/75; 50/50; 75/25	Others	DMTA			
	$\eta_{0}\;(T)$	78 at 180 °C	22							

**Table 18:** Crystallization and mechanical properties of HDPE/UHMWPE blends.

HDPE/UHMWPE										
Study Number	Material Information			Processing Conditions		Crystalization and Mechanical Properties		Results and Discussion		Ref.
	Info.	Polymer 1 HDPE	Polymer 2 UHMWPE					Observation	Authors Conclusion	
S16	ρ	0.96	0.92	Pr.	Standard Leistritz ZSE-18 twin-screw extruder	DSC	A	DSC: One single melting peak for all the blend composition Increase in Tm, could indicate re-crystallization (co-crystllization) % of crystallization decrease with increasing HDPE content Tensile properties: Good interaction between HDPE and UHMWPE: tensile strength of blends is higher than the pure resins Impact properties: Sane conclusion than tensile tests	Miscibility (co-crystallization)	[37]
	$\bar{M_{w}}$	78,000	5,000,000			SSA	NA			
	$\bar{M_{n}}$	NA	NA	T	180–170	POM	NA			
	PDI	NA	NA			TEM	NA			
	Cat.	NA	NA							
	Com.	NA	NA	RPM	80	SAXS	NA			
	SCB	NA	NA			AFM	NA			
	LCB	NA	NA	RT	NA					
	Add.	NA	NA			Mechanical charact	A			
	MFI	7.6	No measurable	Comp.	HDPE/UHMWPE 5/95; 10/90; 20/80; 30/70; 40/60; 50/50	Others	NA			
	$\eta_{0}\;(T)$	NA	NA							
S17	ρ	0.961	0.937	Pr.	Haake Rheomix 600 mixer	DSC	A	DSC: 2 melting peaks for the blends	Immiscible (phase separation)	[36]
	$\bar{M_{w}}$	74,000	2,500,000			SSA	NA			
	$\bar{M_{n}}$	NA	NA	T	190	POM	NA			
	PDI	NA	NA			TEM	NA			
	Cat.	NA	NA							
	Com.	NA	NA	RPM	NA	SAXS	NA			
	SCB	NA	NA			AFM	NA			
	LCB	NA	NA	RT	10					
	Add.	NA	NA			Mechanical charact	NA			
	MFI	7.2	Not measurable	Comp.	HDPE/UHMWPE 90/10; 80/20; 70/30; 60/40	Others	NA			
	$\eta_{0}\;(T)$	NA	NA							
S28	ρ	0.948	NA	Pr.	Melt blending in Brabender Mixer	DSC	A	DSC and SSA: UHMWPE act as a nucleation agent Addition of UHMWPE result in larger lamella thickness	Partial Miscibility	[53]
	$\bar{M_{w}}$	NA	3.500,000			SSA	A			
	$\bar{M_{n}}$	NA	NA	T	165	POM	A			
	PDI	NA	NA			TEM	NA			
	Cat.	NA	NA							
	Com.	NA	NA	RPM	45	SAXS	NA			
	SCB	NA	NA			AFM	NA			
	LCB	NA	NA	RT	7					
	Add.	NA	NA			Mechanical charact.	NA			
	MFI	0.04	NA	Comp.	20/80; 30/70; 50/50; 70/30; 80/20	Others				
	$\eta_{0}\;(T)$	NA	NA							

**Table 19:** Crystallization and mechanical properties of LLDPE/ULDPE blends.

LLDPE/ULDPE										
Study Number	Material Information			Processing Conditions		Crystalization and Mechanical Properties		Results and Discussion		Ref.
	Info.	Polymer 1 LLDPE	Polymer 2 UHMWPE					Observation	Authors Conclusion	
S17	ρ	0.918	0.937	Pr.	Haake Rheomix 600 mixer	DSC	A	2 melting peaks for the blends	Immiscible (phase separation)	[36]
	$\bar{M_{w}}$	96,000	2,500,000			SSA	NA			
	$\bar{M_{n}}$	NA	NA	T	190	POM	NA			
	PDI	NA	NA			TEM	NA			
	Cat.	NA	NA							
	Com.	NA	NA	RPM	NA	SAXS	NA			
	SCB	NA	NA			AFM	NA			
	LCB	NA	NA	RT	10					
	Add.	NA	NA			Mechanical charact	NA			
	MFI	2.0	Not measurable	Comp.	LLDPE/UHMWPE 90/10; 80/20; 70/30; 60/40	Others	NA			
	$\eta_{0}\;(T)$	NA	NA							

**Table 20:** Crystallization and mechanical properties of LDPE/ULDPE blends.

LDPE/ULDPE										
Study Number	Material Information			Processing Conditions		Crystalization and Mechanical Properties		Results and Discussion		Ref.
	Info.	Polymer 1 LDPE	Polymer 2 UHMWPE					Observation	Authors Conclusion	
S17	ρ	0.918	0.937	Pr.	Haake Rheomix 600 mixer	DSC	A	2 melting peaks for the blends	Immiscible (phase separation)	[36]
	$\bar{M_{w}}$	96,000	2,500,000			SSA	NA			
	$\bar{M_{n}}$	NA	NA	T	190	POM	NA			
	PDI	NA	NA			TEM	NA			
	Cat.	NA	NA							
	Com.	NA	NA	RPM	NA	SAXS	NA			
	SCB	NA	NA			AFM	NA			
	LCB	NA	NA	RT	10					
	Add.	NA	NA			Mechanical charact	NA			
	MFI	2.0	Not measurable	Comp.	LLDPE/UHMWPE 90/10; 80/20; 70/30; 60/40	Others	NA			
	$\eta_{0}\;(T)$	NA	NA							

**Table 21:** Crystallization trends in polyethylene blends.

Polymer Pair	Main Trend	Key Influencing Parameters	References
HDPE/LDPE	Conflicting results 1–3 crystal phases depending on structure	Cooling rate, blend composition	[10,20,42,49]
HDPE/LLDPE	Conflicting results 1–3 crystal phases depending on structure	Comonomer type, % of branches, cooling rate	[10,18,19,32,39,52,54,64,65,80,81]
LDPE/LLDPE	Some co-crystallization occurs regardless of apparent melt miscibility	Catalyst type (m-LLDPE and Zn-LLDPE), structures	[19,26,34,48]
With UHMWPE	Alters HDPE nucleation and crystal growth despite immiscibility	Molar mass, % UHMWPE	[36,37,53]
Ternary Blends	LDPE enhances spacing and toughness; fine co-crystals within HDPE matrix	Blend ratio, catalyst, branch content.	[51]

**Table 22:** Main changes in polyethylene resulting from service life exposure and reprocessing.

Lifetime or Recycling Exposure	Main Changes in PE	Likely Effect When Blended with Virgin PE	Ref.
**Service-life exposure**			
Photodegradation and exposure to oxygen	Formation of hydroxyl and carbonyl groups; chain scission; possible branching or crosslinking	Changes in molecular-weight distribution and polarity may modify the viscosity ratio, relaxation behavior, interfacial interactions between the components of the blends as well as their crystallization behavior.	[103,104,105,106,107,108]
Mechanical loading and abrasion	Crack initiation, fragmentation, and localized chain damage	Broader property distribution and possible particulate contamination	[106,107,108]
Crosslinking during use	Network or gel formation	Incomplete melting, poor dispersion, and difficult mechanical recycling.	[104,105,106,107,108]
Additive exudation	Loss or depletion of antioxidants, slip agents, antistatic agents, UV stabilizers, and other additives	Lower oxidative stability and changes in affinity with second component of blend and in crystallization.	[104,105,109]
**Contamination**			
Polymer contamination	Presence of other polymers, especially polyolefins such as PP, as well as labels, adhesives, and multilayer residues	Additional phases in the blend; changes in rheology, morphology, interfacial adhesion, and crystallization; compatibilizers may be less effective than in equivalent virgin blends.	[98,110,111,112,113,114]
Contact with food, detergents, oils, or chemicals	Absorbed low-molar-mass compounds, odors, swelling, or surface contamination	Possible changes in viscosity ratio, relaxation behavior, interfacial interactions between the components of the blends as well as crystallization behavior.	[111,112,113]
Inorganic contamination	Paper, glass, pigments, fillers, metals, dirt, and ash	Heterogeneous nucleation and crystallization; local stress concentrations; altered rheology	[111,114,115]
**Reprocessing**			
Repeated extrusion	Thermomechanical chain scission, oxidation, branching, recombination, crosslinking, and sometimes microgel formation	Changes in molecular weight distribution and polarity may modify the viscosity ratio, relaxation behaviour, interfacial interactions between the components of the blends as well as their crystallization behavior.	[102,116,117]
Washing and thermal recycling	Removal of dirt and part of the inorganic fraction; possible loss of stabilizers during washing and subsequent thermal processing	Reduced ash content, limited change in viscosity in some cases, but possible reduction in thermo-oxidative stability.	[115]

**Table 23:** Practical decision framework for selecting a suitable virgin PE grade for blending with PCR PE.

Practical Question	Experimental Methods	Informative Outcome
Is the PCR PE degraded?	FTIR, SEC/GPC, rheology, gel-fraction measurements, and OIT/OOT	Oxidation, changes in molecular-weight distribution, chain scission, branching, crosslinking, gel formation, and residual oxidative stability
Does the PCR PE contain other polymers or contaminants?	FTIR, Raman spectroscopy, DSC, microscopy, ash-content analysis, and elemental analysis	Presence and nature of polymeric, organic, or inorganic contaminants
Does the PCR PE contain several PE grades?	DSC/SSA, Raman spectroscopy, NMR, and chemometric analysis	Identification and, when possible, quantification of the different PE fractions
Is the selected virgin PE apparently miscible with the PCR PE in the melt?	SAOS, additivity rule, Cole-Cole plots, time-temperature superposition, Han plots, and van Gurp-Palmen plots	Apparent melt miscibility, thermorheological behavior, and evidence of melt heterogeneity
How does the blend solidify?	DSC/SSA, WAXS/SAXS, microscopy, and rheology	Crystallization, co-crystallization, phase separation, crystalline structure, and phase morphology

## Data Availability

No new data were created or analyzed in this study. Data sharing is not applicable to this article.
